# Rice antioxidants: phenolic acids, flavonoids, anthocyanins, proanthocyanidins, tocopherols, tocotrienols, *γ*-oryzanol, and phytic acid

**DOI:** 10.1002/fsn3.86

**Published:** 2014-01-21

**Authors:** Piebiep Goufo, Henrique Trindade

**Affiliations:** CITAB—Centre for the Research and Technology of Agro-Environment and Biological Sciences, Universidade de Trás-os-Montes e Alto Douro5001-801, Vila Real, Portugal

**Keywords:** Antioxidants, phenolic compounds, phytic acid, rice, vitamin E, *γ*-oryzanol

## Abstract

Epidemiological studies suggested that the low incidence of certain chronic diseases in rice-consuming regions of the world might be associated with the antioxidant compound contents of rice. The molecules with antioxidant activity contained in rice include phenolic acids, flavonoids, anthocyanins, proanthocyanidins, tocopherols, tocotrienols, *γ*-oryzanol, and phytic acid. This review provides information on the contents of these compounds in rice using a food composition database built from compiling data from 316 papers. The database provides access to information that would have otherwise remained hidden in the literature. For example, among the four types of rice ranked by color, black rice varieties emerged as those exhibiting the highest antioxidant activities, followed by purple, red, and brown rice varieties. Furthermore, insoluble compounds appear to constitute the major fraction of phenolic acids and proanthocyanidins in rice, but not of flavonoids and anthocyanins. It is clear that to maximize the intake of antioxidant compounds, rice should be preferentially consumed in the form of bran or as whole grain. With respect to breeding, *japonica* rice varieties were found to be richer in antioxidant compounds compared with *indica* rice varieties. Overall, rice grain fractions appear to be rich sources of antioxidant compounds. However, on a whole grain basis and with the exception of *γ*-oryzanol and anthocyanins, the contents of antioxidants in other cereals appear to be higher than those in rice.

## Introduction

Antioxidants are defined as organic molecules that promote health by protecting the body's cells from damage caused by free radicals and reactive oxygen species that may otherwise exert harmful metabolic effects. It has been more than 20 years since Ramarathnam et al. (1989a,b1989b) first identified the flavonoid isovitexin, *α*-tocopherol, and *γ*-oryzanol in rice as having antioxidant activities comparable to that of butylated hydroxyanisole, a common food preservative. This was followed by the identification and quantitation of *γ*-oryzanol and vitamin E components in rice bran oil (Rogers et al. 1993), anthocyanin components in red rice (Terahara et al. 1994), and phenolic acids in various rice varieties (Harukaze et al. 1999). Furthermore, some studies highlighted the dual role of phytic acid as a Fe chelator and an antioxidant (Marfo et al. 1990; Lee et al. 1997). However, it was only by 2000, after Hudson et al. (2000) established a positive relationship between the lower incidence of cancers and coronary heart diseases in Asian populations and rice consumption, that a boost in research interest in rice antioxidants was observed. As a result, the research output on rice antioxidants increased rapidly with over 1000 articles primarily based on the subject being published between 2000 and 2013, representing more than a 15-fold increase compared to the 1980s and 1990s. Currently, rice is the most studied cereal in animal and human clinical trials and in food fortification (Fardet et al. 2008). This trend is likely to increase in the near future as Europe, South America, and Africa are also becoming interested in the antioxidant potentials of their rice varieties.

Although review papers have kept pace with the high number of publications, they have thus far only focused on the pharmacological properties of rice antioxidant extracts (e.g., Cicero and Gaddi 2001; Fardet et al. 2008; Walter and Marchesan 2011) and not their composition and contents. However, for nutritional epidemiology, it is useful to know which particular antioxidant has been identified in rice before selecting biomarkers of antioxidant intake. Rice antioxidants have also been demonstrated to exhibit antioxidant activities in a content-dependent manner (Cicero and Gaddi 2001). Therefore, it is necessary to have access to quantitative data about the level of each individual antioxidant compound. This in turn would enable more accurate calculations of the dietary intakes of different populations.

In this review article, areas of research related to quantifying antioxidant compounds in rice are highlighted. First, a database of the contents of six classes of antioxidants (phenolic acids, flavonoids, anthocyanins and proanthocyanidins, tocopherols and tocotrienols, *γ*-oryzanol, and phytic acid) found in rice was constructed based on the published literature. This information was then used to describe differences in rice antioxidants depending on the ease of extraction (extractable and nonextractable), grain fraction (whole grain, bran, husk, and endosperm), grain color (brown, purple, black, and red), and grain type (*japonica* and *indica*). Rice antioxidants were then compared with those of seven other cereals. Finally, the variability in the contents observed was documented with respect to factors such as cultivar, preharvest factors, storage conditions, and methods of extraction and analysis.

## Construction of the Food Composition Database of Rice Antioxidants

In 2010, the authors' research group started a study on the antioxidant composition of Portuguese rice varieties. As the importance of analytical methods for obtaining reliable data has been recognized, four methods that have been widely satisfactorily used and deemed appropriate for extracting and analyzing antioxidant compounds from rice were optimized. For extraction of phytic acid, 2.4% HCl was used, followed by purification on an anion-exchange column and reverse phase high-performance liquid chromatography (HPLC) based on the method of Frank et al. (2009). For simultaneous separation of the eight vitamin E isomers and *γ*-oryzanol, soxhlet extraction of the oil followed by normal phase-HPLC was used based on the method of Sookwong et al. (2007). A multipurpose reverse phase-HPLC method based on that developed by Hirawan et al. (2011) was used for the simultaneous separation of phenolic acids, flavonoids, and anthocyanins. To measure the antioxidant activities of rice extracts, seven assays were selected: total phenolic content (TPC), total flavonoid content (TFC), total anthocyanin content (TAC), 2,2 diphenyl-1-picrylhydrazyl (DPPH) radical scavenging activity, oxygen radical absorbance capacity (ORAC), and ferric ion reducing antioxidant power (FRAP) (Hirawan et al. 2011; Saikia et al. 2012). Pigmented and nonpigmented rice varieties grown under different environmental conditions were analyzed for contents of antioxidants in the whole grain, the endosperm, the bran, and the husk.

The need for an available and easily accessible compilation of rice composition data for use for comparisons with varieties grown in Portugal was immediately evident at the initiation of the study. Therefore, Citation databases were searched for research papers containing the terms RICE or CEREAL in their titles, plus one of the following terms: PHENOLIC, POLYPHENOL, FLAVONOID, ANTHOCYANIN, PROANTHOCYANIDIN, CONDENSED TANNIN, VITAMIN E, TOCOPHEROL, TOCOTRIENOL, ORYZANOL, FERULATE, PHYTIC ACID, PHYTATE, BIOACTIVE, BIOACTIVITY, ANTIOXIDANT, and ANTIOXIDATIVE. Over 1000 papers were downloaded based on this keyword search, and 316 papers were selected that primarily reported the composition or contents of antioxidant compounds (Data S1).

All data used in the database were original analytical values (dry weight basis). Data were scrutinized to ensure that values related to processing (fermentation, irradiation, soaking, cooking, toasting, parboiling, and extrusion) were not included. Therefore, the reported values were all indicative of fresh samples (on-farm samples) or stored samples (retail samples). To ensure that the final mean values included in the food composition tables were representative of the contents reported by the authors, data from published studies were weighted and extracted either as a selection (highest, middle, and lowest values) or as an average as proposed by Greenfield and Southgate (2003). Using these guidelines, the antioxidant composition database was built (Data S1). Data sources in the database are identified by rice cultivar, country of origin, color, and reference. The analytical methods associated with each reference are shown in Data S2. For each individual compound, the mean of all the values included in the database was calculated, and is presented along with its standard deviation as a measure of variability (Tables 1–10). Due to the drastic differences in the contents of phenolic compounds among different fractions of rice and rice varieties of different colors, data were first stratified into the rice endosperm, bran, whole grain, and husk, and then into the nonpigmented rice varieties (brown rice varieties) and pigmented rice varieties (purple, black, and red rice varieties).

**Table 1:** Contents of phenolic acids in nonpigmented rice (mg/100 g DW).^1^

	Rice endosperm			Rice bran			Rice whole grain			Rice husk		
	Soluble	Insoluble	Total	Soluble	Insoluble	Total	Soluble	Insoluble	Total	Soluble	Insoluble	Total
Gallic acid	0.02–0.19 (*n* = 4)	0.32 (*n* = 1)		0.05–0.48 (*n* = 5)	0.21–2.09 (*n* = 2)		0.04–0.65 (*n* = 7)	0.51–1.60 (*n* = 3)		0.04–0.07 (*n* = 2)	0.07–0.15 (*n* = 3)	
	0.08 ± 0.08	0.32 ± 0.00	0.40 ± 0.08	0.20 ± 0.15	1.15 ± 1.33	1.35 ± 1.48	0.21 ± 0.22	1.16 ± 0.58	1.38 ± 0.79	0.05 ± 0.01	0.15 ± 0.00	0.20 ± 0.01
Protocatechuic acid	0.01–0.17 (*n* = 4)	0.17 (*n* = 1)		0.33–1.07 (*n* = 4)	0.04–0.72 (*n* = 3)		0.04–0.24 (*n* = 4)	0.17–1.38 (*n* = 2)		0.15–0.21 (*n* = 2)	0.04–0.08 (*n* = 3)	
	0.06 ± 0.05	0.17 ± 0.00	0.23 ± 0.05	0.61 ± 0.34	0.34 ± 0.35	0.94 ± 0.69	0.12 ± 0.09	0.78 ± 0.86	0.90 ± 0.95	0.18 ± 0.04	0.06 ± 0.00	0.24 ± 0.04
*p*-Hydroxyben-zoic acid	0.01–0.05 (*n* = 5)	ND (*n* = 2)		0.25–1.39 (*n* = 5)	0.06–6.73 (*n* = 5)		0.02–0.25 (*n* = 9)	0.2–0.74 (*n* = 4)		0.11–0.53 (*n* = 2)	0.03–5.26 (*n* = 3)	
	0.02 ± 0.02	ND	NA	0.62 ± 0.53	3.08 ± 3.54	3.71 ± 4.06	0.10 ± 0.08	0.39 ± 0.27	0.49 ± 0.35	0.32 ± 0.30	2.65 ± 3.70	2.97 ± 4.00
Vanillic acid	0.02–0.15 (*n* = 6)	0.03 (*n* = 1)		0.28–1.64 (*n* = 7)	0.02–0.34 (*n* = 3)		0.04–0.40 (*n* = 13)	0.10–1.10 (*n* = 5)		0.7–1.92 (*n* = 5)	0.02–11.85 (*n* = 3)	
	0.05 ± 0.05	0.03 ± 0.00	0.08 ± 0.05	0.73 ± 0.51	0.13 ± 0.18	0.86 ± 0.69	0.19 ± 0.12	0.35 ± 0.42	0.54 ± 0.54	0.94 ± 0.56	5.94 ± 8.37	6.88 ± 8.93
Syringic acid	0.01–0.19 (*n* = 5)	0.01–0.05 (*n* = 2)		0.07–0.58 (*n* = 4)	0.08–0.21 (*n* = 2)		0.02–0.49 (*n* = 11)	0.14–1.04 (*n* = 5)		0.08–0.15 (*n* = 2)	0.11–30.32 (*n* = 3)	
	0.05 ± 0.08	0.03 ± 0.03	0.08 ± 0.11	0.22 ± 0.24	0.15 ± 0.09	0.36 ± 0.34	0.17 ± 0.16	0.37 ± 0.38	0.55 ± 0.53	0.12 ± 0.05	15.22 ± 21.36	15.33 ± 21.41
Chlorogenic acid	0.02–0.06 (*n* = 3)	0.04–0.06 (*n* = 2)		0.11–1.55 (*n* = 5)	0.21 (*n* = 1)		0.02–0.44 (*n* = 7)	0.04 (*n* = 1)		0.05–0.31 (*n* = 2)	0.02–0.04 (*n* = 3)	
	0.04 ± 0.02	0.04 ± 0.00	0.08 ± 0.02	0.84 ± 0.28	0.21 ± 0.00	1.05 ± 0.28	0.14 ± 0.04	0.04 ± 0.00	0.18 ± 0.04	0.18 ± 0.18	0.03 ± 0.00	0.21 ± 0.18
Caffeic acid	0.02–0.15 (*n* = 4)	0.02 (*n* = 1)		0.14–0.79 (*n* = 7)	0.04–0.15 (*n* = 3)		0.11–0.51 (*n* = 9)	0.22–0.61 (*n* = 3)		0.18–0.53 (*n* = 5)	0.02 (*n* = 1)	
	0.06 ± 0.06	0.02 ± 0.00	0.08 ± 0.06	0.37 ± 0.21	0.09 ± 0.05	0.46 ± 0.26	0.25 ± 0.14	0.37 ± 0.21	0.61 ± 0.35	0.30 ± 0.13	0.02 ± 0.00	0.32 ± 0.13
*p*-Coumaric acid	0.02–0.09 (*n* = 7)	0.17–0.89 (*n* = 8)		0.10–1.57 (*n* = 13)	8.10–74.20 (*n* = 6)		0.07–1.15 (*n* = 15)	0.36–2.88 (*n* = 9)		0.85–2.17 (*n* = 5)	40.6–636.7 (*n* = 6)	
	0.05 ± 0.02	0.51 ± 0.27	0.55 ± 0.30	0.87 ± 0.50	41.33 ± 20.42	42.20 ± 20.92	0.41 ± 0.32	1.21 ± 0.91	1.62 ± 1.23	1.86 ± 0.55	335.1 ± 362.7	337.0 ± 363.3
Sinapic acid	0.01–0.05 (*n* = 3)	0.06 (*n* = 1)		0.04–0.60 (*n* = 4)	0.20–3.47 (*n* = 5)		0.08–0.35 (*n* = 4)	1.42–2.28 (*n* = 2)		0.02–0.24 (*n* = 2)	0.15–0.35 (*n* = 3)	
	0.03 ± 0.02	0.06 ± 0.00	0.09 ± 0.02	0.29 ± 0.26	1.77 ± 1.42	2.06 ± 1.68	0.23 ± 0.13	1.85 ± 0.65	2.08 ± 0.73	0.11 ± 0.12	0.16 ± 0.00	0.27 ± 0.12
Ferulic acid	0.37–2.78 (*n* = 10)	0.79–9.35 (*n* = 9)		1.37–8.69 (*n* = 13)	11.9–225.4 (*n* = 9)		0.26–3.24 (*n* = 16)	2.50–25.90 (*n* = 8)		0.41–1.85 (*n* = 5)	12.7–203.7 (*n* = 6)	
	1.04 ± 0.72	4.61 ± 2.76	5.65 ± 3.48	4.21 ± 2.38	116.1 ± 81.0	120.3 ± 83.3	1.14 ± 0.85	10.34 ± 7.32	11.48 ± 8.17	1.26 ± 0.58	108.6 ± 104.4	109.9 ± 105.0
Cinnamic acid	0.01 (*n* = 1)	0.03 (*n* = 1)		0.02 (*n* = 1)	0.07 (*n* = 1)		0.10–0.29 (*n* = 3)	0.07 (*n* = 1)		ND (*n* = 2)	ND (*n* = 1)	
	0.01 ± 0.00	0.03 ± 0.00	0.04 ± 0.00	0.02 ± 0.00	0.07 ± 0.00	0.09 ± 0.00	0.23 ± 0.11	0.07 ± 0.00	0.30 ± 0.11	ND	ND	ND
Ellagic acid				4.23 (*n* = 1)			0.55 (*n* = 1)			4.34 (*n* = 1)		
	NA	NA	NA	4.23 ± 0.00	NA	NA	0.55 ± 0.00	NA	NA	4.34 ± 0.00	NA	NA
TPC-HPLC^2^	1.49 ± 1.20	5.82 ± 3.95	7.30 ± 5.15	13.21 ± 5.40	164.5 ± 108.4	177.6 ± 113.8	3.74 ± 2.26	16.93 ± 11.60	20.68 ± 12.86	9.66 ± 2.52	467.9 ± 500.5	477.6 ± 503.0
TPC-FCT^3^	3.00–50.60 (*n* = 26)	10.00–84.00 (*n* = 3)		129.0–980 (*n* = 64)	191.1–545.0 (*n* = 5)		24.0–252.4 (*n* = 69)	12.3–200.0 (*n* = 16)		61.4–320.4 (*n* = 5)	211.3–2789.8 (*n* = 6)	
	18.04 ± 12.03	38.91 ± 39.56	56.95 ± 51.60	294.1 ± 162.6	302.2 ± 145.8	596.3 ± 308.5	102.6 ± 68.6	161.3 ± 52.2	263.9 ± 120.9	138.4 ± 71.5	460.7 ± 352.6	599.2 ± 424.2

ND, not detected; NA, not available.

1 For each parameter, the first row values describe the minimum and maximum values (A–B) and the total number of studies from which data were extracted (*n*), whereas the second row values show the mean and SD.

2 TPC-HPLC is the sum of all 12 individual phenolic acids.

3 TPC is the total phenolic content (mg gallic acid equivalent/100 g) as determined using the Folin-Ciocalteu assay.

**Table 2:** Contents of phenolic acids in pigmented (black + purple + red) rice (mg/100 g DW).^1^,^2^

	Rice endosperm			Rice bran			Rice whole grain			Rice husk		
	Soluble	Insoluble	Total	Soluble	Insoluble	Total	Soluble	Insoluble	Total	Soluble	Insoluble	Total
Gallic acid	ND (*n* = 3)			1.18–8.30 (*n* = 7)	1.69–4.06 (*n* = 4)		0.23–6.44 (*n* = 6)			ND (*n* = 1)		
	ND	NA	NA	3.26 ± 1.78	2.17 ± 0.61	5.43 ± 2.40	2.00 ± 1.32	NA	NA	ND	NA	NA
Protocatechuic acid				1.98–9.70 (*n* = 7)	6.04–24.50 (*n* = 5)		0.68–4.50 (*n* = 10)	0.01–4.48 (*n* = 6)				
	NA	NA	NA	3.81 ± 2.70	12.42 ± 3.42	16.23 ± 6.12	2.50 ± 0.83	0.57 ± 0.06	3.07 ± 0.89	NA	NA	NA
*p*-Hydroxyben-zoic acid	ND (*n* = 1)			1.25–26.43 (*n* = 12)	4.00–52.52 (*n* = 5)		0.10–5.99 (*n* = 22)	0.19–1.25 (*n* = 11)		0.46–1.96 (*n* = 4)		
	ND	NA	NA	10.70 ± 4.87	20.51 ± 6.90	31.21 ± 11.77	1.35 ± 1.60	0.78 ± 0.40	2.14 ± 2.00	0.91 ± 0.30	NA	NA
Vanillic acid	ND (*n* = 2)			1.09–16.60 (*n* = 8)	7.20 (*n* = 1)		0.30–4.23 (*n* = 16)	0.20–17.70 (*n* = 12)		0.45–1.94 (*n* = 5)		
	ND	NA	NA	15.17 ± 13.68	39.64 ± 0.00	54.81 ± 13.68	2.35 ± 1.05	4.15 ± 3.55	6.50 ± 4.61	1.11 ± 0.34	NA	NA
Syringic acid				0.07–5.59 (*n* = 3)			0.37–4.49 (*n* = 11)	0.52–1.71 (*n* = 6)				
	NA	NA	NA	2.38 ± 0.63	NA	NA	1.90 ± 1.20	1.23 ± 0.34	3.13 ± 1.54	NA	NA	NA
Chlorogenic acid	ND (*n* = 1)			9.79–15.4 (*n* = 3)			1.6–3.98 (*n* = 2)			1.94 (*n* = 1)		
	ND	NA	NA	12.51 ± 2.81	NA	NA	2.79 ± 1.68	NA	NA	1.94 ± 0.00	NA	NA
Caffeic acid	0.07 (*n* = 1)			2.78–25.28 (*n* = 6)	11.1 (*n* = 1)		0.09–1.06 (*n* = 9)	0.01–0.05 (*n* = 6)		0.21–0.39 (*n* = 6)		
	0.07 ± 0.00	NA	NA	14.50 ± 3.53	11.10 ± 0.00	25.60 ± 3.53	0.38 ± 0.42	0.03 ± 0.01	0.40 ± 0.44	0.43 ± 0.16	NA	NA
*p*-Coumaric acid	0.96 (*n* = 1)	0.54 (*n* = 1)		1.64–6.20 (*n* = 14)	16.77–144.00 (*n* = 6)		0.30–5.23 (*n* = 22)	1.05–15.05 (*n* = 15)		1.44–2.27 (*n* = 6)		
	0.96 ± 0.00	0.54 ± 0.00	1.50 ± 0.00	3.05 ± 1.30	43.13 ± 41.43	46.18 ± 42.73	1.49 ± 1.50	5.13 ± 2.98	6.62 ± 4.48	1.74 ± 0.27	NA	NA
Sinapic acid	1.92 (*n* = 1)			0.65–5.00 (*n* = 6)	15.02–25.91 (*n* = 5)		4.72–16.07 (*n* = 2)	5.51–9.69 (*n* = 6)				
	1.92	NA	NA	2.32 ± 1.57	20.44 ± 4.23	22.76 ± 5.80	10.40 ± 8.03	7.07 ± 1.46	17.46 ± 9.49	NA	NA	NA
Ferulic acid	2.56–4.44 (*n* = 2)	1.68 (*n* = 1)		2.95–19.15 (*n* = 14)	15.32–94.85 (*n* = 6)		0.46–8.34 (*n* = 24)	5.73–39.5 (*n* = 15)		0.43–0.93 (*n* = 24)		
	3.50 ± 0.96	1.68 ± 0.00	5.18 ± 0.96	8.02 ± 5.75	88.60 ± 33.28	96.61 ± 39.03	2.42 ± 1.88	29.27 ± 7.98	31.68 ± 9.87	0.65 ± 0.13	NA	NA
Cinnamic acid							0.29–3.84 (*n* = 4)					
	NA	NA	NA	NA	NA	NA	1.86 ± 1.57	NA	NA	NA	NA	NA
Ellagic acid	ND (*n* = 2)	NA (*n* = 2)		5.51–12.21 (*n* = 2)			0.49–1.26 (*n* = 2)			3.43–3.54 (*n* = 2)		
	ND	NA	NA	6.11 ± 8.63	NA	NA	0.63 ± 0.89	NA	NA	1.77 ± 2.50	NA	NA
TPC-HPLC^3^	6.45 ± 0.96	2.22 ± 0.00	8.67 ± 0.96	81.83 ± 47.02	238.0 ± 89.7	319.8 ± 136.7	30.10 ± 21.97	48.20 ± 16.78	78.30 ± 38.75	8.55 ± 3.70	NA	NA
TPC-FCT^4^	5.40–52.32 (*n* = 16)	29.35–79.00 (*n* = 4)		277.2–9850.0 (*n* = 58)	227.0–8400.2 (*n* = 7)		100.1–1640.6 (*n* = 106)	37.62–665.1 (*n* = 29)		117.0–145.0 (*n* = 6)		
	21.90 ± 8.51	42.85 ± 9.80	64.76 ± 18.32	2297.4 ± 1917.6	1212.1 ± 1189.0	3509.9 ± 3107.7	365.7 ± 254.9	134.5 ± 110.3	500.3 ± 365.2	125.8 ± 10.9	NA	NA

ND, not detected; NA, not available.

1 For phenolic acid contents in black, purple, and red rice, see Table S1.

1 For each parameter, the first row values describe the minimum and maximum values (A–B) and the total number of studies from which data were extracted (*n*), whereas the second row values show the mean and SD.

1 TPC-HPLC is the sum of all 12 individual phenolic acids.

1 TPC is the total phenolic content (mg gallic acid equivalent/100 g) as determined using the Folin-Ciocalteu assay.

**Table 3:** Contents of flavonoids in rice (mg/100 g DW).^1^

Color^2^	Rice endosperm			Rice bran			Rice whole grain			Rice husk		
	Soluble	Insoluble	Total	Soluble	Insoluble	Total	Soluble	Insoluble	Total	Soluble	Insoluble	Total
Luteolin												
Pigmented	ND (*n* = 1)	ND (*n* = 1)		18.40 (*n* = 1)	ND (*n* = 1)		0.30 (*n* = 1)	ND (*n* = 1)		ND (*n* = 1)	ND (*n* = 1)	
Pigmented	ND	ND	ND	18.40 ± 0.00	ND	ND	0.30 ± 0.00	ND	ND	ND	ND	ND
Apigenin												
Nonpigmented	ND (*n* = 1)	ND (*n* = 1)		0.04 (*n* = 1)	0.32 (*n* = 1)		0.25–2.05 (*n* = 2)	0.08 (*n* = 2)		0.75–1.58 (*n* = 3)	0.10 (*n* = 1)	
Nonpigmented	ND	ND	ND	0.04 ± 0.00	0.32 ± 0.00	0.36 ± 0.00	1.15 ± 1.27	0.08 ± 0.00	1.23 ± 1.27	1.08 ± 0.00	0.10 ± 0.00	1.18 ± 0.00
Pigmented	ND (*n* = 1)	ND (*n* = 1)		7.80 (*n* = 1)	ND (*n* = 1)		0.20–2.85 (*n* = 8)	ND (*n* = 1)		ND (*n* = 1)	ND (*n* = 1)	
Pigmented	ND	ND	ND	7.80 ± 0.00	ND	ND	1.76 ± 0.81	ND	ND	ND	ND	ND
Tricin												
Nonpigmented	ND (*n* = 1)	0.02–0.05 (*n* = 3)		0.50–4.86 (*n* = 3)	0.65–2.85 (*n* = 3)		0.02–0.09 (*n* = 3)	0.62–1.77 (*n* = 3)		26.77–42.63 (*n* = 3)	1.55–3.95 (*n* = 3)	
Nonpigmented	ND	0.05 ± 0.00	ND	2.96 ± 2.69	0.32 ± 0.00	3.28 ± 2.69	0.09 ± 0.00	0.64 ± 0.00	0.73 ± 0.00	34.85 ± 0.00	3.55 ± 0.00	38.40 ± 0.00
Pigmented				11.9–193.1 (*n* = 3)								
Pigmented	NA	NA	NA	101.7 ± 90.54	NA	NA	NA	NA	NA	NA	NA	NA
Quercetin												
Pigmented	ND (*n* = 1)			1.42–6.32 (*n* = 8)			0.42–3.68 (*n* = 8)			ND (*n* = 3)		
Pigmented	ND	NA	NA	3.37 ± 1.90	NA	NA	1.96 ± 1.00	NA	NA	ND	NA	NA
Kaempferol												
Pigmented							0.37–1.05 (*n* = 7)					
Pigmented	NA	NA	NA	NA	NA	NA	0.67 ± 0.26	NA	NA	NA	NA	NA
Isorhamnetin												
Pigmented				0.04–0.67 (*n* = 5)								
Pigmented	NA	NA	NA	0.19 ± 0.27	NA	NA	NA	NA	NA	NA	NA	NA
Myricetin												
Pigmented							0.30–0.40 (*n* = 3)					
Pigmented	NA	NA	NA	NA	NA	NA	0.35 ± 0.05	NA	NA	NA	NA	NA
TFC^3^												
Nonpigmented	14.0–106.0 (*n* = 9)	35.00–65.04 (*n* = 2)		100.0–1168.1 (*n* = 15)	106.3–350.4 (*n* = 4)		30.0–293.0 (*n* = 18)	24.0–126.4 (*n* = 3)		63.96–372.0 (*n* = 7)	78.4–135.8 (*n* = 3)	
Nonpigmented	56.53 ± 33.20	50.02 ± 21.24	106.5 ± 54.4	409.9 ± 341.4	166.9 ± 67.7	576.8 ± 409.1	117.9 ± 40.3	70.47 ± 51.85	188.4 ± 92.16	205.9 ± 114.9	108.4 ± 0.0	314.3 ± 114.9
Pigmented	25.0–111.0 (*n* = 7)	6.00 (*n* = 1)		120.0–2716.7 (*n* = 29)	126.7–386.9 (*n* = 6)		108.1–590.0 (*n* = 28)	33.70–222.0 (*n* = 5)		292.0–431.0 (*n* = 6)		
Pigmented	83.96 ± 26.31	6.00 ± 0.00	89.96 ± 26.31	1107.4 ± 676.3	294.9 ± 37.8	1402.3 ± 714.2	218.7 ± 103.8	112.2 ± 42.7	330.9 ± 146.5	364.5 ± 42.3	NA	NA
Black	25.0–105.0 (*n* = 4)	6.00 (*n* = 1)		1346.4–1980.0 (*n* = 8)	126.7–386.9 (*n* = 4)		142.9–590.0 (*n* = 10)	173.0–222.0 (*n* = 2)		344.0–400.0 (*n* = 3)		
Black	76.25 ± 35.15	6.00 ± 0.00	82.25 ± 35.15	1272.6 ± 485.1	253.9 ± 113.5	1526.0 ± 598.6	279.8 ± 131.5	197.5 ± 34.6	477.3 ± 166.2	391.6 ± 44.1	NA	NA
Red	97.7–111.0 (*n* = 3)			1270.4–2716.7 (*n* = 16)	283.0 (*n* = 1)		108.7–473.0 (*n* = 13)	33.7–166.0 (*n* = 2)		292.0–370.0 (*n* = 3)		
Red	91.67 ± 17.47	NA	NA	1041.4 ± 916.7	283.0 ± 0.00	1324.7 ± 916.7	225.1 ± 136.3	99.85 ± 93.55	325.2 ± 229.8	337.3 ± 40.5	NA	NA
Purple				651.0–7110.4 (*n* = 5)	348.0 (*n* = 1)		125.0–228.1 (*n* = 5)	39.41 (*n* = 1)				
Purple	NA	NA	NA	1007.4 ± 627.2	348.0 ± 0.0	1355.2 ± 627.2	151.1 ± 43.5	39.41 ± 0.00	190.5 ± 43.5	NA	NA	NA

ND, not detected; NA, not available.

1 For each parameter, the first row values describe the minimum and maximum values (A–B) and the total number of studies from which data were extracted (*n*), whereas the second row values show the mean and SD.

2 Pigmented rice refer to rice with black, purple, and red bran, whereas nonpigmented rice refer to rice with brown bran.

3 TFC is the total flavonoid content (mg catechin equivalent/100 g) as determined using the aluminum chloride method.

**Table 4:** Contents of anthocyanins and proanthocyanidins in rice (mg/100 g DW).^1^

Color^2^	Rice endosperm	Rice bran		Rice whole grain	Rice husk
	Soluble	Soluble	Insoluble	Soluble	Soluble
Peonidin-3-*O*-glucoside					
Nonpigmented		0.04–2.61 (*n* = 3)			
Nonpigmented	NA	0.96 ± 1.43	NA	NA	NA
Pigmented		11.4–534.1 (*n* = 14)		2.9–162.1 (*n* = 15)	
Pigmented	NA	123.9 ± 102.6	NA	54.59 ± 54.75	NA
Cyanidin-3-*O*-glucoside					
Nonpigmented		7.36 (*n* = 1)			
Nonpigmented	NA	7.36 ± 0.00	NA	NA	NA
Pigmented		9.1–2640.4 (*n* = 13)		0.8–784.3 (*n* = 15)	
Pigmented	NA	700.2 ± 632.7	NA	93.42 ± 71.32	NA
Cyanidin-3-*O*-galactoside					
Nonpigmented					
Nonpigmented	NA	NA	NA	NA	NA
Pigmented		2.93–50.00 (*n* = 4)			
Pigmented	NA	28.29 ± 19.78	NA	NA	NA
Cyanidin-3-*O*-rutinoside					
Nonpigmented		6.19 (*n* = 1)			
Nonpigmented	NA	6.19 ± 0.00	NA	NA	NA
Pigmented		3.17–96.62 (*n* = 6)		13.78–19.90 (*n* = 2)	
Pigmented	NA	55.51 ± 16.75	NA	16.84 ± 0.00	NA
Catechin					
Nonpigmented		0.55–2.35 (*n* = 4)		0.26–0.44 (*n* = 4)	1.23–1.90 (*n* = 3)
Nonpigmented	NA	1.38 ± 0.74	NA	0.34 ± 0.07	1.54 ± 0.34
Pigmented		5.83–48.72 (*n* = 7)		0.40–3.98 (*n* = 8)	1.57–3.16 (*n* = 4)
Pigmented	NA	20.90 ± 6.85	NA	1.32 ± 1.03	2.02 ± 0.41
Epicatechin					
Nonpigmented				0.34 (*n* = 1)	3.12–7.49 (*n* = 3)
Nonpigmented	NA	NA	NA	0.34 ± 0.00	4.98 ± 2.26
Pigmented		46.53 (*n* = 1)		0.42–1.41 (*n* = 2)	0.57–6.14 (*n* = 6)
Pigmented	NA	46.53 ± 0.00	NA	0.92 ± 0.00	2.74 ± 0.62
TAC^3^					
Nonpigmented		3.09 (*n* = 1)	0.41 (*n* = 1)	2.00–3.26 (*n* = 3)	
Nonpigmented	NA	3.09 ± 0.00	0.41 ± 0.00	2.75 ± 0.67	NA
Pigmented	17.51 (*n* = 1)	2.7–6353.8 (*n* = 22)	4.86–8.20 (*n* = 4)	4.1–256.5 (*n* = 16)	
Pigmented	17.51 ± 0.00	1589.2 ± 1438.8	6.12 ± 1.69	59.43 ± 7.79	NA
Black	17.51 (*n* = 1)	113.5–5096.4 (*n* = 9)	4.86–8.20 (*n* = 3)	19.7–256.5 (*n* = 16)	
Black	17.51 ± 0.00	1884.1 ± 1794.0	6.66 ± 1.69	138.6 ± 18.9	NA
Red	1.50 (*n* = 1)	2.78–26.45 (*n* = 8)	5.57 (*n* = 1)	0.26–11.13 (*n* = 10)	
Red	1.50 ± 0.00	8.78 ± 8.84	5.57 ± 0.00	4.07 ± 4.46	NA
Purple		155.0–6353.4 (*n* = 5)		35.64 (*n* = 1)	
Purple	NA	2874.2 ± 2511.5	NA	35.64 ± 0.00	NA
TPAC^4^					
Nonpigmented		2.24–6.44 (*n* = 2)		5.02 (*n* = 1)	
Nonpigmented	NA	4.34 ± 2.97	NA	5.02 ± 0.00	NA
Pigmented		8.8–2261.0 (*n* = 9)	ND (*n* = 1)	5.1–202.1 (*n* = 7)	
Pigmented	NA	440.0 ± 462.9	ND	54.85 ± 38.70	NA
Black		8.8–218.9 (*n* = 7)			
Black	NA	78.05 ± 79.39	NA	NA	NA
Red		80.0–2261.0 (*n* = 9)	ND (*n* = 1)	5.1–202.0 (*n* = 7)	
Red	NA	716.6 ± 802.9	ND	87.31 ± 77.44	NA
Purple		36.0–1260.4 (*n* = 5)		22.40 (*n* = 1)	
Purple	NA	525.4 ± 506.4	NA	22.40 ± 0.00	NA

NA, not available; ND, not detected; TAC, total anthocyanin content; TPAC, total proanthocyanidin content.

1 For each parameter, the first row values describe the minimum and maximum values (A–B) and the total number of studies from which data were extracted (*n*), whereas the second row values show the mean and SD.

2 Pigmented rice refer to rice with black, purple, and red bran, whereas nonpigmented rice refer to rice with brown bran.

3 TAC in mg cyanidin-3-*O*-glucoside equivalent/100 g.

4 TPAC in mg catechin equivalent/100 g.

**Table 5:** Contents of tocopherols and tocotrienols in rice (mg/kg DW).^1^

Color^2^	Rice endosperm	Rice bran	Rice whole grain	Rice husk
*α*-Tocopherol				
Nonpigmented	0.17–5.43 (*n* = 11)	7.34–107.7 (*n* = 33)	2.40–49.14 (*n* = 32)	0.06–2.13 (*n* = 4)
Nonpigmented	1.52 ± 1.01	42.11 ± 25.91	16.61 ± 12.87	0.90 ± 0.98
Pigmented	0.33–3.78 (*n* = 7)	9.67–116.6 (*n* = 21)	4.53–18.99 (*n* = 8)	0.24–2.57 (*n* = 6)
Pigmented	1.61 ± 1.16	43.33 ± 24.53	10.14 ± 4.97	1.09 ± 0.99
*β*-Tocopherol				
Nonpigmented	0.02–0.42 (*n* = 4)	1.92–25.00 (*n* = 12)	0.08–7.60 (*n* = 8)	0.01–0.41 (*n* = 4)
Nonpigmented	0.18 ± 0.17	9.70 ± 8.48	2.08 ± 2.74	0.18 ± 0.18
Pigmented	0.15–0.26 (*n* = 6)	0.83–5.01 (*n* = 9)	0.34–1.79 (*n* = 7)	0.03–0.33 (*n* = 6)
Pigmented	0.19 ± 0.05	2.42 ± 1.15	0.92 ± 0.45	0.13 ± 0.13
*γ*-Tocopherol				
Nonpigmented	0.04–4.95 (*n* = 8)	2.60–58.90 (*n* = 32)	1.40–27.50 (*n* = 28)	0.04–0.91 (*n* = 4)
Nonpigmented	1.73 ± 1.65	23.85 ± 17.13	8.23 ± 7.14	0.41 ± 0.38
Pigmented	0.42–2.04 (*n* = 6)	9.10–57.18 (*n* = 18)	1.28–8.91 (*n* = 8)	0.08–3.49 (*n* = 6)
Pigmented	1.43 ± 0.58	27.43 ± 14.29	5.10 ± 2.71	1.17 ± 1.49
*δ*-Tocopherol				
Nonpigmented	0.02–0.15 (*n* = 5)	0.26–6.96 (*n* = 21)	0.03–4.69 (*n* = 18)	0.02–0.17 (*n* = 4)
Nonpigmented	0.11 ± 0.07	2.06 ± 1.50	1.46 ± 1.39	0.09 ± 0.07
Pigmented	0.08–0.38 (*n* = 6)	0.97–4.30 (*n* = 14)	0.35–2.38 (*n* = 6)	0.11–0.95 (*n* = 6)
Pigmented	0.17 ± 0.11	1.93 ± 0.94	0.91 ± 0.79	0.37 ± 0.31
Total tocopherols				
Nonpigmented	0.21–10.90 (*n* = 15)	16.11–218.0 (*n* = 36)	3.00–105.5 (*n* = 35)	0.18–3.34 (*n* = 4)
Nonpigmented	3.54 ± 2.90	77.72 ± 53.02	28.38 ± 24.14	1.58 ± 1.61
Pigmented	0.99–6.34 (*n* = 6)	24.30–190.1 (*n* = 18)	7.24–34.33 (*n* = 8)	0.46–8.54 (*n* = 6)
Pigmented	3.40 ± 1.90	75.11 ± 40.91	17.07 ± 8.92	2.76 ± 2.92
*α*-Tocotrienol				
Nonpigmented	0.12–8.45 (*n* = 7)	8.44–81.97 (*n* = 24)	0.77–21.38 (*n* = 25)	0.03–0.17 (*n* = 4)
Nonpigmented	1.31 ± 1.18	36.61 ± 21.05	6.79 ± 6.07	0.10 ± 0.06
Pigmented	0.62–4.44 (*n* = 7)	9.12–138.2 (*n* = 13)	2.03–11.41 (*n* = 7)	0.11–0.95 (*n* = 6)
Pigmented	1.96 ± 1.42	46.58 ± 42.91	6.60 ± 3.34	0.50 ± 0.37
*β*-Tocotrienol				
Nonpigmented	0.04–0.86 (*n* = 4)	2.40–26.00 (*n* = 10)	0.50–3.28 (*n* = 7)	0.10–0.57 (*n* = 3)
Nonpigmented	0.39 ± 0.35	9.87 ± 7.82	1.46 ± 0.92	0.38 ± 0.25
Pigmented	0.12–0.20 (*n* = 3)	0.06–0.76 (*n* = 4)	0.10–0.12 (*n* = 3)	0.09–0.14 (*n* = 3)
Pigmented	0.16 ± 0.04	0.37 ± 0.32	0.11 ± 0.01	0.12 ± 0.03
*γ*-Tocotrienol				
Nonpigmented	2.54–17.13 (*n* = 7)	17.36–212.3 (*n* = 20)	8.60–44.86 (*n* = 23)	0.03–1.75 (*n* = 4)
Nonpigmented	8.35 ± 4.79	110.8 ± 52.03	21.94 ± 9.88	0.80 ± 0.73
Pigmented	3.97–24.82 (*n* = 7)	75.98–230.6 (*n* = 13)	17.51–46.95 (*n* = 7)	0.12–11.59 (*n* = 7)
Pigmented	10.50 ± 6.90	115.9 ± 48.79	27.88 ± 10.60	5.15 ± 5.39
*δ*-Tocotrienol				
Nonpigmented	0.07–1.65 (*n* = 7)	4.36–25.30 (*n* = 18)	0.44–4.06 (*n* = 20)	0.02–0.16 (*n* = 4)
Nonpigmented	0.86 ± 0.50	12.18 ± 7.10	1.61 ± 0.94	0.11 ± 0.07
Pigmented	0.28–1.06 (*n* = 6)	1.45–16.80 (*n* = 12)	0.58–4.78 (*n* = 7)	0.05–0.48 (*n* = 6)
Pigmented	0.57 ± 0.34	5.87 ± 5.70	1.46 ± 1.50	0.25 ± 0.18
Total tocotrienols				
Nonpigmented	3.85–24.49 (*n* = 9)	29.40–360.0 (*n* = 34)	11.94–76.15 (*n* = 24)	0.23–2.81 (*n* = 5)
Nonpigmented	10.91 ± 6.86	169.4 ± 88.00	31.80 ± 17.81	1.39 ± 1.11
Pigmented	5.20–31.62 (*n* = 7)	86.40–388.9 (*n* = 13)	21.55–63.75 (*n* = 7)	0.42–13.13 (*n* = 6)
Pigmented	13.19 ± 8.70	168.7 ± 97.72	36.05 ± 15.45	6.02 ± 5.97
Vitamin E				
Nonpigmented	4.06–36.83 (*n* = 9)	47.00–585.6 (*n* = 33)	14.86–186.1 (*n* = 25)	0.41–6.02 (*n* = 5)
Nonpigmented	14.45 ± 9.72	247.1 ± 141.0	60.18 ± 41.95	2.97 ± 2.72
Pigmented	6.19–38.00 (*n* = 8)	113.7–574.3 (*n* = 13)	29.11–100.40 (*n* = 9)	0.88–22.00 (*n* = 6)
Pigmented	16.59 ± 10.60	243.8 ± 138.6	53.12 ± 24.37	8.78 ± 8.89

1 For each parameter, the first row values describe the minimum and maximum values (A–B) and the total number of studies from which data were extracted (*n*), whereas the second row values show the mean and SD.

2 Pigmented rice refer to rice with black, purple, and red bran, whereas nonpigmented rice refer to rice with brown bran.

**Table 6:** Contents of steryl ferulates (*γ*-oryzanol) in rice (mg/kg DW).^1^

Color^2^	Rice endosperm	Rice bran	Rice whole grain	Rice husk
Cycloartenyl *trans*-ferulate				
Nonpigmented	8.50–22.20 (*n* = 3)	430.0–1200.7 (*n* = 5)	20.90–133.1 (*n* = 15)	
Nonpigmented	15.28 ± 6.85	806.1 ± 346.6	76.31 ± 38.83	NA
Pigmented		362.9–742.5 (*n* = 4)	30.02 (*n* = 1)	
Pigmented	NA	496.35 ± 175.07	30.02 ± 0.00	NA
24-Methylenecylcoartanyl *trans*-ferulate				
Nonpigmented	14.70–32.20 (*n* = 3)	941.5–1676.5 (*n* = 6)	40.47–259.0 (*n* = 14)	
Nonpigmented	24.12 ± 8.83	1304 ± 292.2	122.8 ± 55.54	NA
Pigmented		737.2–1490.2 (*n* = 4)	50.91 (*n* = 1)	
Pigmented	NA	1187.4 ± 428.9	50.91 ± 0.00	NA
Campesteryl *trans*-ferulate				
Nonpigmented	6.70–14.30 (*n* = 3)	190.0–658.5 (*n* = 5)	20.00–76.00 (*n* = 11)	
Nonpigmented	10.43 ± 3.80	459.1 ± 183.9	45.63 ± 17.01	NA
Pigmented		241.2–725.1 (*n* = 4)	50.14 (*n* = 1)	
Pigmented	NA	459.2 ± 199.8	50.14 ± 0.00	NA
*β*-Sitosteryl *tran*s-ferulate				
Nonpigmented	5.30–13.40 (*n* = 3)	300.1–720.9 (*n* = 5)	8.60–85.00 (*n* = 11)	
Nonpigmented	9.03 ± 4.09	455.6 ± 169.12	36.52 ± 25.85	NA
Pigmented		271.2–823.7 (*n* = 4)	10.88 (*n* = 1)	
Pigmented	NA	467.2 ± 260.9	10.88 ± 0.00	NA
Stigmasteryl *trans*-ferulate				
Nonpigmented		20.55–58.84 (*n* = 3)	4.51–12.55 (*n* = 3)	
Nonpigmented	NA	43.00 ± 19.98	7.43 ± 4.45	NA
Pigmented		60.00 (*n* = 1)	10.00 (*n* = 1)	
Pigmented	NA	60.00 ± 0.00	10.00 ± 0.00	NA
*γ*-Oryzanol				
Nonpigmented	6.11–120.7 (*n* = 24)	1030.0–8864.0 (*n* = 65)	163.00–979.00 (*n* = 66)	60.55–158.71 (*n* = 7)
Nonpigmented	49.14 ± 28.89	3176 ± 1474	413.3 ± 190.6	102.4 ± 45.39
Pigmented	69.11–625.9 (*n* = 11)	1122–9120 (*n* = 24)	150.0–885.5 (*n* = 21)	38.18–653.0 (*n* = 6)
Pigmented	231.8 ± 208.4	3174 ± 2065	474.3 ± 223.9	323.2 ± 244.0

NA, not available.

1 For each parameter, the first row values describe the minimum and maximum values (A–B) and the total number of studies from which data were extracted (*n*), whereas the second row values show the mean and SD.

2 Pigmented rice refer to rice with black, purple, and red bran, whereas nonpigmented rice refer to rice with brown bran.

**Table 7:** Contents of phytate phosphorus and other forms of phosphorus in rice (mg/g DW).^1^

Color^2^	Rice endosperm	Rice bran	Rice whole grain	Rice husk
Phytate phosphorus^3^				
Nonpigmented	0.25–1.21 (*n* = 25)	7.88–19.15 (*n* = 27)	1.52–7.11 (*n* = 69)	0.52–1.00 (*n* = 4)
Nonpigmented	0.63 ± 0.29	13.93 ± 3.72	3.02 ± 1.58	0.84 ± 0.34
Pigmented	0.31–0.58 (*n* = 4)	9.85–17.97 (*n* = 6)	3.00–4.20 (*n* = 5)	
Pigmented	0.50 ± 0.15	14.13 ± 3.31	3.50 ± 0.65	NA
Total phosphorus				
Nonpigmented	0.95–3.10 (*n* = 12)	10.58–39.43 (*n* = 9)	3.07–8.50 (*n* = 23)	0.52–1.25 (*n* = 3)
Nonpigmented	1.67 ± 0.58	21.56 ± 9.24	4.16 ± 1.44	0.90 ± 0.37
Pigmented				
Pigmented	NA	NA	NA	NA
Inorganic phosphorus				
Nonpigmented	0.03–0.07 (*n* = 6)	0.24–0.51 (*n* = 3)	0.09–0.32 (*n* = 16)	0.15 (*n* = 1)
Nonpigmented	0.05 ± 0.01	0.42 ± 0.16	0.17 ± 0.09	0.15 ± 0.00
Pigmented				
Pigmented	NA	NA	NA	NA
Cellular phosphorus				
Nonpigmented	0.64–1.08 (*n* = 5)	5.36–8.32 (*n* = 2)	0.43–1.05 (*n* = 11)	0.04 (*n* = 1)
Nonpigmented	0.99 ± 0.18	7.21 ± 2.09	0.97 ± 0.19	0.04 ± 0.00
Pigmented				
Pigmented	NA	NA	NA	NA

NA, not available.

1 For each parameter, the first row values describe the minimum and maximum values (A–B) and the total number of studies from which data were extracted (*n*), whereas the second row values show the mean and SD.

2 Pigmented rice refer to rice with black, purple, and red bran, whereas nonpigmented rice refer to rice with brown bran.

3 Phytic acid (MW = 660) was converted to phytate P (186 g [1 mol of phytic acid contains 6 mol of P, i.e., MW = 31 × 6]) by dividing by the conversion factor 3.5484 (660/186).

**Table 8:** Mean antioxidant activity values for soluble extracts of rice.^1^

Color^2^	Rice endosperm	Rice bran	Rice whole grain	Rice husk
DPPH radical scavenging activity (mmol trolox Eq/100 g)				
Nonpigmented	NA	0.87 ± 0.33 (*n* = 7)	0.28 ± 0.39 (*n* = 6)	NA
Pigmented	NA	28.32 ± 21.81 (*n* = 17)	0.65 ± 0.16 (*n* = 25)	0.40 ± 0.00 (*n* = 1)
DPPH radical scavenging activity (g trolox Eq/100 g)				
Nonpigmented	0.27 ± 0.18 (*n* = 3)	0.68 ± 0.38 (*n* = 9)	0.59 ± 0.45 (*n* = 7)	0.27 ± 0.00 (*n* = 2)
Pigmented	0.66 ± 0.00 (*n* = 2)	9.86 ± 0.00 (*n* = 4)	5.22 ± 0.42 (*n* = 6)	0.12 ± 0.00 (*n* = 1)
DPPH radical scavenging activity (EC_50_, mg/mL)				
Nonpigmented	15.06 ± 7.87 (*n* = 5)	19.49 ± 15.33 (*n* = 24)	18.45 ± 30.08 (*n* = 5)	NA
Pigmented	NA	1.17 ± 1.21 (*n* = 15)	19.92 ± 0.01 (*n* = 6)	NA
Inhibition of linoleic acid autooxidation (EC_50_, mg/mL)				
Nonpigmented	12.00 ± 0.00 (*n* = 1)	NA	11.21 ± 0.00 (*n* = 1)	NA
Pigmented	NA	NA	NA	NA
Oxygen radical absorbance capacity (mmol trolox Eq/100 g)				
Nonpigmented	0.85 ± 0.00 (*n* = 1)	47.02 ± 47.70 (*n* = 5)	1.79 ± 0.70 (*n* = 11)	NA
Pigmented	1.52 ± 0.49 (*n* = 8)	105.5 ± 80.15 (*n* = 8)	10.28 ± 9.25 (*n* = 30)	NA
Inhibition of conjugated dienes formation (EC_50_, mg/mL)				
Nonpigmented	0.26 ± 0.00 (*n* = 1)	0.41 ± 0.22 (*n* = 4)	0.52 ± 0.28 (*n* = 2)	NA
Pigmented	NA	NA	NA	NA
ABTS radical cation scavenging activity (mmol trolox Eq/100 g)				
Nonpigmented	2.44 ± 1.27 (*n* = 6)	5.24 ± 4.20 (*n* = 7)	0.65 ± 0.69 (*n* = 25)	NA
Pigmented	0.07 ± 0.02 (*n* = 4)	12.38 ± 5.44 (*n* = 7)	3.75 ± 3.10 (*n* = 27)	NA
ABTS radical cation scavenging activity (EC_50_, mg/mL)				
Nonpigmented	NA	1.70 ± 1.42 (*n* = 3)	NA	NA
Pigmented	NA	0.77 ± 0.00 (*n* = 2)	NA	NA
Reducing power (absorbance)				
Nonpigmented	0.15 ± 0.10 (*n* = 4)	0.93 ± 0.59 (*n* = 10)	0.24 ± 0.08 (*n* = 10)	0.35 ± 0.00 (*n* = 1)
Pigmented	NA	1.26 ± 0.64 (*n* = 10)	0.48 ± 0.00 (*n* = 3)	NA
Ferric ion reducing antioxidant power (mmol Fe^+^ Eq/100 g)				
Nonpigmented	0.30 ± 0.25 (*n* = 10)	3.05 ± 1.17 (*n* = 11)	0.87 ± 0.54 (*n* = 21)	2.02 ± 0.79 (*n* = 4)
Pigmented	NA	8.07 ± 3.15 (*n* = 10)	3.35 ± 2.57 (*n* = 32)	NA
Ferrous-iron chelating activity (g EDTA Eq/100 g)				
Nonpigmented	0.06 ± 0.05 (*n* = 4)	0.89 ± 0.30 (*n* = 5)	0.25 ± 0.11 (*n* = 5)	NA
Pigmented	NA	2.00 ± 0.10 (*n* = 4)	1.08 ± 0.03 (*n* = 4)	NA
Ferrous-iron chelating activity (EC_50_, mg/mL)				
Nonpigmented	5.26 ± 0.00 (*n* = 1)	0.68 ± 0.55 (*n* = 5)	4.25 ± 4.02 (*n* = 2)	0.35 ± 0.00 (*n* = 1)
Pigmented	NA	NA	NA	NA
TBARS concentration (mg TBARS/100 g)				
Nonpigmented	NA	1.08 ± 1.39 (*n* = 5)	NA	3.84 ± 0.00 (*n* = 1)
Pigmented	NA	NA	NA	NA
Hydroxyl radical scavenging activity (EC_50_, mg/mL)				
Nonpigmented	4.18 ± 2.18 (*n* = 3)	3.94 ± 1.67 (*n* = 6)	6.12 ± 1.73 (*n* = 2)	
Pigmented	NA	1.37 ± 0.79 (*n* = 15)	NA	NA
Hydrogen peroxide scavenging activity (EC_50_, mg/mL)				
Nonpigmented	NA	NA	NA	5.82 ± 0.00 (*n* = 1)
Pigmented	NA	NA	NA	NA
Superoxide anion scavenging activity (EC_50_, mg/mL)				
Nonpigmented	NA	0.96 ± 0.76 (*n* = 7)	1.22 ± 0.00 (*n* = 1)	1.56 ± 0.00 (*n* = 1)
Pigmented	NA	0.20 ± 0.22 (*n* = 15)	NA	NA
Singlet oxygen scavenging activity (EC_50_, mg/mL)				
Nonpigmented	NA	3.14 ± 2.10 (*n* = 4)	NA	NA
Pigmented	NA	0.85 ± 0.74 (*n* = 13)	NA	NA
Molybdate reduction capacity (g BHT Eq/100 g)				
Nonpigmented	NA	0.94 ± 0.49 (*n* = 8)	NA	NA
Pigmented	NA	3.03 ± 0.36 (*n* = 4)	NA	NA
Tert-butylperoxyl scavenging activity (EC_50_, mg/mL)				
Nonpigmented	NA	0.40 ± 0.06 (*n* = 4)	1.01 ± 0.00 (*n* = 1)	NA
Pigmented	NA	0.37 ± 0.24 (*n* = 13)	1.55 ± 0.00 (*n* = 2)	NA

NA, not available.

1 See Table S1 for minimum and maximum values, values for insoluble extracts, and values for soluble + insoluble extracts.

2 Pigmented rice refer to rice with black, purple, and red bran, whereas nonpigmented rice refer to rice with brown bran. See Table S1 for values for black, purple, and red rice.

**Table 9:** Contents of antioxidant compounds in rice paddy, rice germ, and rice oil.^1^

	Paddy rice	Rice germ	Rice bran oil	Rice brown oil
TPC (mg GAE/100 g)	137.0–550.0 (*n* = 3)	40.04–262.0 (*n* = 13)		
	287.3 ± 228.2	111.3 ± 27.60	NA	NA
TFC (mg CAE/100 g)	80.00–130.0 (*n* = 2)			
	105.0 ± 35.36	NA	NA	NA
DPPH (mmol/L TE/100 g)		0.86–1.45 (*n* = 3)	0.15–2.33 (*n* = 8)	
	NA	1.11 ± 0.31	1.37 ± 0.77	NA
DPPH (EC_50_, mg/mL)		1.16–2.11 (*n* = 4)	8.43–21.87 (*n* = 2)	
	NA	1.60 ± 0.50	15.15 ± 9.50	NA
*α*-Tocopherol (mg/kg)	8.28–12.70 (*n* = 2)	60.61–457.9 (*n* = 11)	26.70–592.0 (*n* = 15)	221.0–1197 (*n* = 6)
	10.49 ± 3.13	211.4 ± 149.7	209.3 ± 185.4	559.9 ± 372.5
*β*-Tocopherol (mg/kg)	1.41–2.75 (*n* = 2)	5.89–12.40 (*n* = 5)	7.00–50.00 (*n* = 9)	26.69–46.16 (*n* = 2)
	2.08 ± 0.95	ND	30.92 ± 26.46	36.43
*γ*-Tocopherol (mg/kg)	0.87–3.73 (*n* = 2)	9.36–283.32 (*n* = 8)	13.20–240.0 (*n* = 12)	27.00–65.55 (*n* = 5)
	1.32 ± 0.64	101.2 ± 91.59	106.5 ± 77.42	45.22 ± 16.40
*δ*-Tocopherol (mg/kg)	0.67–1.46 (*n* = 2)	1.61–8.32 (*n* = 3)	7.00–11.24 (*n* = 8)	7.48–14.30 (*n* = 5)
	1.07 ± 0.56	5.45 ± 3.18	9.40 ± 2.76	9.65 ± 2.71
Total tocopherols (mg/kg)	11.47–17.44 (*n* = 2)	104.4–744.1 (*n* = 6)	72.00–846.0 (*n* = 15)	280.0–1315 (*n* = 7)
	14.96 ± 5.28	318.0 ± 244.4	356.1 ± 292.0	651.2 ± 391.6
*α*-Tocotrienol (mg/kg)	3.92–7.50 (*n* = 2)	2.55–23.83 (*n* = 3)	68.10–590.0 (*n* = 11)	117.0–1114 (*n* = 5)
	5.71 ± 2.53	13.97 ± 10.42	241.2 ± 181.1	623.7 ± 376.1
*β*-Tocotrienol (mg/kg)	ND (*n* = 2)	ND (*n* = 2)	6.94–50.50 (*n* = 5)	34.18–51.48 (*n* = 3)
	ND	ND	30.64 ± 21.74	43.22 ± 8.68
*γ*-Tocotrienol (mg/kg)	5.10–6.98 (*n* = 2)	1.12–30.84 (*n* = 3)	162.0–955.1 (*n* = 13)	340.0–2105 (*n* = 6)
	5.90 ± 1.12	24.82 ± 5.65	438.2 ± 276.0	974.1 ± 686.2
*δ*-Tocotrienol (mg/kg)	0.71–1.12 (*n* = 2)	1.49–2.40 (*n* = 3)	18.70–120.0 (*n* = 13)	55.00–129.2 (*n* = 6)
	0.92 ± 0.29	1.96 ± 0.46	89.08 ± 51.99	84.48 ± 26.70
Total tocotrienols (mg/kg)	9.73–15.31 (*n* = 2)	28.44–55.00 (*n* = 4)	174.0–1584 (*n* = 13)	413.5–3401 (*n* = 6)
	12.53 ± 3.94	40.75 ± 16.53	799.1 ± 530.8	1725 ± 1097
Vitamin E (mg/kg)	23.20–31.75 (*n* = 2)	132.0–799.0 (*n* = 5)	373.0–2431 (*n* = 15)	671.0–4717 (*n* = 6)
	27.49 ± 9.22	358.8 ± 261.0	1155 ± 822.8	2376 ± 1489
*γ*-Oryzanol (mg/kg)	19.00–406.5 (*n* = 2)	532.9–1750 (*n* = 8)	3509–31,640 (*n* = 30)	8931–32,929 (*n* = 9)
	378.8 ± 39.19	1221 ± 469.7	15,049 ± 7692	16,499 ± 7683
Phytate phosphorus (mg/g)	1.07–2.02 (*n* = 2)	8.97–11.13 (*n* = 4)		
	1.47 ± 0.14	10.16 ± 0.95	NA	NA

NA, not available; ND, not detected.

1 For each parameter, the first row values describe the minimum and maximum values (A–B) and the total number of studies from which data were extracted (*n*), whereas the second row values show the mean and SD.

**Table 10:** Comparison of the antioxidant composition of whole grain rice and other whole grain cereals^1^,^2^

Parameters	Rice	Maize	Wheat	Oat	Barley	Rye	Sorghum	Millet	Rank
TPC (mg GAE/100 g)	23.97–387.0 (*n* = 16)	15.01–367.1 (*n* = 12)	21.40–595.0 (*n* = 16)	28.00–330.3 (*n* = 11)	37.69–408.3 (*n* = 8)	47.49–142.5 (*n* = 3)	49.00–241.0 (*n* = 6)	44.00–373.1 (*n* = 7)	
	144.5 ± 116.9	164.3 ± 104.3	187.3 ± 147.9	186.6 ± 118.9	194.6 ± 175.8	95.05 ± 47.54	111.0 ± 83.42	185.0 ± 106.5	6/8
TFC (mg CAE/100 G)	26.01–53.04 (*n* = 3)	26.01–102.3 (*n* = 3)	18.00–61.07 (*n* = 3)	21.01–57.04 (*n* = 3)	37.06–192.1 (*n* = 3)				
	35.36 ± 15.32	66.69 ± 38.29	40.71 ± 21.63	38.03 ± 18.10	128.05 ± 80.95	NA	NA	NA	5/5
TAC (mg CGE/100 g)	2.30–327.6 (*n* = 4)	6.94–60.71 (*n* = 3)	0.79–25.10 (*n* = 4)		3.46 (*n* = 1)				
	85.49 ± 161.4	26.94 ± 29.41	14.17 ± 11.09	NA	3.46 ± 0.00	NA	NA	NA	1/4
DPHH (mmol TE/100 g)	0.13–1.23 (*n* = 7)	0.45–1.70 (*n* = 6)	0.11–2.90 (*n* = 7)	0.24–2.60 (*n* = 5)	0.60–2.39 (*n* = 4)		1.27 (*n* = 2)	0.40–1.73 (*n* = 3)	
	0.69 ± 0.41	1.25 ± 0.44	0.75 ± 0.97	1.00 ± 0.94	1.50 ± 0.77	NA	1.27 ± 0.00	1.13 ± 0.67	7/7
ABTS (mmol TE/100 g)	0.02–2.00 (*n* = 12)	0.30–1.30 (*n* = 6)	0.13–1.78 (*n* = 7)	0.02–2.85 (*n* = 5)	0.25–3.31 (*n* = 5)	1.16–2.45 (*n* = 2)	0.04–2.40 (*n* = 4)	0.05–0.91 (*n* = 8)	
	0.75 ± 0.74	0.66 ± 0.42	0.52 ± 0.57	0.80 ± 1.17	1.06 ± 1.29	1.81 ± 0.91	0.82 ± 1.10	0.27 ± 0.30	5/8
FRAP (mmol Fe^2+^/100 g)	0.15–1.91 (*n* = 14)	0.16–1.56 (*n* = 12)	0.30–1.21 (*n* = 10)	0.23–1.84 (*n* = 8)	0.25–1.89 (*n* = 5)	2.33 (*n* = 1)	0.25–1.42 (*n* = 5)	0.13–4.71 (*n* = 6)	
	0.95 ± 0.62	0.79 ± 0.42	0.60 ± 0.36	0.98 ± 0.59	1.02 ± 0.59	2.33 ± 0.00	0.83 ± 0.46	1.51 ± 1.65	5/8
Reducing power (absorbance)	0.79–0.93 (*n* = 2)	1.20 (*n* = 2)	0.36–0.61 (*n* = 2)				0.82 (*n* = 2)	1.17–4.54 (*n* = 2)	
	0.86 ± 0.10	1.20 ± 0.00	0.49 ± 0.18	NA	NA	NA	0.82 ± 0.00	2.86 ± 2.38	3/5
ORAC (mmol TE/100 g)	1.77–3.15 (*n* = 3)	2.12–8.37 (*n* = 3)	2.06–4.83 (*n* = 3)	2.54–5.43 (*n* = 3)	2.59–7.94 (*n* = 3)				
	2.10 ± 0.93	5.58 ± 3.18	3.22 ± 1.44	3.62 ± 1.58	5.29 ± 2.68	NA	NA	NA	5/5
Vitamin E (mg/kg)	20.00–102.7 (*n* = 6)	35.58–153.0 (*n* = 4)	40.00–230.0 (*n* = 6)	19.35–280.0 (*n* = 5)	34.00–80.80 (*n* = 2)	38.00–190.0 (*n* = 4)		27.00–86.41 (*n* = 3)	
	51.58 ± 35.28	79.57 ± 54.85	102.2 ± 72.33	104.2 ± 112.3	57.40 ± 33.09	108.3 ± 72.29	NA	59.34 ± 30.05	7/7
*γ*-Oryzanol (mg/kg)	200.0–720.0 (*n* = 3)	30.00–220.0 (*n* = 3)	62.00–126.0 (*n* = 3)		2.00–4.00 (*n* = 3)	29.00–86.00 (*n* = 3)			
	473.3 ± 261.0	140.0 ± 98.49	95.33 ± 32.08	NA	2.67 ± 1.15	57.67 ± 28.50	NA	NA	1/5
Phytate P (mg/g)	0.16–3.05 (*n* = 13)	0.23–2.55 (*n* = 10)	0.33–2.05 (*n* = 9)	1.34–3.15 (*n* = 5)	1.50–2.39 (*n* = 6)	0.52–2.49 (*n* = 5)	0.59–2.89 (*n* = 10)	0.33–3.09 (*n* = 9)	
	1.21 ± 0.87	1.65 ± 0.86	1.25 ± 0.70	2.19 ± 0.68	1.74 ± 0.40	1.40 ± 0.71	2.05 ± 0.92	1.89 ± 1.00	8/8

NA, not available.

1 For each parameter, the first row values describe the minimum and maximum values (A–B) and the total number of studies from which data were extracted (*n*), whereas the second row values show the mean and SD.

2 With the exception of TAC, all other values refer to nonpigmented rice.

## Antioxidant Composition of Rice

In this study, the antioxidant compounds in rice were classified into six groups: phenolic acids, flavonoids, anthocyanins and proanthocyanidins, tocopherols and tocotrienols (vitamin E), *γ*-oryzanol, and phytic acid. The first three groups are referred to collectively as phenolic compounds.

### Phenolic acid composition of rice

Phenolic acids are substances containing a phenolic ring and an organic carboxylic acid function (Goufo et al. 2014b), with absorption maxima at 280 nm for the C6-C1 skeleton of hydroxybenzoic acid derivatives (gallic, protocatechuic, *p*-hydroxybenzoic, vanillic, and syringic acids) and at 320 nm for the C6-C3 skeleton of hydroxycinnamic acid derivatives (*p*-coumaric, ferulic, caffeic, sinapic, chlorogenic, and cinnamic acids). The phenolic ring can stabilize and delocalize unpaired electrons, conferring an antioxidant property to phenolic acids. The antioxidant property notably depends on the number and the position of hydroxyl groups on the phenolic ring (Goffman and Bergman 2004; Chung and Shin 2007; Heuberger et al. 2010). Twelve phenolic acids are usually identified in rice, with their sum ranging from 7.3 to 8.7 mg/100 g in the endosperm, 177.6 to 319.8 mg/100 g in the bran, 20.8 to 78.3 mg/100 g in the whole grain, and 477.6 mg/100 in the husk, depending on the rice color (Tables 1 and 2). The most abundant phenolic acid found in the endosperm, bran, and whole grain is ferulic acid (56–77% of total phenolic acids), followed by *p*-coumaric acid (8–24%), sinapic acid (2–12%), gallic acid (1–6%), protocatechuic acid (1–4%), *p*-hydroxybenzoic acid (1–2%), vanillic acid (1%), and syringic acid (1%). Minor constituents are caffeic, chlorogenic, cinnamic, and ellagic acids, each accounting for less than 1% of total phenolic acids. In the husk, a different ranking was observed, with *p*-coumaric acid being the dominant phenolic acid (71%), followed by ferulic acid (23%), vanillic acid (3%), and syringic acid (1%). This ranking is consistent with the values reported in most studies (Harukaze et al. 1999; Tian et al. 2004; Zhou et al. 2004; Vichapong et al. 2010; Sompong et al. 2011; Tuncel and Yılmaz 2011; Chen et al. 2012a; Deng et al. 2012; Huang and Ng 2012; Irakli et al. 2012; Jun et al. 2012; Mohanlal et al. 2012; Moongngarm et al. 2012; Goufo et al. 2014a). In KDML 105 rice, however, Butsat et al. (2009) found *p*-hydroxybenzoic acid to be the major phenolic acid in the husk, accounting for 42% of the total phenolic acids, followed by ferulic acid (24%), and *p*-coumaric acid (12%). Overall, phenolic acids in rice are composed of 61–89% hydroxycinnamic acids and 12–28% hydroxybenzoic acids. Several other phenolic acids have recently been identified in rice, but await quantification or confirmation in other studies. These include methoxycinnamic acid (Chen et al. 2012a), ethyl-3,4-dihydroxybenzoic acid, 4-hydroxy-3-methoxyphenylacetic acid, 3,4-dihydroxybenzoic acid, 4-hydroxy-3-methoxy cinnamic acid (Chung and Shin 2007), 4-hydroxy-3-methoxy methyl benzoic acid, 3,4-dihydroxy methyl benzoic acid, *p*-methoxyphenol (Fujita et al. 2010), guaiacol, *o*-cresol, 3,5-xylene (Vichapong et al. 2010), *p*-cresol (Vichapong et al. 2010; Chen et al. 2012a), 6′-*O*-(E)-feruloylsucrose, 6′-*O*-(E)-sinapoylsucrose (Tian et al. 2004; Finocchiaro et al. 2007), feruloyl quinic acid, sinapoyl rutinoside, and sinapoyl tartrate (Finocchiaro et al. 2007).

### Flavonoid composition of rice

Similar to phenolic acid, flavonoids are synthesized by the phenylpropanoid metabolic pathway. Most flavonoids have absorption maxima at 370 nm. Flavonoids consist of a 15-carbon skeleton that is organized in two aromatic rings (A-and B-rings) interlinked by a three-carbon chain (structure C6-C3-C6). Flavonoids are recognized for both their ability to donate electrons and to stop chain reactions. These activities are attributed to the phenolic hydroxyls, particularly in the 3′OH and 4′OH of the three-carbon chain (Ramarathnam et al. 1989a; Hudson et al. 2000; Kim et al. 2010; Cho et al. 2013). Flavonoids can be classified into flavones, flavonols, flavanols (flavan-3-ols), flavanonols, isoflavones, and flavanones, which generally occur as *O*-or *C*-glycosides. In nonpigmented rice varieties, flavones are the most commonly encountered flavonoids. Although phenolic acids have been extensively studied in rice, few papers have focused on flavonoids (Chi et al. 2007; Hirawan et al. 2011; Chen et al. 2012a; Deng et al. 2012; Irakli et al. 2012; Goufo et al. 2014a; Sriseadka et al. 2013). Of the seven flavonoids that are usually reported in rice, tricin appears to be the major flavonoid in the bran, accounting for 77% of all seven flavonoids (131.5 mg/100 g) (Table 3). The other flavonoids are present in the following order: luteolin (14%) > apigenin (6%) > quercetin (3%) > isorhamnetin (1%) > kaempferol (<1%) > myricetin (<1%). The other flavonoids that have been recently identified in rice but not yet quantified or confirmed in other studies include tricin 4′-*O*-*(erythro-β*-guaiacylglyceryl) ether, tricin 4′*O*-(*threo-β*-guaiacylglyceryl) ether (Mohanlal et al. 2012), isovitexin (Ramarathnam et al. 1989a), naringenin (Chen et al. 2012a; Irakli et al. 2012), hesperidin (Chi et al. 2007; Chen et al. 2012a; Irakli et al. 2012), rutin (Irakli et al. 2012), luteolin-7-*O*-glucoside, apigenin-7-*O*-glucoside (Goufo et al. 2014a), quercetin-3-*O*-glucoside (Irakli et al. 2012; Sriseadka et al. 2013), quercetin-3-*O*-rutinoside, isorhamnetin-3-*O*-glucoside, isorhamnetin-3-*O*-acetylglucoside, isorhamnetin-7-*O*-rutinoside, taxifolin-7-*O*-glucoside, 5,3′,4′,5′-tetrahydroxyflavanone-7-*O*-glucoside, 5,6,3′,4′,5′-pentahydroxyflavone-7-*O*-glucoside, myricetin-7-*O*-glucoside (Sriseadka et al. 2013), apigenin-6-*C*-glucoside-8-*C*-arabinoside (Hirawan et al. 2011), (+)-3′-*O*-methyltaxifolin, brassicin, isorhamnetin-4′-*O*-glucoside, 3′-*O*-methyltaxifolin-5-*O*-glucoside, 3′-*O*-methyltaxifolin-7-*O*-glucoside, 3′-*O*-methyltaxifolin-4′-*O*-glucoside, isorhamnetin-7-*O*-cellobioside (brassicin-4′'-*O*-*β*-d-glucoside), and brassicin-4′-*O*-glucoside (Cho et al. 2013).

### Anthocyanin and proanthocyanidin composition of rice

Anthocyanins, another class of flavonoids, which exhibit maximum absorbance in the green/blue spectrum at 510 nm, are water-soluble glycosides of polyhydroxyl and polymethoxyl derivatives of 2-phenylbenzopyrylium or flavylium (2-phenylchromenylium) salts. They share a common hydroxylation at the C3, C5, and C7 positions on the B-ring. Anthocyanins exist as *O*-glycosides (mono, di, or tri) and acylglycosides of anthocyanidins in plants. The sugars may be substituted by aliphatic, hydroxybenzoic, or hydroxycinnamic acids. The structural characteristic of anthocyanins makes them highly reactive toward reactive oxygen species (Zhang et al. 2006; Fardet et al. 2008; Sam et al. 2008; Sangkitikomol et al. 2010; Pitija et al. 2013). About 18 anthocyanins have been identified in rice, of which only four have been quantified (cyanidin-3-*O*-glucoside, peonidin-3-*O*-glucoside, cyanidin-3-*O*-rutinoside, and cyanidin-3-*O*-galactoside). The mean value of the sum of the four anthocyanins in pigmented rice varieties is 1252.7 mg/100 g and 345.8 mg/100 g for the bran and the whole grain, respectively (Table 4). The anthocyanin content of rice varies more widely than does the phenolic acid content. Despite that great variability, a survey of over 25 studies (e.g., Terahara et al. 1994; Ryu et al. 1998; Ichikawa et al. 2001; Abdel-Aal et al. 2006; Zhang et al. 2006; Sam et al. 2008; Hiemori et al. 2009; Min et al. 2011; Yoshimura et al. 2011; Chen et al. 2012b) indicated that cyanidin-3-*O*-glucoside and peonidin-3-*O*-glucoside are the predominant anthocyanins in rice, accounting for 51–84% and 6–16% of the TAC, respectively, depending on the rice bran color and the rice fraction. The next most common anthocyanins in rice are cyanidin-3-*O*-rutinoside (3–5%) and cyanidin-3-*O*-galactoside (1–2%). The minor constituents are cyanidin-3-*O*-sophoroside, peonidin-3-*O*-rutinoside (Terahara et al. 1994), cyanidin-3,5-*O*-diglucoside (Terahara et al. 1994; Abdel-Aal et al. 2006; Zhang et al. 2006; Hiemori et al. 2009; Min et al. 2011), peonidin-3,5-*O*-diglucoside (Yoshimura et al. 2011), pelargonidin-3-*O*-glucoside (Sam et al. 2008; Yoshimura et al. 2011), delphinidin-3-*O*-glucoside (Ichikawa et al. 2001; Sam et al. 2008; Min et al. 2011), petunidin-3-*O*-glucoside (Ichikawa et al. 2001; Sam et al. 2008; Chen et al. 2012b), petunidin-3-*O*-galactoside, petunidin-3-*O*-arabinoside, delphinidin-3-*O*-galactoside, delphinidin-3-*O*-arabinoside, malvidin-3-*O*-galactoside (Ichikawa et al. 2001), malvidin-3-*O*-glucoside (Ichikawa et al. 2001; Sam et al. 2008), and pelargonidin-3,5-*O*-diglucoside (Zhang et al. 2006). The anthocyanidins peonidin, delphinidin, cyanidin (Min et al. 2011), and malvidin (Chen et al. 2012b) have also been reported in rice. Although petunidin-3-*O*-glucoside and malvidin have rarely been reported as important anthocyanins in rice, in an analysis of different rice cultivars from Japan, Chen et al. (2012b) found that petunidin-3-*O*-glucoside was the predominant anthocyanin in the rice cultivar Chinakuromai, comprising almost half of the TAC. In the cultivar Asamurasaki, malvidin accounted for 17–28% of the TAC.

Anthocyanidins are intermediates in the synthesis of proanthocyanidins. Proanthocyanidins, or condensed tannins, are a class of polymeric phenolic compounds consisting mainly of flavon-3-ol units (catechin, epicatechin, and their 3-*O*-gallates and epigallates). Catechin and epicatechin are particularly abundant in the bran (20.90 and 46.53 mg/100 g, respectively) and in the husk (2.02 and 2.74 mg/100 g, respectively) of pigmented rice varieties (Table 4). The degree of polymerization and galloylation affect the bioactivity of proanthocyanidins. On the basis of the observed molecular weights, the main proanthocyanidin compounds in rice are most likely oligomers of epicatechin linked by four to eight carbon–carbon bonds (B-types) (Finocchiaro et al. 2007; Mohanlal et al. 2012). Analyses of proanthocyanidins based on their degree of polymerization showed that monomers to trimers of lower molecular weight proanthocyanidins account for 38% of the total proanthocyanidin content (Chen et al. 2012b). The dominant components of proanthocyanidins were the molecular weights of dimers (Gunaratne et al. 2013).

### Tocopherol and tocotrienol composition of rice

Tocotrienols and tocopherols are known collectively as vitamin E or tocols as they share a common basic structural unit based on an amphiphilic 6-chromanol ring and a terpenoid side chain located at position 2 of the ring. The chromanol head group can be joined to a saturated phytyl side chain to form tocopherols, or to an unsaturated geranylgeranyl side chain to form tocotrienols. The head group can then be methylated in different configurations, resulting in four alternative forms (*α*,*β*,*γ*, and *δ*). The free hydroxyl group on the chromanol ring is responsible for the antioxidant properties, and the hydrogen atom from this group can be donated to free radicals, resulting in a resonance-stabilized vitamin E radical (Qureshi et al. 2000; Xu et al. 2001; Kim 2005). In nonpigmented rice varieties, the mean vitamin E content is 14.5, 247.2, 60.2, and 3.0 mg/kg for the endosperm, bran, whole grain, and husk, respectively (Table 5). Depending on the rice grain fraction, *γ*-tocotrienol contributes the most to the total tocol content (27–63%), followed by *α*-tocopherol (10–30%), *α*-tocotrienol (9–19%), *γ*-tocopherol (9–14%), *δ*-tocotrienol (2–6%), *β*-tocotrienol (1–4%), *β*-tocopherol (1–2%), and *δ*-tocopherol (1–2%). This ranking is similar to that inferred in most studies (Diack and Sask 1994; Yu et al. 2007; Heinemann et al. 2008; Imsanguan et al. 2008; Huang and Ng 2011; Min et al. 2011; Jeng et al. 2012; Zhang et al. 2012; Pascual et al. 2013); however, *α*-tocopherol was reported to be the major tocol in two Taiwanese rice varieties (Lin and Lai 2011). With respect to rice varieties of the Southern United States, the content of *α*-tocotrienol was highest, followed by *γ*-tocotrienol, *α*-tocopherol, and *γ*-tocopherol (Bergman and Xu 2003). A positive correlation between *γ*-tocotrienol and vitamin E content was observed, but only a loose correlation between the total tocopherol and total tocotrienol content was found (Sookwong et al. 2007). Overall, tocotrienols account for 47–80% of the total vitamin E content, and tocopherols for 20–53%. In heated rice bran, two novel tocotrienols were isolated and characterized as desmethyl and didesmethyl tocotrienols (Qureshi et al. 2000). The unsaturated tocotrienols were found to have greater antioxidant properties, which are probably related to the fact that their structures have fewer methyl groups and hence lower steric hindrance impeding their penetration into membranes.

### Steryl ferulate (γ-oryzanol) composition of rice

Gamma-oryzanol is a mixture of steryl ferulates, which are formed by esterification of the hydroxyl group of sterols (campesterol, stigmasterol, *β*-sitosterol) or triterpene alcohols (cycloartanol, cycloartenol, 24-methylenecycloartanol, cyclobranol) with the carboxylic acid group of ferulic acid (Diack and Sask 1994; Bucci et al., 2003; Yu et al. 2007; Imsanguan et al. 2008; Lu et al. 2011; Jeng et al. 2012). Sterols with a saturated steroid skeleton are known as stanols, whereas compounds containing a double bond between C5 and C6 or between C7 and C8 are referred to as sterols. Methyl groups at C4 affect the antioxidant properties of steryl ferulates (Akihisa et al. 2000; Deepam et al. 2011; Mandak and Nystrom 2012). On the basis of their absorbance maxima at 330 nm, at least 25 components of *γ*-oryzanol have been identified so far, with five of them comprising about 95% of the total *γ*-oryzanol content (Akihisa et al. 2000; Xu et al. 2001; Fang et al. 2003; Miller and Engel 2006). In nonpigmented rice varieties, the mean value of these five components is 58.9, 3067.1, and 288.6 mg/kg for the endosperm, bran, and whole grain, respectively (Table 6). The contribution of these five *γ*-oryzanol components to the total *γ*-oryzanol content is in the following decreasing order: 24-methylenecycloartanyl *trans*-ferulate (present at 34–44% of the total), cycloartenyl *trans*-ferulate (19–26%), campesteryl *trans*-ferulate (15–23%), *β*-sitosteryl *trans*-ferulate (7–17%), and stigmasteryl *trans*-ferulate (1–7%). Other components of *γ*-oryzanol include 24-methylenecylcoartanyl *cis*-ferulate, cycloartenyl *cis*-ferulate, *β*-sitosteryl *cis*-ferulate, 24-methylenecholesterol *cis*-ferulate, stigmastanyl *cis*-ferulate, *β*-sitostanyl *trans*-ferulate, Δ^7^-sitostenyl *trans*-ferulate, campestanyl *trans*-ferulate, stigmastanyl *trans*-ferulate, Δ^7^-stigmastenyl *trans*-ferulate, Δ^7^-campestenyl *trans*-ferulate, 24-hydroxy-24-methylcycloartanol *trans*-ferulate, 25-hydroxy-24-methylcycloartanol *trans*-ferulate, (24S)-cycloart-25-ene-3*β*, 24-diol-3*β*-*trans*-ferulate, (24R)-cycloart-25-ene-3*β*,24-diol-3*β*-*tran*s-ferulate, cycloart-23Z-ene-3*β*,25-diol-3*β*-*trans*-ferulate, hydroxylated cycloartenol *trans*-ferulate, 24-methylcholesterol *trans*-ferulate, cycloeucalenol *trans*-ferulate, and 24-methylenecholesterol *trans*-ferulate (Diack and Sask 1994; Akihisa et al. 2000; Xu et al. 2001; Fang et al. 2003). One caffeate ester (cycloartenyl *trans*-caffeate) has also been reported as part of *γ*-oryzanol in rice (Fang et al. 2003), but this remains to be confirmed. There is also a high probability that the five *cis*-feruloyl esters identified by Akihisa et al. (2000) were simply artifacts, since long-wavelength UV radiation observed in the laboratory can induce *cis*-*trans* isomerization of feruloyl esters. The data showed that the proportion of cycloartenyl *trans*-ferulate was negatively correlated with the proportion of 24 methylenecycloartanyl *trans*-ferulate and with that of campesteryl *trans*-ferulate. In addition, the proportion of campesteryl *trans*-ferulate was negatively correlated with the proportion of campestanyl *trans*-ferulate (Miller and Engel 2006; Mandak and Nystrom 2012; Mohanlal et al. 2012).

### Phosphorus composition of rice

Phosphorus in rice is found in three forms: inositol polyphosphate, inorganic phosphorus, and cellular phosphorus (Agte et al. 1999; Liang et al. 2007; Ren et al. 2007; Frontela et al. 2008; Frank et al. 2009; Wang et al. 2011). Cellular phosphorus comprises all other forms of organic phosphorus including DNA, RNA, free nucleotides, phospholipids, and sugar phosphates. The highest phosphorus content is found in the bran (21.56 mg/g), followed by the whole grain (4.16 mg/g), endosperm (1.67 mg/g), and husk (1.03 mg/g) (Table 7). Phytic acid, also known as phytate phosphorus or myo-inositol-1.2.3.4.5.6-hexakisphosphate (ringed myo-inositol with six phosphate groups attached to each carbon), is the most abundant form of phosphorus in the whole grain and in the bran, representing 65–73% of the total phosphorus content. Phytic acid suppresses Fe-catalyzed oxidative reactions owing to its capacity to chelate Fe^2+^ or to keep iron in its inert form (Fe^3+^) (Canan et al. 2011; Mohanlal et al. 2012). Lower inositol polyphosphates (InsP3, InsP4, and InsP5) can also be found in rice, but only in trace levels and mainly reflecting hydrolysis that occurs during storage. In the whole grain and bran, the phytate phosphorus content is highest, followed by cellular phosphorus (23–33% of the total phosphorus) and inorganic phosphorus (2–4%). In the endosperm, the cellular phosphorus content represents about 60% of the total phosphorus content, whereas phytate phosphorus accounts for only 37%. A different ranking is observed in the husk with the phytate phosphorus content being highest (82% of the total phosphorus content), followed by inorganic phosphorus (14%) and cellular phosphorus (4%).

### The antioxidant activity of rice

Sixteen methods are routinely used for assessments of the antioxidant activities of rice grains (Table 8). Depending on the reaction mechanisms upon which they operate, these methods can be classified into five groups: (1) those based on the prevention of chain initiation by rice antioxidants, including ORAC, 2,2-azinobis(3-ethylbenzothiazoline-6-sulfonic acid) (ABTS) radical cation scavenging activity, and ferrous-iron chelating activity, (2) those based on the inhibition of peroxide formation, including inhibition of linoleic acid autooxidation, (3) those based on the prevention of continued hydrogen abstraction, including inhibition of conjugated diene formation and inhibition of thiobarbituric acid reactive substance (TBARS) formation, (4) those based on reductive capacity, including reducing power, molybdate reduction capacity, FRAP, and DPPH radical scavenging activity, and (5) those based on oxy-radical scavenging, including hydrogen peroxide, hydroxyl radical, superoxide anion, singlet oxygen, *tert*-butylperoxyl, and peroxynitrite scavenging activities (Kim 2005; Chi et al. 2007; Stratil et al. 2007; Shen et al. 2009; Sangkitikomol et al. 2010; Zhang et al. 2010; Laokuldilok et al. 2011; Min et al. 2011; Jun et al. 2012; Moongngarm et al. 2012; Saikia et al. 2012; Cho et al. 2013; Gunaratne et al. 2013; Seo et al. 2013; Walter et al. 2013). Each of these assays can independently account for more than one of these mechanisms. On the other hand, the same antioxidant compound might react differently in different assays. Determining the method that can most reliably measure the antioxidant activity of rice samples is beyond the scope of this paper. It is important to note that unlike maize (Fardet et al. 2008; Sreeramulu et al. 2009), the in vitro antioxidant activities of rice are, in most cases, significantly correlated with their antioxidant compound contents.

Phenolic acids are reported to possess higher antioxidant activities than those of anthocyanins (Min et al. 2011; Chen et al. 2012a; Pitija et al. 2013). Phenolic compounds also show higher reducing power compared with *α*-tocopherol (Laokuldilok et al. 2011). It was also reported that phenolic compounds exhibit antioxidant activities that are up to four times higher than those of *α*-tocopherol (Goffman and Bergman 2004; Kim 2005; Yawadio et al. 2007) and *γ*-oryzanol (Xu et al. 2001). The antioxidant activity of the main *γ*-oryzanol components are reported to be almost 10 times higher than those of tocopherols, whereas tocotrienols show antioxidant activities that are 40–60 times greater than those of tocopherols (Xu et al. 2001; Deepam et al. 2011). Among the anthocyanins, the antioxidant activity determined using the ORAC assay showed the following ranking: peonidin-3-*O*-glucoside > malvidin > cyanidin-3-*O*-glucoside > petunidin-3-*O*-glucoside (Chen et al. 2012b). Historically, *α*-tocopherol has been considered the vitamin E isomer of greatest value due to its high level of physiological activity; however, *δ*-tocotrienol has recently been reported as having in vitro and in vivo free radical scavenging activities that are more than three times that of *α*-tocopherol (Qureshi et al. 2000; Kim 2005). Among the components of *γ*-oryzanol, the highest antioxidant activity was found for 24-methylenecycloartanyl *trans*-ferulate (Xu et al. 2001).

## Contribution of Soluble and Insoluble Compounds to the Total Antioxidant Content of Rice

Most antioxidants in rice exist in three forms, namely, soluble-free, soluble-conjugated, and insoluble. Soluble, extractable, or free antioxidants are generally low and intermediate molecular mass compounds that can be extracted using different organic and organic-aqueous solvents (e.g., methanol, ethanol). Insoluble, nonextractable, or bound antioxidants comprise compounds with high molecular mass, compounds cross-or ester-linked to various cell wall macromolecules (e.g., arabinoxylans, pectins, cellulose, lignin, proteins), and compounds trapped in the core of the food matrix. Insoluble compounds usually remain in the residues of organic extractions.

### Contribution of insoluble phenolic acids to the total phenolic acid content

As shown in Tables 1 and 2, insoluble phenolic acids account for the major part of phenolic acids in rice. For example, in nonpigmented rice varieties, insoluble phenolic acids represent approximately 68% of the TPC of the endosperm (56.9 mg gallic acid equivalent [GAE]/100 g), 51% of the TPC of the bran (596.5 mg GAE/100 g), 61% of the TPC of the whole grain (263.9 mg GAE/100 g), and 77% of the TPC of the husk (599.2 mg GAE/100 g). These values are consistent with values reported by most authors (e.g., Harukaze et al. 1999; Tian et al. 2004; Zhou et al. 2004; Butsat et al. 2009; Laokuldilok et al. 2011; Massaretto et al. 2011; Min et al. 2011; Goufo et al. 2014a). In some studies, higher contents of soluble phenolic acids have been found relative to insoluble phenolic acids (De Mira et al. 2009; Zhang et al. 2010; Tuncel and Yılmaz 2011). However, these studies used methanol combined with HCl for the extraction of soluble phenolic compounds. Part of the insoluble phenolics is released by HCl, which surely explains why the contents of soluble phenolic acids are higher than those of insoluble phenolic acids in these studies. With the exception of chlorogenic and caffeic acids, the major portion of individual phenolic acids also exists in insoluble forms in all rice fractions. For example, the percentage contribution of insoluble ferulic acid to the total ferulic acid content ranges from 88% to 99% depending on the rice fraction analyzed (Table 1). In contrast to nonpigmented rice varieties, in pigmented rice varieties, the soluble TPC is two to three times higher than the insoluble TPC in the bran and in the whole grain (Table 2). This could simply be a sampling artifact as insufficient data are available on the insoluble TPC in pigmented rice varieties to establish an accurate value.

### Contribution of insoluble flavonoids to the TFC

The data extracted from the literature appear to support the conclusion that insoluble flavonoids are present in a significant amount in the rice grain (Zhang et al. 2010; Lin and Lai 2011; Min et al. 2011), which challenges the widely accepted view that flavonoids are exclusively free in plants. In the endosperm of pigmented rice varieties, insoluble flavonoids account for 7% of the TFC = 89.9 mg catechin equivalent (CAE)/100 g, 21% in the bran (TFC = 1402.0 mg CAE/100 g), and 34% in the whole grain (TFC = 330.9 mg CAE/100 g) (Table 3). For example, in the husk, the soluble tricin content is 34.8 mg/100 g, whereas the insoluble tricin content is 3.5 mg/100 g. The exact nature of the associations between rice flavonoids and cell wall components remains to be elucidated.

### Contribution of insoluble anthocyanins and proanthocyanidins to the total anthocyanin and proanthocyanidin contents

Insoluble anthocyanins contribute only 1–12% to the TAC. For example, in the bran of pigmented rice varieties, the soluble TAC is 1589.0 mg cyanidin-3-*O*-glucoside equivalent (CGE)/100 g, whereas the insoluble TAC is 6.1 mg CGE/100 g (Table 4). This observation agrees with the literature, which shows that anthocyanins are mainly stored in the vacuole and are not bound to cell walls (Finocchiaro et al. 2007; Zhang et al. 2010). In fact, some authors reported that after acid hydrolysis of rice residues, insoluble anthocyanins may actually correspond to hydrolyzed proanthocyanidins (Finocchiaro et al. 2007). In contrast to anthocyanins, proanthocyanidins in rice appear to be mainly bound to the cell wall components or associated with proteins that cannot be easily disrupted. However, it was not until 2010 that some rice research groups turned their attention to this class of phenolic compounds (Min et al. 2011; Chen et al. 2012b; Gunaratne et al. 2013).

There have been no reports linking tocopherols, tocotrienols, steryl ferulates, and phytic acid to the cellular components of rice.

### Contribution of insoluble antioxidants to the total antioxidant activity

In agreement with the literature (Pellegrini et al. 2006; Butsat et al. 2009; Vichapong et al. 2010; Zhang et al. 2010; Min et al. 2011; Tuncel and Yılmaz 2011; Deng et al. 2012; Goufo et al. 2014a), insoluble antioxidants emerged as the major contributors to the total antioxidant activity of rice (Table S1). However, the insoluble phenolic contents are only loosely correlated with their antioxidant activities (Finocchiaro et al. 2007; Min et al. 2011; Goufo et al. 2014a). The results of a study by Goufo et al. (2014a) showed that bound phenolics were less sensitive to DPPH (*R*^2^ = 0.505 and *R*^2^ = 0.454 for the TPC and the TFC, respectively) compared to free phenolics (*R*^2^ = 0.895 and *R*^2^ = 0.886 for the TPC and the TFC, respectively). This might imply that the insoluble extract obtained after alkaline or acidic hydrolysis of rice samples contains other bioactive compounds besides phenolic acids and flavonoids. However, the matrix may include antioxidant components that react slowly or may be even inert to the DPPH radical. Therefore, more data with different assays are needed before a conclusion can be reached.

## Distribution of Antioxidant Compounds in the Rice Grain

Rice harvested from the field is known as paddy or rough rice. Milling is the process wherein the paddy is transformed into a form that is suitable for human consumption. The process starts with removing the inedible husk (or hull) that covers the grain, thereby producing the whole grain (or brown rice). The rice endosperm, also known as milled rice, polished rice, or white rice, is produced by an additional polishing process that strips the bran layer of the whole grain rice. In general, the rice endosperm is preferred over the whole grain owing to its desirable sensory properties and storage stability. The bran layer consists of the bran (pericarp, seed coat, nucellus, and aleurone) and the germ (or embryo). The rice bran, embryo, and husk are considered by-products of the rice milling industry.

### Distribution of phenolic acids in rice

The data presented in Table 1 show that the rice bran is the richest source of phenolic acids in rice, which is consistent with the literature (Tian et al. 2004; Finocchiaro et al. 2007; Vichapong et al. 2010; Tuncel and Yılmaz 2011; Gunaratne et al. 2013; Walter et al. 2013). In nonpigmented rice varieties, the bran fraction has a TPC of 596.3 mg GAE/100 g, which is close to that of the husk (599.2 mg GAE/100 g); this is followed by the whole grain (263.9 mg GAE/100 g) and the rice endosperm (56.9 mg GAE/100 g). The only exceptions are vanillic, syringic, and *p*-coumaric acids, whose contents are, respectively, 8.0, 37.0, and 7.9 times higher in the husk compared with the bran. This is consistent with *p*-coumaric and vanillic acids being primarily associated with the highly lignified cell walls of the husk, and ferulic acid ester preferentially linking with arabinoxylans, which are abundant in the walls of aleurone cells (bran) (Harukaze et al. 1999; Zhou et al. 2004).

### Distribution of flavonoids in rice

As observed for the TPC, the TFC of the bran (576.8 mg CAE/100 g and 1402.0 mg CAE/100 g for nonpigmented and pigmented rice varieties, respectively) is higher than that of the husk (1.8 times), the whole grain (3.1–4.2 times), and the endosperm (5.4–15.6 times). Surprisingly, the husk of nonpigmented rice varieties contains more tricin (38.40 vs. 3.28 mg/100 g) and apigenin (1.18 vs. 0.36 mg/100 g) than the bran (Table 3). This is consistent with the findings of Goufo et al. (2014a).

### Distribution of anthocyanins and proanthocyanidins in rice

Anthocyanin and proanthocyanidin compounds also appear to be mainly associated with the bran layer of rice (Hirawan et al. 2011; Yoshimura et al. 2011; Chen et al. 2012b). For example, in the bran of pigmented rice varieties, the TAC was found to be 1589.0 mg CGE/100 g, which is higher than that found in the whole grain (59.4 mg CGE/100 g; Table 4). The bran cyanidin-3-*O*-glucoside (700.2 mg/100 g), peonidin-3-*O*-glucoside (123.9 mg/100 g), and cyanidin-3-*O*-rutinoside (55.5 mg/100 g) contents are, respectively, 7.5, 2.3, and 3.3 times higher than those in the whole grain. The endosperm contains almost no anthocyanins or proanthocyanidins, while no data could be found for the husk.

### Distribution of tocopherols and tocotrienols in rice

Depending on the rice color, the vitamin E distribution in rice is ranked in the following decreasing order: bran (243.8–247.1 mg/kg) > whole grain (53.1–60.1 mg/kg) > endosperm (14.4–16.5 mg/kg) > husk (2.9–8.7mg/kg) (Table 5). This corroborates the findings of Finocchiaro et al. (2007), Jeng et al. (2012), and Goufo et al. (2014a); however, Huang and Ng (2011) reported a higher vitamin E content in the husk of 16 Taiwanese varieties compared with the endosperm.

### Distribution of γ-oryzanol in rice

The steryl ferulate esters that compose *γ*-oryzanol predominantly reside in the bran (Yu et al. 2007; Huang and Ng 2011; Tuncel and Yılmaz 2011; Jeng et al. 2012; Mandak and Nystrom 2012). Regardless of the rice color, the *γ*-oryzanol distribution in rice is ranked in the following decreasing order: bran (3174.2–3176.4 mg/kg) > whole grain (413.3–473.3 mg/kg) > husk (102.4–323.2 mg/kg) > endosperm (49.1–231.8 mg/kg) (Table 6).

### Distribution of phytic acid in rice

The results obtained for phytic acid (Table 7) are in agreement with previous reports (Lee et al. 1997; Liang et al. 2007; Ren et al. 2007; Wang et al. 2011), wherein approximately 90% of the phytate phosphorus is concentrated in the bran (13.93 mg/g), 4–5% in the rice endosperm (0.63 mg/g), and 4–5% in the husk (0.84 mg/g). In the whole grain, the mean content of phytate phosphorus is 3.02 mg/g for nonpigmented rice varieties.

### Antioxidant activities of rice fractions

Because the bran harbors most of the antioxidant compounds, the bran fraction shows higher values of antioxidant activities compared with the other fractions, regardless of the antioxidant activity assay used, which is usually followed by the husk, whole grain, and endosperm (Table 8). For instance, the FRAP value of nonpigmented rice varieties was calculated to be 3.05 mmol FeSO_4_/100 g for the bran, 2.02 mmol FeSO_4_/100 g for the husk, 0.87 mmol FeSO_4_/100 g for the whole grain, and 0.30 mmol FeSO_4_/100 g for the endosperm.

## Effect of Bran Color on the Antioxidant Composition of Rice

Rice is usually classified depending on the color of its bran into four groups: brown, black, purple, and red. In this study, bran fractions of brown color were defined as nonpigmented rice, whereas bran fractions of black, purple, and red color were defined as pigmented rice. The grain color is directly visible only after removing the husk to obtain the whole grain. Milling the whole grain to obtain the rice endosperm usually removes the color. Most of the commercially grown rice varieties around the world are nonpigmented (Goufo 2008). Until recently, pigmented rice varieties were cultivated only in restricted areas of the globe for ornamentation and for making specialty foods and alcoholic beverages, but are becoming increasingly popular.

### Effect of bran color on the phenolic acid composition of rice

Considerable evidence has accumulated to substantiate the claim that pigmented rice varieties are more phenolic acid-rich compared to nonpigmented rice varieties (Goffman and Bergman 2004; Chi et al. 2007; Chung and Shin 2007; Finocchiaro et al. 2007; Yawadio et al. 2007; De Mira et al. 2009; Vichapong et al. 2010; Zhang et al. 2010; Hirawan et al. 2011; Lin and Lai 2011; Massaretto et al. 2011; Huang and Ng 2012; Mohanlal et al. 2012; Gunaratne et al. 2013; Pitija et al. 2013; Seo et al. 2013). For example, in the bran, the TPC of pigmented rice varieties (sum of black, purple, and red) is 3509 mg GAE/100 g, which is 5.9 times higher than that of nonpigmented rice varieties (596.3 mg GAE/100 g). These differences are still evident in the endosperm with the colored bran removed (Tables 1 and 2). With the exception of ferulic acid, the same observation was made for individual phenolic compounds, which are 1.1 (for *p*-coumaric acid) to 63.7 times (for vanillic acid) higher in pigmented rice varieties compared with nonpigmented rice varieties. Among the pigmented rice varieties, black rice varieties have higher TPCs in all rice fractions, followed by red and purples rice varieties. For example, in the whole grain, black rice had the highest TPC (686.4 mg GAE/100 g), followed by red rice (517.6 mg GAE/100 g) and purple rice (296.8 mg GAE/100 g). The higher phenolic compound content in black rice varieties compared with red and purples rice varieties is well documented (Zhang et al. 2006; Shen et al. 2009; Sangkitikomol et al. 2010; Laokuldilok et al. 2011; Sompong et al. 2011; Deng et al. 2012; Irakli et al. 2012; Jun et al. 2012; Walter et al. 2013); however, results are contradictory with respect to the comparison between the purple and red rice varieties, with some authors reporting no difference (De Mira et al. 2009; Chen et al. 2012b), some finding a higher TPC in red rice varieties (Saikia et al. 2012), and others finding a higher TPC in purple rice varieties (Laokuldilok et al. 2011; Min et al. 2011; Chen et al. 2012a; Deng et al. 2012). However, pooling the data together revealed a net tendency for a higher TPC in red rice varieties compared with purple rice varieties, as observed for protocatechuic acid (11.08 vs. 5.77 mg/100 g) and syringic acid (0.42 vs. 0.07 mg/100 g) in the bran (Table S1).

### Effect of bran color on the flavonoid composition of rice

Up to threefold differences in TFC values were evident between pigmented and nonpigmented rice varieties (Table 3). For instance, the bran TFC is 1402.4 mg CAE/100 g for pigmented rice varieties and is 576.8 mg CAE/100 g for nonpigmented rice varieties. Significant differences were also found among pigmented rice varieties, with black rice > red rice ≥ purple rice. These results were generally consistent with the literature (Finocchiaro et al. 2007; Shen et al. 2009; Zhang et al. 2010; Min et al. 2011; Chen et al. 2012a; Huang and Ng 2012; Irakli et al. 2012; Saikia et al. 2012); however, Jun et al. (2012) reported a higher TFC in red rice varieties compared with black rice varieties. Results obtained for tricin and apigenin (Table 3) indicated that individual flavonoid contents might also be higher in pigmented rice varieties compared with nonpigmented rice varieties. Goufo et al. (2014a) reported a free tricin content of 2.96 mg/100 g in the Italian nonpigmented rice Ariete, whereas Mohanlal et al. (2012) reported a content of 101.7 mg/100 g in the Indian red rice Njavara.

### Effect of bran color on the anthocyanin and proanthocyanidin composition of rice

Among the rice bran color varieties, the TAC, ranked in descending order, is purple (2874.0 CGE/100 g), black (1884.0 CGE/100 g), red (8.78 CGE/100 g), and brown (3.09 CGE/100 g) rice (Table 4). The same ranking applies to cyanidin-3-*O*-glucoside, cyanidin-3-*O*-rutinoside, and peonidin-3-*O*-glucoside; that is, black rice ≥ purple rice > red rice > brown rice. Comparison of anthocyanin and proanthocyanidin contents revealed that black rice varieties are mainly composed of anthocyanins (Ryu et al. 1998; Yawadio et al. 2007; Zhang et al. 2010; Hirawan et al. 2011; Sompong et al. 2011; Saikia et al. 2012; Pitija et al. 2013) and red rice varieties are mainly composed of proanthocyanidins (Sangkitikomol et al. 2010; Min et al. 2011; Chen et al. 2012b; Mohanlal et al. 2012; Gunaratne et al. 2013). Consistent with this result, the bran of red rice varieties exhibited the highest total proanthocyanidin content (TPAC = 716.6 mg CAE/100 g), followed by that of purple rice varieties (525.4 mg CAE/100 g), black rice varieties (78.0 mg CAE/100 g), and brown rice varieties (4.34 mg CAE/100 g) (Table 4). In addition, purple rice varieties were found to have considerable amounts of both anthocyanins and proanthocyanidins. This indicates that anthocyanins contribute to the black color of black rice varieties, proanthocyanidins to the red color of red rice varieties, and both contribute to the purple color of purple rice varieties. Unlike anthocyanins, which are reddish to purple, proanthocyanidins are colorless. To give the red color, proanthocyanidins have to be oxidized into complex compounds such as phlobatannins and phlobaphenes (Finocchiaro et al. 2007).

### Effect of bran color on the tocopherol and tocotrienol composition of rice

Differences in tocopherol and tocotrienol contents in rice are not associated with the bran color as reported in several studies (Finocchiaro et al. 2007; Yawadio et al. 2007; Huang and Ng 2011; Min et al. 2011; Gunaratne et al. 2013), and shown in Table 5 for the endosperm, whole grain, and bran. In the husk, however, the total tocotrienol content of pigmented rice varieties (6.02 mg/kg) is 4.3-fold higher than that of nonpigmented rice varieties. This is unlikely to be due to the bran color, but is instead likely due to the low number of studies (<5) published on the subject.

### Effect of bran color on the γ-oryzanol composition of rice

The *γ*-oryzanol contents were found to vary between rice fractions, but were not related to the bran color (Huang and Ng 2011; Min et al. 2011; Mandak and Nystrom 2012; Gunaratne et al. 2013; Seo et al. 2013). For example, the bran *γ*-oryzanol contents are 3176 mg/kg and 3174 mg/kg for nonpigmented and pigmented rice varieties, respectively (Table 6).

### Effect of bran color on the phytic acid content of rice

The results presented in Table 7 show that there was no clear difference observed between the phytic acid content of nonpigmented and pigmented rice varieties: 0.63 versus 0.50 mg/g for the endosperm, 13.9 versus 14.1 mg/g for the bran, and 3.0 versus 3.5 mg/g for the whole grain. However, no study has directly compared the phytic acid content between pigmented and nonpigmented rice varieties.

### Effect of bran color on the antioxidant activity of rice

As shown in Table 8, regardless of the antioxidant assay (with the exceptions of ABTS in mmol TE/100 g endosperm, DPPM in mmol TE/100 g husk, and EC_50_ of the whole grain to scavenge tBuOO radicals), pigmented rice varieties possess higher antioxidant activities compared with nonpigmented rice varieties (Chi et al. 2007; Finocchiaro et al. 2007; Shen et al. 2009; Vichapong et al. 2010; Zhang et al. 2010; Laokuldilok et al. 2011; Chen et al. 2012b; Mohanlal et al. 2012; Moongngarm et al. 2012; Saikia et al. 2012; Gunaratne et al. 2013; Pitija et al. 2013; Seo et al. 2013; Walter et al. 2013). For example, in the whole grain, the ORAC value was 10.28 mmol TE/100 g for pigmented rice varieties, but was only 1.79 mmol TE/100 g for nonpigmented rice varieties. The data shown in Table S1 comparing the antioxidant activities of black, purple, red, and brown rice varieties suggest that ORAC and reducing power assays are mostly related to the sample content of proanthocyanidins. For all rice fractions, ORAC and reducing power values followed the order red rice > purple rice > black rice > brown rice (Table S1), which matches well with the ranking obtained with the TPAC. It is also apparent that DPPH, FRAP, Fe-chelating activity, and superoxide radical scavenging activity assays are more sensitive to the sample content of phenolic acids and flavonoids. For most rice fractions, values obtained using these assays followed the order black rice > red rice ≥ purple rice > brown rice (Table S1), which matches well with the ranking obtained with the TPC and the TFC. This observation is not surprising as different assays function through different mechanisms, and would hence yield different results depending on the type of antioxidant present in the sample. For example, the FRAP assay is based on a single-electron transfer mechanism, whereas the ORAC assay is based on hydrogen atom transfer (Fardet et al. 2008). As such, it is not unusual that the FRAP and ORAC assays may or may not correlate depending on the food system being tested.

## Effect of Rice Subspecies (*indica* and *japonica*) on the Antioxidant Composition of Rice

Rice is classified into two subspecies, *indica* and *japonica*, depending on the degree of spikelet sterility in F1 hybrids between the two types. *Indica* rice varieties are usually grown in tropical areas. They are vigorous, tall, and have large leaves and firm cooking grains. *Japonica* rice varieties are grown in temperate areas. They are more productive, short to intermediate in size, rounder, and have low amylose content and soft cooking grains (Heuberger et al. 2010).

At least five published articles support the idea that *japonica* rice varieties possess more phenolic acids and more flavonoids compared with *indica* rice varieties (Ramarathnam et al. 1989a; Harukaze et al. 1999; Heuberger et al. 2010; Zhang et al. 2010; Huang and Ng 2012). Clustering analysis based on single-nucleotide polymorphisms (SNPs) in phenolic compounds pathways could group rice varieties according to *indica* and *japonica* subspecies (Heuberger et al. 2010). Of the 10 rice varieties analyzed by Heuberger et al. (2010), the TPC of cooked rice was 288.0 mg GAE/100 g for the *japonica* rice varieties, which was statistically different from that of *indica* rice varieties (179.0 mg GAE/100 g). In Zhang et al.'s (2010) study, the average value of the TFC of *japonica* rice (six varieties) was 25% higher than that of *indica* rice (eight varieties). These authors also reported higher anthocyanin contents and ORAC values in *japonica* rice varieties compared with *indica* rice varieties. The vitamin E content (Heinemann et al. 2008; Heuberger et al. 2010; Huang and Ng 2011; Lin and Lai 2011; Zhang et al. 2012) and the *γ*-oryzanol content (Ramarathnam et al. 1989b; Heinemann et al. 2008; Huang and Ng 2011; Lu et al. 2011; Pascual et al. 2013) of *japonica* rice varieties were also reported to be higher than those of *indica* rice varieties. For example, in Brazilian rice varieties the average vitamin E content was 24.2 mg/kg for *japonica* rice varieties and was 17.1 mg/kg for *indica* rice varieties (Heinemann et al. 2008). In *japonica* rice varieties, *α*-tocopherol and *α*-tocotrienol are the most abundant vitamin E isomers, whereas in *indica* rice varieties the most abundant vitamin E isomer is *γ*-tocotrienol (Zhang et al. 2012). To the best of the authors' knowledge, no studies have investigated the relationship between the phytic acid content of rice and the classification of rice into *indica* and *japonica* subspecies.

## Contents of Antioxidants in the Paddy Rice, Rice Germ, and Rice Bran Oil

Besides the bran, three other products of the rice industry also have industrial applications, namely, the paddy, the germ, and the oil. Rice bran oil has become the most widely popularized valued-added product from the rice industry, and is used in many countries as cooking oil as well as in alternative medicine, cosmetics, and pharmaceutics (Rogers et al. 1993; Cicero and Gaddi 2001; Xu et al. 2001). Compared to other vegetable oils, rice bran oil is very stable at high temperatures. The World Health Organization recommends a composition of saturated fatty acid to monounsaturated fatty acid to polyunsaturated fatty acid of 1:1.5:1. Compared to olive oil, sunflower oil, or soybean oil, crude rice bran oil matches this composition most closely (Deepam et al. 2011). There have also been efforts to concentrate rice antioxidants directly from the paddy rice in the production of germinated rice varieties (Tian et al. 2004), parboiled rice varieties (Pascual et al. 2013; Walter et al. 2013), and the germ (Yu et al. 2007).

As shown in Table 9, tocopherols and tocotrienols are found in rice bran oil in higher quantities than those found in all rice fractions. For *γ*-oryzanol, the following ranking could be established: rice brown oil (16,499.0 mg/kg) > rice bran oil (15,049.0 mg/kg) > rice bran (3178.0 mg/kg) > rice germ (1221.0 mg/kg) > whole grain rice (413.3 mg/kg) > rice paddy (378.8 mg/kg) > rice endosperm (102.4 mg/kg) > rice husk (49.1 mg/kg). The same ranking applies to vitamin E, with one notable difference: the germ has a higher vitamin E content (358.8 mg/kg) compared with the bran (247.1 mg/kg). Moreover, it was found that the contribution of each vitamin E isomer to the total vitamin E in the rice bran oil is the same as that found in the bran, whole grain, husk, and endosperm, with *γ*-tocotrienol accounting for 38–41% of the total tocol content, followed by *α*-tocopherol (18–24%), *α*-tocotrienol (21–26%), and *γ*-tocopherol (2–9%). The major vitamin E isomer in the rice germ appears to be *α*-tocopherol (and not *γ*-tocotrienol as found for the bran), accounting for 59% of the total tocol content, followed by *γ*-tocopherol (28%), *γ*-tocotrienol (7%), and *α*-tocotrienol (4%), which is in agreement with the findings of Yu et al. (2007), Jeng et al. (2012), and Moongngarm et al. (2012). The bran contains higher levels of phytic acid compared with the germ (13.93 vs. 10.12 mg/g). Moongngarm et al. (2012) also reported higher levels of phenolic acids and flavonoids in the bran compared with the germ. In the paddy, the phytic acid, vitamin E, and *γ*-oryzanol contents are lower than those found in the whole grain, but are higher than those in the husk and the endosperm (Table 9), which is in agreement with the literature (Ren et al. 2007; Tuncel and Yılmaz 2011).

## Rice Antioxidants in the World of Cereals

Rice grain is the most popular cereal worldwide, serving as a stable food for nearly half of the world's population. It is the grain with the second highest worldwide production after maize.

Compared with other cereals, rice does not appear to be a rich source of antioxidant compounds (Table 10). The mean values presented in Table 10, although calculated using values extracted from several papers (e.g., Agte et al. 1999; Abdel-Aal et al. 2006; Pellegrini et al. 2006; Stratil et al. 2007; Sreeramulu et al. 2009; Hirawan et al. 2011; Laokuldilok et al. 2011; Min et al. 2011), are highly reliable since each paper compared rice with other cereals using exactly the same analytical methods. It is important to mention that the data presented in Table 10 refer to the sum of soluble and insoluble antioxidants in the whole grains of nonpigmented rice varieties (with the exception of anthocyanins and proanthocyanidins, whose values are given for pigmented rice varieties). With the exception of the *γ*-oryzanol and anthocyanin contents, which are highest in rice, the contents of all other antioxidant compounds are lower in rice. In general, and regardless of the method applied, the average antioxidant activity of other cereals is equal to or exceeds that of rice. Moreover, there is much difference between the consumption of white rice (endosperm) and brown rice (whole grain) in terms of antioxidant intake as the major components that possess antioxidant activity are located in rice bran.

Barley and wheat were found to have higher TPC and TFC levels than maize and oat, followed by rice and rye (Table 10). The vitamin E content in cereal grains was observed in the following order: rye (108.0 mg/kg) > oat (104.2 mg/kg) > wheat (102.2 mg/kg) > maize (79.5 mg/kg) > millet (59.3 mg/kg) > barley (57.4 mg/kg) > rice (51.5 mg/kg). Comparison of different cereals indicated that rice (1.21 mg/g) was lowest and wheat (1.25 mg/g) was second lowest with respect to phytic acid content, with sorghum (2.05 mg/g), and oat (2.19 mg/g) on the higher side of the range, which, with some exceptions, agrees with the findings of Agte et al. (1999), Fardet et al. (2008), and Frontela et al. (2008).

Steryl ferulates composing *γ*-oryzanol have been identified in rice, wheat, maize, rye, triticale, and barley. Among these cereals, rice exhibits the highest levels of steryl ferulates (Mandak and Nystrom 2012). The mean *γ*-oryzanol content in whole grain rice is 473.3 mg/kg, which is 3.4 times higher than that in maize, 5.1 times higher than that in wheat, 8.2 times higher than that in rye, and 177.3 times higher than that in barley (Table 10). As shown in Table 10, the TAC appears to be higher in pigmented rice varieties compared with other pigmented cereals. For example, Abdel-Aal et al. (2006) reported that black rice bran has the highest TAC (327.6 mg CGE/100 g) among the blue, red, and purple cereal grains, including corn, wheat, and barley (ranging from 0.67 to 127.7 mg CGE/100 g); this result was subsequently confirmed by Min et al. (2011). It is important to note that when cereals are considered independently of their colors, sorghum and black rice appear to have the highest phenolic compound contents and the highest antioxidant activities (Fardet et al. 2008).

## Variability in the Antioxidant Composition of Rice

There is notable variation in the levels of rice antioxidant compounds as shown from the high standard deviations in Tables 1–10. The main factors responsible for the large variation in the values in the literature are the genetic makeup of the different rice varieties, preharvest factors, storage conditions, and analytical methods.

### Genetic makeup

Foods are inherently variable in composition. Numerous lines of evidence have shown that secondary metabolites in plant foods are more prone to variation than primary metabolites (Greenfield and Southgate 2003), and 2/3 of the differences in the specific values observed in this study could be attributed to the genetic makeup of rice. One example is provided in a study conducted by Ryu et al. (1998), who analyzed 10 black rice varieties that were grown in the same region. Five of the grains exhibited a low TAC (10–55 mg CGE/100 g) and three had an intermediate TAC (232–266 mg CGE/100 g), whereas the rice variety Suwon 415 had a TAC of 473 mg CGE/100 g, representing a 43.7-fold difference compared with the first group of rice varieties.

### Preharvest factors

Antioxidant compounds are formed during seed maturation; therefore, differences in antioxidant contents of rice grains may be due to differences in the degree of maturation, which is in turn affected by environmental fluctuations, location, irrigation conditions, soil type, and fertilizer and pesticide applications, among other factors. For instance, there is a 1.2-fold difference in the TPC between husk samples harvested at maturity (22–28 days after flowering) and those harvested at the fully ripe stage (29–35 days after flowering) (Butsat et al. 2009). Decreases of 34% were found for the vitamin E content of whole grains from rice grown on a sandy soil compared with rice grown on a clay soil (P. Goufo, unpubl. data).

### Storage conditions

The levels of rice antioxidant compounds presented in this study also depended on storage conditions. Storage of whole rice grain at room temperature (25°C) for 6 months caused a 70% loss of vitamin E and an 18% loss of *γ*-oryzanol (Pascual et al. 2013). Storage also reduced the TPC, with the decline in phenolic acids being greater at 37°C than at 4°C (Zhou et al. 2004).

### Analytical methods

The lack of a standardized method for the extraction and analysis of antioxidants in rice may have also contributed to the wide variation observed in the data reported. Besides phytic acid, the methodologies for all of these compounds have not yet been studied in collaborative trials (Data S2).

## Extraction and Analysis of Antioxidants in Rice

### Extraction and analysis of phenolic acids

Three extraction factors can bring about variability in the contents of phenolic compounds in rice. First, several authors defat their sample before extraction, usually with hexane (Chen et al. 2012a; Chiou et al. 2013), a process that reduces the phenolic acid content (Min et al. 2011). Second, rice phenolic acids exist in both soluble and insoluble forms. The most frequently used solvent systems for the extraction of soluble phenolic acids are 70–80% ethanol or methanol (Chi et al. 2007; Chen et al. 2012a), 70–80% ethanol or methanol + HCl (Kim et al. 2010; Zhang et al. 2010), and 70% ethanol or methanol + heating at 70–80°C (Kim et al. 2010; Goufo et al. 2014a). Insoluble phenolic acids may be released by heat treatment of samples prior to or during the extraction of soluble phenolic acids. Solutions containing HCl may also cause the release of part of the insoluble phenolic acids, thus leading to higher levels of soluble phenolic compounds (Shen et al. 2009). Although the same method is generally used for the extraction of insoluble phenolic acids (NaOH + HCl + ethyl acetate) from the residues of aqueous-organic extractions (Laokuldilok et al. 2011; Lin and Lai 2011), the extraction yield may still differ depending on the solvent concentration and the extraction time selected (Pellegrini et al. 2006). Third, depending on the instruments used, reported extraction techniques of phenolic acids from rice include vortex-assisted extraction (Sompong et al. 2011; Chen et al. 2012a), ultrasonic-assisted extraction (Hirawan et al. 2011; Huang and Ng 2012), soxhlet extraction (Tuncel and Yılmaz 2011; Chiou et al. 2013), supercritical fluid extraction (Chiou et al. 2013), pressurized liquid extraction (Vichapong et al. 2010), and solid-phase extraction (Tian et al. 2004; Chen et al. 2012a). For example, compared with solid-phase extraction, vortex-assisted extraction involves multistep sample extraction and cleanup procedures that use large amounts of solvents, which can result in compound losses. The high temperatures used in extractions with subcritical fluids can cause the degradation of phenolic acids or lead to their involvement in Maillard reactions. Furthermore, subcritical extracts contain carbohydrates, proteins, and amino acids, which can react with the Folin-Ciocalteu reagent during spectrophotometric analyses, which could produce erroneous results (Stratil et al. 2007).

The literature also reveals that the TPC might depend on the analytical method used: Na_2_CO_3_-based Folin-Ciocalteu assay (Chi et al. 2007; De Mira et al. 2009), ethanolamine-based Folin-Ciocalteu assay (Laokuldilok et al. 2011; Min et al. 2011), or Prussian blue assay (Finocchiaro et al. 2007). To the best of the authors' knowledge, no study has concentrated on comparing the three assays to optimize extraction and spectrophotometry determinations of phenolic acids in rice.

### Extraction and analysis of flavonoids

The same factors that affect the extraction of phenolic acids apply to flavonoids. The choice of reagents has also been found to be an important factor in the determination of the TFC using the aluminum chloride method. Three assays are currently available: the NaNO_2_-based aluminum chloride assay, the potassium acetate-based aluminum chloride assay, and the sodium borohydride/chloranil-based aluminum chloride assay. The sodium borohydride/chloranil method clearly yields values that are significantly higher than those of the two other assays (Zhang et al. 2010).

### Extraction and analysis of anthocyanins and proanthocyanidins

Different solvents have been evaluated for their effectiveness in extracting anthocyanins and proanthocyanidins from cereals. For anthocyanins, 85% methanol/1 mol/L HCl under cold conditions was found to be a suitable extraction solvent (Hiemori et al. 2009; Saikia et al. 2012), along with 85% methanol (Chen et al. 2012a; Gunaratne et al. 2013) or acetone:water:acetic acid (70:29.5:0.5, v/v) for free proanthocyanidins (Min et al. 2011). Although these solvent combinations are most commonly used, the extraction protocol (defatation or not, sonication or not, heating or not) can also play an important role in extracting anthocyanins and proanthocyanidins from rice as was highlighted above for phenolic acids. Different methods used for the determination of the TAC, that is, direct spectrophotometric assay (Hirawan et al. 2011; Sompong et al. 2011) and pH differential assay (Sam et al. 2008; Saikia et al. 2012), and the total proanthocyanidin content, that is, vanillin assay (Sangkitikomol et al. 2010; Gunaratne et al. 2013) and ammonium iron (II) sulfate assay (Finocchiaro et al. 2007), are considered additional factors responsible for the high variation observed in responses. To the best of the authors' knowledge, no systematic comparison has been attempted regarding the advantages and drawbacks of these various techniques.

### Extraction and analysis of tocopherols and tocotrienols

With regard to tocopherols and tocotrienols, hexane (Diack and Sask 1994; Xu et al. 2001), methanol (Miller and Engel 2006; Jeng et al. 2012), and acetone (Mandak and Nystrom 2012; Gunaratne et al. 2013) are most commonly used for their extraction from rice grains, usually at temperatures varying from 60 to 80°C. Under optimal extraction conditions, however, methanol could extract more vitamin E isomers than hexane and acetone (Imsanguan et al. 2008). Nevertheless, the extraction temperature appears to be the most important factor, as a certain temperature might cause the degradation of vitamin E. Direct solvent extraction and saponification-assisted extraction are used for the analysis of tocols in rice. Saponification is judged by some authors to be necessary to reduce the load of waxes and triglycerides in the extracts, which can interact with the adsorbents during HPLC analyses to various degrees and affect the separation and detection selectivity. The saponification process is accomplished using 50–80% KOH in the presence of antioxidants such as ascorbic acid (Xu et al. 2001; Moongngarm et al. 2012), pyrogallol (Zhang et al. 2012), and butylated hydroxytoluene (Sookwong et al. 2007; Lin and Lai 2011), which are expected to protect the tocols from oxidation. Diack and Sask (1994) found a 50% loss of vitamin E during saponification under inert gas without an antioxidant and a 16% loss with an antioxidant. Extractant tools such as supercritical fluids (Imsanguan et al. 2008), ultrasonication (Diack and Sask 1994; Moongngarm et al. 2012), soxhlation (Imsanguan et al. 2008; Mohanlal et al. 2012), and vortexing (Huang and Ng 2011; Gunaratne et al. 2013) have also been used to obtain vitamin E isomers from rice. When these methods were compared with respect to their effectiveness in extracting vitamin E from rice, supercritical fluids extraction ranked first, followed by soxhlet extraction and vortex-assisted extraction (Imsanguan et al. 2008).

Finally, the wide variation in the data reported for vitamin E isomers can be related to the diversity of the analytical methods currently available. In contrast to phenolic compounds, where only reverse phase-HPLC is used for the analytical resolution of the various compounds, both normal phase-HPLC and reverse phase-HPLC have been used for the separation of vitamin E isomers. When normal phase-HPLC is used, relatively easy separations of eight isomers is obtained (Sookwong et al. 2007; Goufo et al. 2014a). By contrast, reverse phase-HPLC using standard C8 and C18 microparticulate stationary phases usually fails to resolve the isomeric *β*-and *γ*-T and-T3 (Finocchiaro et al. 2007; Pascual et al. 2013). In such cases, most authors consider the (*β *+ *γ*) peak as *γ*, therefore omitting the presence of *β*-T.

### Extraction and analysis of γ-oryzanol

All of the methods described above for vitamin E extraction have also been used for the simultaneous extraction of *γ*-oryzanol and vitamin E from rice. Methods for the analysis of *γ*-oryzanol in rice comprise UV-spectrophotometry (Bucci et al., 2003), normal phase-HPLC (Huang and Ng 2011; Goufo et al. 2014a), reverse phase-HPLC (Lin and Lai 2011; Pascual et al. 2013), and gas chromatography (Miller and Engel 2006). The normal phase-HPLC method yields determination values that are significantly lower than those obtained from the UV-spectrophotometry method (Bucci et al., 2003). The difference between the two methods arises from the fact that all of the substances that have an absorbance maximum at 315 nm are computed along with *γ*-oryzanol during UV-spectrophotometric analysis, whereas normal phase-HPLC separates *γ*-oryzanol from the other compounds before determination. The 25 components of *γ*-oryzanol can be separated using reverse phase-HPLC or gas chromatography. With normal phase-HPLC, only one to three peaks are reported. A major problem one has to face in the quantification of individual steryl ferulate is the lack of commercially available pure standards. The purity of standards obtained through synthesis or by purification from natural sources in the authors' own laboratories could lead to a misinterpretation in the quantification of steryl ferulates in rice.

### Extraction and analysis of phytic acid

The phytic acid content of rice, as reported by different researchers, shows less variation than that of the other antioxidants described above, likely because phytic acid is classified as a carbohydrate. As stated above, primary metabolites show less variability compared with secondary metabolites. In addition, most methods used for the extraction of phytic acid from rice are based on a collaboratively tested method (Official Methods of the Association of Analytical Communities No. 986.11).

Parameters that potentially affect the quantification of phytic acid include the extraction solvent, the precipitation or purification scheme, and the analytical procedure. Three extraction media are generally used for the extraction of phytic acid from rice: 1.2% HCl/10% Na_2_SO_4_ (Moongngarm et al. 2012), 2.4% HCl/NaOH (Ren et al. 2007; Wang et al. 2011), and 2.4% HCl (Liang et al. 2007; Frontela et al. 2008). After extraction, phytic acid is either precipitated using a ferric chloride solution (FeCl_3_/NaOH/HCl) (Wei et al. 2007; Wang et al. 2011) or purified through an anion-exchange resin (Marfo et al. 1990). Quantification is carried out by colorimetry/titration (Moongngarm et al. 2012), digestion/colorimetry/spectrophotometry (Liang et al. 2007; Mohanlal et al. 2012), colorimetry/spectrophotometry (Wei et al. 2007; Wang et al. 2011), HPLC/refractive index detector (Frank et al. 2009), or high-performance ion chromatography (HPIC)/Dionex conductivity detector (Ren et al. 2007). Indirect measurements of phytic acid are based on the determination of phosphorus (Marfo et al. 1990; Mohanlal et al. 2012) or on the stoichiometric relationship between ferric ion and phytate for methods based on precipitation using ferric chloride (Wei et al. 2007; Wang et al. 2011). For the latter, marked discrepancies in the results have been observed, partly because the ratio of iron (III) ions that reacts with each molecule of phytic acid, up to a maximum of four ions, varies depending on the choice of reagents. On the other hand, methods based on quantification by titration and spectrophotometry appear to overestimate the phytic acid content in rice because they do not differentiate among inositol hexaphosphate (InsP6), pentaphosphate (InsP5), tetraphosphate (InsP4), triphosphate (InsP3), diphosphate (InsP2), or monophosphate (InsP1) as do HPLC methods. HPIC does not require a prepurification step, and is hence considered superior to HPLC for quantifying phytic acid.

## Current Needs and Future Directions

In the last few decades, there has been considerable interest in the chemistry of rice antioxidant compounds. From a qualitative and a quantitative point of view, however, the antioxidant composition of rice remains unresolved. For example, little is known about the identity of phenolic compounds. To resolve this, there is an urgent need to take advantage of the rapid development of analytical techniques such as diode array spectroscopy and mass spectrometry. Advances in microwave extraction and enzyme-assisted extraction techniques are promising, and these extraction procedures may also be applied for extracting antioxidant compounds from rice. Besides the compounds reviewed in this study, the antioxidant activities of several alkaloids (e.g., 4-carboethoxy-6-hydroxy-2-quinolone; Chung and Shin 2007), protein fractions (albumin, globulin, glutelin, and prolamin; Adebiyi et al. 2009; Zhang et al. 2009), and polysaccharide fractions (Zha et al. 2009) have been reported in rice. This is an area of research that deserves further attention. A key barrier to research in the area of rice antioxidants is the lack of validated methods, as described above. Therefore, establishing standardized methods for the extraction and analysis of rice antioxidants through collaborative studies involving international laboratories is warranted. However, rice antioxidants are naturally prone to variation, and the large genotypic differences in the contents found in this study may present new opportunities for breeding varieties with a higher ratio of all of these antioxidant compounds. Owing to legal restrictions and consumer concerns of the use of synthetic additives in food, interest in natural sources of antioxidants has intensified in recent years. The rice bran appears to be a good candidate in this respect, and may hold promise for the development of rice-based functional foods, pharmaceuticals, and cosmetic products. The data presented herein show that the rice husk contains a unique complex of naturally occurring antioxidant compounds; for example, tricin and isovitexin. However, further studies are needed to determine how to best incorporate the husk in value-added products. This review also paves the way for more in-depth investigations of the factors affecting the contents of antioxidant compounds in rice. For example, rice is classified with regard to its flavor into aromatic and nonaromatic species (Goufo et al. 2010, 2011). The comparison of the two types of rice varieties with respect to their contents of antioxidant compounds should be conducted. Finally, it should be noted that approximately 90% of the studies used to build the database presented in this review arose from the analysis of rice varieties grown in Asia. Whether the antioxidant profile of rice varieties grown in Europe, Africa, or South America is the same remains uncertain. This represents a promising area for future investigation. Moreover, estimating dietary intakes require quantitative knowledge on varieties native to each region and those vastly consumed by local populations.

## Supporting Information

Data S1 Rice antioxidants database.

Data S2 Methods used for the extraction and analysis of rice antioxidants.

Table S1 Effect of rice bran color on rice antioxidants.
