# Supplementary material for: Rice antioxidants: phenolic acids, flavonoids, anthocyanins, proanthocyanidins, tocopherols, tocotrienols, γ-oryzanol, and phytic acid
Source: Food Sci Nutr. 2014 Jan 21;2(2):75–104. doi: 10.1002/fsn3.86 (PMC3959956; doi:10.1002/fsn3.86)
Supplement: Table S1 — Effect of rice bran color on rice antioxidants. [file fsn30002-0075-sd3.doc]

**Supporting Information 2. Distribution of phenolic acids, anthocyanins, and proanthocyanidins in non-pigmented (brown) and pigmented (black, red, and purple) rice, and their antioxidant activities.**

| **Phenolic acids (mg/100 g)** | **Color** | **Rice endosperm** |  |  | **Rice bran** |  |  | | **Rice whole grain** |  | |  | **Rice husk** |  |  |
| --- | --- | --- | --- | --- | --- | --- | --- | --- | --- | --- | --- | --- | --- | --- | --- |
|  |  | **Soluble** | **Insoluble** | **Total** | **Soluble** | **Insoluble** | **Total** | | **Soluble** | **Insoluble** | | **Total** | **Soluble** | **Insoluble** | **Total** |
|  |  |  |  |  |  |  |  | |  |  | |  |  |  |  |
| Gallic acid | Brown | 0.02–0.19 (n = 4) | 0.32 (n = 1) |  | 0.05–0.42 (n = 5) | 0.21–2.09 (n = 2) |  | | 0.04–0.65 (n = 7) | 0.51–1.60 (n = 3) | |  | 0.04–0.06 (n = 2) | 0.15 (n = 1) |  |
|  | Brown | 0.08 ± 0.08 | 0.32 ± 0.00 | 0.40 ± 0.08 | 0.20 ± 0.15 | 1.15 ± 1.33 | 1.35 ± 1.48 | 0.21 ± 0.22 | 1.16 ± 0.58 | | 1.38 ± 0.79 | 0.05 ± 0.01 | 0.15 ± 0.00 | 0.20 ± 0.01 | |
|  | Black | ND (n = 2) |  |  | 1.20–8.30 (n = 4) | 1.88–4.06 (n = 3) |  | 2.03–6.44  (n = 3) |  | |  | ND (n = 1) |  |  | |
|  | Black | ND | NA | NA | 5.00 ± 2.98 | 2.65 ± 1.22 | 7.65 ± 4.20 | 3.57 ± 2.49 | NA | | NA | ND | NA | NA | |
|  | Red | ND (n = 1) |  |  | 1.18–2.21 (n = 3) | 1.69 (n = 1) |  | 0.23–0.52  (n = 3) |  | |  | ND (n = 1) |  |  | |
|  | Red | ND | NA | NA | 1.53 ± 0.59 | 1.69 ± 0.00 | 3.22 ± 0.59 | 0.42 ± 0.16 | NA | | NA | ND | NA | NA | |
|  | Purple |  |  |  |  |  |  | 2.88 (n = 1) |  | |  |  |  |  | |
|  | Purple | NA | NA | NA | NA | NA | NA | 2.88 ± 0.00 | NA | | NA | NA | NA | NA | |
|  | pigmented | ND (n = 3) |  |  | 1.18–8.30 (n = 7) | 1.69–4.06 (n = 4) |  | 0.23–6.44  (n = 6) |  | |  | ND (n = 1) |  |  | |
|  | pigmented | ND | NA | NA | 3.26 ± 1.78 | 2.17 ± 0.61 | 5.43 ± 2.40 | 2.00 ± 1.32 | NA | | NA | ND | NA | NA | |
|  |  |  |  |  |  |  |  |  |  | |  |  |  |  | |
| Protocatechuic acid | Brown | 0.01–0.10 (n = 4) | 0.17 (n = 1) |  | 0.33–1.07 (n = 4) | 0.04–0.72 (n = 3) |  | 0.04–0.24  (n = 4) | 0.17–1.38 (n = 2) | |  | 0.15–0.21 (n = 2) | 0.06 (n = 1) |  | |
|  | Brown | 0.06 ± 0.05 | 0.17 ± 0.00 | 0.23 ± 0.05 | 0.61 ± 0.34 | 0.34 ± 0.35 | 0.94 ± 0.69 | 0.12 ± 0.09 | 0.78 ± 0.86 | | 0.90 ± 0.95 | 0.18 ± 0.04 | 0.06 ± 0.00 | 0.24 ± 0.04 | |
|  | Black |  |  |  | 1.98–4.15 (n = 4) | 12.32–24.50  (n = 4) |  | 2.56–4.50  (n = 5) | 1.00–4.48 (n = 3) | |  |  |  |  | |
|  | Black | NA | NA | NA | 3.11 ± 0.90 | 18.27 ± 6.10 | 21.37 ± 7.00 | 3.35 ± 0.78 | 1.12 ± 0.12 | | 4.47 ± 0.89 | NA | NA | NA | |
|  | Red |  |  |  | 1.69–9.70 (n = 3) | 6.04–7.10 (n = 2) |  | 0.68–2.80  (n = 5) | 0.01–1.26 (n = 3) | |  |  |  |  | |
|  | Red | NA | NA | NA | 4.51 ± 4.50 | 6.57 ± 0.75 | 11.08 ± 5.25 | 1.64 ± 0.89 | 0.02 ± 0.01 | | 1.66 ± 0.90 | NA | NA | NA | |
|  | Purple |  |  |  | 1.54 (n = 1) | 4.23 (n = 1) |  |  |  | |  |  |  |  | |
|  | Purple | NA | NA | NA | 1.54 ± 0.00 | 4.23 ± 0.00 | 5.77 ± 0.00 | NA | NA | | NA | NA | NA | NA | |
|  | Pigmented |  |  |  | 1.98–9.70 (n = 7) | 6.04–24.50  (n = 5) |  | 0.68–4.50 (n = 10) | 0.01–1.23 (n = 6) | |  |  |  |  | |
|  | Pigmented | NA | NA | NA | 3.81 ± 2.70 | 12.42 ± 3.42 | 16.23 ± 6.12 | 2.50 ± 0.83 | 0.57 ± 0.06 | | 3.07 ± 0.89 | NA | NA | NA | |
|  |  |  |  |  |  |  |  |  |  | |  |  |  |  | |
| *p*–Hydroxybenzoic acid | Brown | 0.01–0.05  (n = 5) | ND (n = 0) |  | 0.25–1.39 (n = 5) | 0.06–6.73 (n = 5) |  | 0.02–0.25 (n = 9) | 0.2–0.74 (n = 4) | |  | 0.11–0.53 (n = 2) | 0.03–5.26 (n = 2) |  | |
|  | Brown | 0.02 ± 0.02 | ND | NA | 0.62 ± 0.53 | 3.08 ± 3.54 | 3.71 ± 4.06 | 0.10 ± 0.08 | 0.39 ± 0.27 | | 0.49 ± 0.35 | 0.32 ± 0.30 | 2.65 ± 3.70 | 2.97 | |
|  | Black |  |  |  | 4.44–26.43 (n = 7) | 27.71–52.52  (n = 3) |  | 0.10–5.99 (n = 11) | 0.19–1.19 (n–5) | |  | 0.78–1.96 (n = 3) | NA (n = 0) |  | |
|  | Black | NA | NA | NA | 16.72 ± 6.64 | 36.89 ± 13.61 | 53.60 ± 20.25 | 1.77 ± 1.82 | 0.73 ± 0.41 | | 2.50 ± 2.22 | 1.35 ± 0.59 | NA | NA | |
|  | Red |  |  |  | 1.25–8.21 (n = 5) | 4.00–4.27 (n = 2) |  | 0.10–4.69 (n = 11) | 0.29–1.25 (n = 6) | |  | 0.46 (n = 1) |  |  | |
|  | Red | ND | NA | NA | 4.68 ± 3.10 | 4.14 ± 0.19 | 8.82 ± 3.29 | 0.93 ± 1.38 | 0.83 ± 0.39 | | 1.77 ± 1.76 | 0.46 ± 0.00 | NA | NA | |
|  | Purple |  |  |  | 0.07 (n = 1) | 0.05 (n = 1) |  |  |  | |  |  |  |  | |
|  | Purple | NA | NA | NA | 0.07 ± 0.00 | 0.05 ± 0.00 | 0.12 ± 0.00 | NA | NA | | NA | NA | NA | NA | |
|  | Pigmented | ND (n = 1) |  |  | 1.25–26.43  (n = 12) | 4.00–52.52  (n = 5) |  | 0.10–5.99  (n = 22) | 0.19–1.25 (n = 11) | |  | 0.46–1.96 (n = 4) |  |  | |
|  | Pigmented | ND | NA | NA | 10.70 ± 4.87 | 20.51 ± 6.90 | 31.21 ± 11.77 | 1.35 ± 1.60 | 0.78 ± 0.40 | | 2.14 ± 2.00 | 0.91 ± 0.30 | NA | NA | |
|  |  |  |  |  |  |  |  |  |  | |  |  |  |  | |
| Vanillic acid | Brown | 0.02–0.15  (n = 6) | 0.03 (n = 1) |  | 0.28–1.64 (n = 7) | 0.02–0.34 (n = 3) |  | 0.04–0.40  (n = 13) | 0.10–1.10 (n = 5) | |  | 0.7–1.92 (n = 5) | 0.02–11.85  (n = 2) |  | |
|  | Brown | 0.05 ± 0.05 | 0.03 ± 0.00 | 0.08 ± 0.05 | 0.73 ± 0.51 | 0.13 ± 0.18 | 0.86 ± 0.69 | 0.19 ± 0.12 | 0.35 ± 0.42 | | 0.54 ± 0.54 | 0.94 ± 0.56 | 5.94 ± 8.37 | 6.88 ± 8.93 | |
|  | Black | ND (n = 1) |  |  | 1.09–15.40 (n = 4) |  |  | 1.37–4.40  (n = 8) | 1.81–17.70 (n = 6) | |  | 1.24–1.94 (n = 2) |  |  | |
|  | Black | ND | NA | NA | 11.71 ± 7.08 | NA | NA | 3.25 | 7.00 ± 6.15 | | 10.25 ± 7.19 | 1.59 ± 0.49 | NA | NA | |
|  | Red |  |  |  | 1.29–16.60 (n = 4) | 7.20 (n = 1) |  | 0.30–3.36  (n = 8) | 0.20–2.62 (n = 6) | |  | 0.45–0.81 (n = 3) |  |  | |
|  | Red | NA | NA | NA | 18.64 ± 20.27 | 55.25 ± 0.00 | 73.89 ± 20.27 | 1.46 ± 1.07 | 1.29 ± 0.96 | | 2.74 ± 2.03 | 0.63 ± 0.18 | NA | NA | |
|  | Purple |  |  |  | 53.50 (n = 1) | 103.30 (n = 1) |  |  |  | |  |  |  |  | |
|  | Purple | NA | NA | NA | 53.50 ± 0.00 | 103.30 ± 0.00 | 156.80 ± 0.00 | NA | NA | | NA | NA | NA | NA | |
|  | Pigmented | ND (n = 2) |  |  | 1.09–16.60 (n = 8) | 7.2 (n = 1) |  | 0.30–4.23 (n = 16) | 0.20–17.70 (n = 12) | |  | 0.45–1.94 (n = 5) |  |  | |
|  | Pigmented | ND | NA | NA | 15.17 ± 13.68 | 39.64 ± 0.00 | 54.81 ± 13.68 | 2.35 ± 1.05 | 4.15 ± 3.55 | | 6.50 ± 4.61 | 1.11 ± 0.34 | NA | NA | |
|  |  |  |  |  |  |  |  |  |  | |  |  |  |  | |
| Syringic acid | Brown | 0.01–0.19  (n = 5) | 0.01–0.05 (n = 2) |  | 0.07–0.58 (n = 4) | 0.08–0.21 (n = 2) |  | 0.02–0.49  (n = 11) | 0.14–1.04 (n = 5) | |  | 0.08–0.15 (n = 2) | 0.11–30.32  (n = 2) |  | |
|  | Brown | 0.05 ± 0.08 | 0.03 ± 0.03 | 0.08 ± 0.11 | 0.22 ± 0.24 | 0.15 ± 0.09 | 0.36 ± 0.34 | 0.17 ± 0.16 | 0.37 ± 0.38 | | 0.55 ± 0.53 | 0.12 ± 0.05 | 15.22 ± 21.36 | 15.33 ± 21.41 | |
|  | Black |  |  |  | 0.07 (n = 1) |  |  | 1.20–3.12  (n = 5) | 0.63–1.71 (n = 3) | |  |  |  |  | |
|  | Black | NA | NA | NA | 0.07 ± 0.00 | NA | NA | 1.82 ± 0.75 | 1.30 ± 0.38 | | 3.12 ± 1.12 | NA | NA | NA | |
|  | Red |  |  |  | 3.80–5.59 (n = 2) |  |  | 0.37–4.49  (n = 6) | 0.52–1.45 (n = 3) | |  |  |  |  | |
|  | Red | NA | NA | NA | 4.70 ± 1.27 | NA | NA | 1.98 ± 1.65 | 1.17 ± 0.31 | | 3.15 ± 1.95 | NA | NA | NA | |
|  | Purple |  |  |  | 0.42 (n = 1) |  |  |  |  | |  |  |  |  | |
|  | Purple | NA | NA | NA | 0.42 ± 0.00 | NA | NA | NA | NA | | NA | NA | NA | NA | |
|  | Pigmented |  |  |  | 0.07–5.59 (n = 3) |  |  | 0.37–4.49 (n = 11) | 0.84–1.71 (n = 6) | |  |  |  |  | |
|  | Pigmented | NA | NA | NA | 2.38 ± 0.63 | NA | NA | 1.90 ± 1.20 | 1.23 ± 0.34 | | 3.13 ± 1.54 | NA | NA | NA | |
|  |  |  |  |  |  |  |  |  |  | |  |  |  |  | |
| Chlorogenic acid | Brown | 0.02–0.06 (n = 3) | 0.04 (n = 1) |  | 0.11–1.55 (n = 5) | 0.21 (n = 1) |  | 0.02–0.44  (n = 7) | 0.04 (n = 1) | |  | 0.05–0.31 (n = 2) | 0.03 (n = 1) |  | |
|  | Brown | 0.04 ± 0.02 | 0.04 ± 0.00 | 0.08 ± 0.02 | 0.84 ± 0.28 | 0.21 ± 0.00 | 1.05 ± 0.28 | 0.14 ± 0.04 | 0.04 ± 0.00 | | 0.18 ± 0.04 | 0.18 ± 0.18 | 0.03 ± 0.00 | 0.21 ± 0.18 | |
|  | Black |  |  |  |  |  |  | 2.53 (n = 1) |  | |  |  |  |  | |
|  | Black | NA | NA | NA | NA | NA | NA | 2.53 ± 0.00 | NA | | NA | NA | NA | NA | |
|  | Red | ND (n = 1) |  |  | 9.79–15.40 (n = 3) |  |  | 1.60–3.98  (n = 2) |  | |  | 1.94 (n = 1) |  |  | |
|  | Red | ND | NA | NA | 12.51 ± 2.81 | NA | NA | 2.79 ± 1.68 | NA | | NA | 1.94 ± 0.00 | NA | NA | |
|  | Purple |  |  |  |  |  |  |  |  | |  |  |  |  | |
|  | Purple | NA | NA | NA | NA | NA | NA | NA | NA | | NA | NA | NA | NA | |
|  | Pigmented | ND (n = 1) |  |  | 9.79–15.4 (n = 3) |  |  | 1.6–3.98 (n = 2) |  | |  | 1.94 (n = 1) |  |  | |
|  | Pigmented | ND | NA | NA | 12.51 ± 2.81 | NA | NA | 2.79 ± 1.68 | NA | | NA | 1.94 ± 0.00 | NA | NA | |
|  |  |  |  |  |  |  |  |  |  | |  |  |  |  | |
| Caffeic acid | Brown | 0.02–0.15 (n = 4) | 0.02 (n = 1) |  | 0.18–0.79 (n = 7) | 0.06–0.15 (n = 3) |  | 0.11–0.51 (n = 9) | 0.22–0.61 (n = 3) | |  | 0.18–0.53 (n = 5) | 0.02 (n = 1) |  | |
|  | Brown | 0.06 ± 0.06 | 0.02 ± 0.00 | 0.08 ± 0.06 | 0.37 ± 0.21 | 0.09 ± 0.05 | 0.46 ± 0.26 | 0.25 ± 0.14 | 0.37 ± 0.21 | | 0.61 ± 0.35 | 0.30 ± 0.13 | 0.02 ± 0.00 | 0.32 ± 0.13 | |
|  | Black | ND (n = 1) |  |  | 19.19–25.28 (n = 3) |  |  | 0.09–1.06 (n = 4) | 0.02–0.05 (n = 3) | |  | 0.34–0.39 (n = 3) |  |  | |
|  | Black | ND | NA | NA | 22.29 ± 3.05 | NA | NA | 0.41 ± 0.44 | 0.04 ± 0.02 | | 0.45 ± 0.46 | 0.36 ± 0.03 | NA | NA | |
|  | Red | 0.07 (n = 1) |  |  | 2.78–6.54 (n = 3) | 11.10 (n = 1) |  | 0.13–1.06 (n = 5) | 0.01–0.03 (n = 3) | |  | 0.21–0.80 (n = 3) |  |  | |
|  | Red | 0.07 ± 0.00 | NA | NA | 6.71 ± 4.01 | 11.10 ± 0.00 | 17.81 ± 4.01 | 0.34 ± 0.41 | 0.02 ± 0.01 | | 0.36 ± 0.42 | 0.50 ± 0.30 | NA | NA | |
|  | Purple |  |  |  |  | 0.06 (n = 1) |  |  |  | |  |  |  |  | |
|  | Purple | NA | NA | NA | NA | 0.06 ± 0.00 | NA | NA | NA | | NA | NA | NA | NA | |
|  | Pigmented | 0.07 (n = 1) |  |  | 2.78–25.28 (n = 6) | 11.1 (n = 1) |  | 0.09–1.06 (n = 9) | 0.01–0.05 (n = 6) | |  | 0.21–0.39 (n = 6) |  |  | |
|  | Pigmented | 0.07 ± 0.00 | NA | NA | 14.50 ± 3.53 | 11.10 ± 0.00 | 25.60 ± 3.53 | 0.38 ± 0.42 | 0.03 ± 0.01 | | 0.40 ± 0.44 | 0.43 ± 0.16 | NA | NA | |
|  |  |  |  |  |  |  |  |  |  | |  |  |  |  | |
| *p*–Coumaric acid | Brown | 0.02–0.09  (n = 7) | 0.17–0.89 (n = 8) |  | 0.10–1.57  (n = 13) | 18.10–74.20  (n = 6) |  | 0.07–1.15  (n = 15) | 0.36–2.88  (n = 9) | |  | 1.14–2.07  (n = 5) | 78.66–591.7 (n = 2) |  | |
|  | Brown | 0.05 ± 0.02 | 0.51 ± 0.27 | 0.55 ± 0.30 | 0.87 ± 0.50 | 41.33 ± 20.42 | 42.20 ± 20.92 | 0.41 ± 0.32 | 1.21 ± 0.91 | | 1.62 ± 1.23 | 1.86 ± 0.55 | 335.1 ± 362.7 | 337.0 ± 363.3 | |
|  | Black | 0.96 (n = 1) | 0.54 (n = 1) |  | 1.76–6.20 (n = 8) | 13.92–144.00 (n = 4) |  | 0.30–4.11 (n = 11) | 2.00–6.70 (n = 7) | |  | 1.44–1.71 (n = 3) | ND (n = 1) |  | |
|  | Black | 0.96 ± 0.00 | 0.54 ± 0.00 | 1.50 ± 0.00 | 3.40 ± 1.65 | 48.85 ± 63.49 | 52.25 ± 65.15 | 1.35 ± 1.41 | 4.23 ± 1.25 | | 5.58 ± 2.62 | 1.60 ± 0.14 | ND | NA | |
|  | Red |  |  |  | 1.64–4.06 (n = 6) | 23.71–51.10 (n = 2) |  | 0.10–5.23 (n = 11) | 1.05–15.09 (n = 8) | |  |  |  |  | |
|  | Red | NA | NA | NA | 2.70 ± 0.94 | 37.41 ± 19.37 | 40.10 ± 20.31 | 1.62 ± 1.60 | 6.03 ± 4.71 | | 7.66 ± 6.31 | 1.88 ± 0.40 | NA | NA | |
|  | Purple |  |  |  | 0.47 (n = 1) | 51.70 (n = 1) |  |  |  | |  |  |  |  | |
|  | Purple | NA | NA | NA | 0.47 ± 0.00 | 51.70 ± 0.00 | 52.17 ± 0.00 | NA | NA | | NA | NA | NA | NA | |
|  | Pigmented | 0.96 (n = 1) | 0.54 (n = 1) |  | 1.64–6.20 (n = 14) | 16.77–144.00 (n = 6) |  | 0.30–5.23 (n = 22) | 1.25–15.05 (n = 15) | |  | 1.44–2.27 (n = 6) |  |  | |
|  | Pigmented | 0.96 ± 0.00 | 0.54 ± 0.00 | 1.50 ± 0.00 | 3.05 ± 1.30 | 43.13 ± 41.43 | 46.18 ± 42.73 | 1.49 ± 1.50 | 5.13 ± 2.98 | | 6.62 ± 4.48 | 1.74 ± 0.27 | NA | NA | |
|  |  |  |  |  |  |  |  |  |  | |  |  |  |  | |
| Sinapic acid | Brown | 0.01–0.05 (n = 3) | 0.06 (n = 1) |  | 0.04–0.60 (n = 4) | 0.20–3.47 (n = 5) |  | 0.11–0.35 (n = 4) | 1.42–2.28 (n = 2) | |  | 0.02–0.19 (n = 2) | 0.16 (n = 1) |  | |
|  | Brown | 0.03 ± 0.02 | 0.06 ± 0.00 | 0.09 ± 0.02 | 0.29 ± 0.26 | 1.77 ± 1.42 | 2.06 ± 1.68 | 0.23 ± 0.13 | 1.85 ± 0.65 | | 2.08 ± 0.73 | 0.11 ± 0.12 | 0.16 ± 0.00 | 0.27 ± 0.12 | |
|  | Black |  |  |  | 0.84–5.00 (n = 4) | 15.02–25.91 (n = 3) |  | ND (n = 2) | 5.51–7.33 (n = 3) | |  |  |  |  | |
|  | Black | NA | NA | NA | 2.99 ± 1.73 | 20.42 ± 5.45 | 23.41 ± 7.17 | ND | 6.39 ± 0.91 | | NA | NA | NA | NA | |
|  | Red | 1.92 (n = 1) |  |  | 0.65–2.64 (n = 2) | 18.34–22.60 (n = 2) |  | 4.72–16.07 (n = 2) | 5.68–9.69 (n = 3) | |  |  |  |  | |
|  | Red | 1.92 ± 0.00 | NA | NA | 1.65 ± 1.41 | 20.47 ± 3.01 | 22.12 ± 4.42 | 10.40 ± 8.03 | 7.74 ± 20.1 | | 18.14 ± 10.03 | NA | NA | NA | |
|  | Purple |  |  |  |  | 20.39 (n = 1) |  |  |  | |  |  |  |  | |
|  | Purple | NA | NA | NA | NA | 20.39 ± 0.00 | NA | NA | NA | | NA | NA | NA | NA | |
|  | Pigmented | 1.92 (n = 1) |  |  | 0.65–5.00 (n = 6) | 15.02–25.91 (n = 5) |  | 4.72–16.07 (n = 2) | 5.51–9.69 (n = 6) | |  |  |  |  | |
|  | Pigmented | 1.92 | NA | NA | 2.32 ± 1.57 | 20.44 ± 4.23 | 22.76 ± 5.80 | 10.40 ± 8.03 | 7.07 ± 1.46 | | 17.46 ± 9.49 | NA | NA | NA | |
|  |  |  |  |  |  |  |  |  |  | |  |  |  |  | |
| Ferulic acid | Brown | 0.37–2.78 (n = 10) | 0.99–9.35 (n = 9) |  | 1.37–8.69  (n = 13) | 11.99–225.00  (n = 9) |  | 0.26–3.24  (n = 16) | 3.90–25.90  (n = 8) | |  | 0.41–1.85  (n = 5) | 34.78–182.51 (n = 2) |  | |
|  | Brown | 1.04 ± 0.72 | 4.61 ± 2.76 | 5.65 ± 3.48 | 4.21 ± 2.38 | 116.16 ± 81.00 | 120.3 ± 83.38 | 1.14 ± 0.85 | 10.34 ± 7.32 | | 11.48 ± 8.17 | 1.26 ± 0.58 | 108.6 ± 104.4 | 109.9 ± 105.0 | |
|  | Black | 4.44 (n = 1) | 1.68 (n = 1) |  | 3.55–19.15 (n = 8) | 15.32–164.00 (n = 4) |  | 0.46–5.27 (n = 11) | 15.54–42.64 (n = 7) | |  | 0.43–0.93 (n = 3) |  |  | |
|  | Black | 4.44 ± 0.00 | 1.68 ± 0.00 | 6.12 ± 0.00 | 9.40 ± 6.10 | 81.10 ± 64.14 | 90.49 ± 70.24 | 2.29 ± 1.51 | 30.94 ± 9.14 | | 33.22 ± 10.66 | 0.69 ± 0.25 | NA | NA | |
|  | Red | 2.56 (n = 1) |  |  | 2.95–17.12 (n = 6) | 94.39–97.80 (n = 2) |  | 0.85–8.34 (n = 13) | 5.73–35.54 (n = 8) | |  | 0.61 (n = 1) |  |  | |
|  | Red | 2.56 ± 0.00 | NA | NA | 6.64 ± 5.40 | 96.10 ± 2.41 | 102.7 ± 7.81 | 2.55 ± 2.25 | 27.59 ± 6.83 | | 30.15 ± 9.07 | 0.61 ± 0.00 | NA | NA | |
|  | Purple |  |  |  | 7.00 (n = 1) | 152.30 (n = 1) |  |  |  | |  |  |  |  | |
|  | Purple | NA | NA | NA | 7.00 ± 0.00 | 152.3 ± 0.00 | 159.3 ± 0.00 | NA | NA | | NA | NA | NA | NA | |
|  | Pigmented | 2.56–4.44 (n = 2) | 1.68 (n = 1) |  | 2.95–19.15 (n = 14) | 15.32–94.85 (n = 6) |  | 0.46–8.34 (n = 24) | 14.03–39.5 (n = 15) | |  | 0.43–0.93 (n = 24) |  |  | |
|  | Pigmented | 3.50 ± 0.96 | 1.68 ± 0.00 | 5.48 ± 0.96 | 8.02 ± 5.75 | 88.60 ± 33.28 | 96.61 ± 39.03 | 2.42 ± 1.88 | 29.27 ± 7.98 | | 31.68 ± 9.87 | 0.65 ± 0.13 | NA | NA | |
|  |  |  |  |  |  |  |  |  |  | |  |  |  |  | |
| Cinnamic acid | Brown | 0.01 (n = 1) | 0.03 (n = 1) |  | 0.02 (n = 1) | 0.07 (n = 1) |  | 0.10–0.29 (n = 3) | 0.07 (n = 1) | |  | ND (n = 2) | ND (n = 1) |  | |
|  | Brown | 0.01 ± 0.00 | 0.03 ± 0.00 | 0.04 ± 0.00 | 0.02 ± 0.00 | 0.07 ± 0.00 | 0.09 ± 0.00 | 0.23 ± 0.11 | 0.07 ± 0.00 | | 0.30 ± 0.11 | ND | ND | ND | |
|  | Black |  |  |  |  |  |  |  |  | |  |  |  |  | |
|  | Black | NA | NA | NA | NA | NA | NA | NA | NA | | NA | NA | NA | NA | |
|  | Red |  |  |  |  |  |  | 0.29–3.84 (n = 4) |  | |  |  |  |  | |
|  | Red | NA | NA | NA | NA | NA | NA | 1.86 ± 1.57 | NA | | NA | NA | NA | NA | |
|  | Purple |  |  |  |  |  |  |  |  | |  |  |  |  | |
|  | Purple | NA | NA | NA | NA | NA | NA | NA | NA | | NA | NA | NA | NA | |
|  | Pigmented |  |  |  |  |  |  | 0.29–3.84 (n = 4) |  | |  |  |  |  | |
|  | Pigmented | NA | NA | NA | NA | NA | NA | 1.86 ± 1.57 | NA | | NA | NA | NA | NA | |
|  |  |  |  |  |  |  |  |  |  | |  |  |  |  | |
| Ellagic acid | Brown |  |  |  | 4.23 (n = 1) |  |  | 0.55 (n = 1) |  | |  | 4.34 (n = 1) |  |  | |
|  | Brown | NA | NA | NA | 4.23 ± 0.00 | NA | NA | 0.55 ± 0.00 | NA | | NA | 4.34 ± 0.00 | NA | NA | |
|  | Black |  |  |  | 12.21 (n = 1) |  |  | 1.26 (n = 1) |  | |  | 3.54 (n = 1) |  |  | |
|  | Black | NA | NA | NA | 12.21 ± 0.00 | NA | NA | 1.26 ± 0.00 | NA | | NA | 3.54 ± 0.00 | NA | NA | |
|  | Red |  |  |  | 5.51 (n = 1) |  |  | 0.49 (n = 1) |  | |  | 3.43 (n = 1) |  |  | |
|  | Red | NA | NA | NA | 5.51 ± 0.00 | NA | NA | 0.49 ± 0.00 | NA | | NA | 3.43 ± 0.00 | NA | NA | |
|  | Purple |  |  |  |  |  |  |  |  | |  |  |  |  | |
|  | Purple | NA | NA | NA | NA | NA | NA | NA | NA | | NA | NA | NA | NA | |
|  | Pigmented | ND (n = 2) |  |  | 5.51–12.21 (n = 2) |  |  | 0.49–1.26 (n = 2) |  | |  | 3.43–3.54 (n = 2) |  |  | |
|  | Pigmented | ND | NA | NA | 6.11 ± 8.63 | NA | NA | 0.63 ± 0.89 | NA | | NA | 1.77 ± 2.50 | NA | NA | |
|  |  |  |  |  |  |  |  |  |  | |  |  |  |  | |
|  |  |  |  |  |  |  |  |  |  | |  |  |  |  | |
|  |  |  |  |  |  |  |  |  |  | |  |  |  |  | |
| Total phenolic content | Brown | 3.00–50.60 (n = 26) | 10.00–84.00 (n = 3) |  | 129.0–980.0  (n = 64) | 191.12–545.0  (n = 5) |  | 24.00–252.4  (n = 69) | 12.30–200.0  (n = 16) | |  | 61–22–0.4  (n = 5) | 211.3–710.1 (n = 2) |  | |
|  | Brown | 18.04 ± 12.03 | 38.91 ± 39.56 | 56.95 ± 51.60 | 294.1 ± 162.6 | 302.2 ± 145.8 | 596.3 ± 308.5 | 102.6 ± 68.67 | 61.30 ± 52.23 | | 163.9 ± 120.9 | 138.4 ± 71.59 | 460.7 ± 352.6 | 599.2 ± 424.2 | |
|  | Black | 5.40–40.03 (n = 7) | 45.00–79.00 (n = 3) |  | 277.2–9850  (n = 21) | 227.0–8400  (n = 5) |  | 100.1–1640  (n = 42) | 77.00–401.6  (n = 11) | |  | 117.0–145.0  (n = 3) |  |  | |
|  | Black | 23.61 ± 11.65 | 56.36 ± 19.61 | 79.96 ± 31.26 | 1939 ± 2476 | 2032 ± 3569 | 3971 ± 6046 | 520.1 ± 409.9 | 166.3 ± 104.5 | | 686.4 ± 514.4 | 128.3 ± 14.74 | NA | NA | |
|  | Red | 10.00–52.32 (n = 8) | 29.35  (n = 1) |  | 439.0–7400  (n = 28) | 785.0  (n = 1) |  | 102.0–862.6  (n = 52) | 37.62–665.1  (n = 16) | |  | 117.0–131.0  (n = 3) |  |  | |
|  | Red | 28.02 ± 13.88 | 29.35 ± 0.00 | 57.37 ± 13.88 | 2600 ± 1619 | 785.0 ± 0.00 | 3385 ± 1619 | 347.7 ± 21101 | 169.9 ± 187.5 | | 517.6 ± 398.5 | 123.3 ± 7.09 | NA | NA | |
|  | Purple | 14.08  (n = 1) |  |  | 406.0–5121.0  (n = 9) | 819.0  (n = 1) |  | 110.0–547.3  (n = 12) | 40.00–95.07  (n = 2) | |  |  |  |  | |
|  | Purple | 14.08 ± 0.00 | NA | NA | 2353 ± 1657 | 819.0 ± 0.00 | 3172 ± 1657 | 229.3 ± 143.7 | 67.54 ± 38.94 | | 296.8 ± 182.7 | NA | NA | NA | |
|  | Pigmented | 5.40–52.32 (n = 16) | 29.35–79.00 (n = 4) |  | 277.2–9850  (n = 58) | 227.0–8400  (n = 7) |  | 100.1–1640  (n = 106) | 40.00–665.1  (n = 29) | |  | 117.0–145.00  (n = 6) |  |  | |
|  | Pigmented | 21.90 ± 8.51 | 42.85 ± 9.80 | 64.76 ± 18.32 | 2297 ± 1917 | 1212 ± 1189 | 3509 ± 3107 | 365.7 ± 254.9 | 134.5 ± 110.3 | | 500.3 ± 365.2 | 125.8 ± 10.92 | NA | NA | |
| **Anthocyanins (mg/100 g)** | **Color** | **Rice endosperm** |  |  | **Rice bran** |  |  | **Rice whole grain** |  | |  | **Rice husk** |  |  | |
|  |  | **Soluble** | **Insoluble** | **Total** | **Soluble** | **Insoluble** | **Total** | **Soluble** | **Insoluble** | | **Total** | **Soluble** | **Insoluble** | **Total** | |
|  |  |  |  |  |  |  |  |  |  | |  |  |  |  | |
| Peonidin 3–O–glucoside | Brown |  |  |  | 0.04–2.61 (n = 3) |  |  |  |  | |  |  |  |  | |
|  | Brown | NA | NA | NA | 0.96 ± 1.43 | NA | NA | NA | NA | | NA | NA | NA | NA | |
|  | Black |  |  |  | 14.17–534.0 (n = 6) |  |  | 2.30–162.1 (n = 13) |  | |  |  |  |  | |
|  | Black | NA | NA | NA | 200.1 ± 210.5 | NA | NA | 46.30 ± 56.85 | NA | | NA | NA | NA | NA | |
|  | Red |  |  |  | 66.76 (n = 0) |  |  | 25.65–100.1 (n = 2) |  | |  |  |  |  | |
|  | Red | NA | NA | NA | 66.76 ± 0.00 | NA | NA | 62.88 ± 52.65 | NA | | NA | NA | NA | NA | |
|  | Purple |  |  |  | 11.46–290.0 (n = 7) |  |  |  |  | |  |  |  |  | |
|  | Purple | NA | NA | NA | 104.8 ± 97.34 | NA | NA | NA | NA | | NA | NA | NA | NA | |
|  | Pigmented |  |  |  | 11.46–534.1  (n = 14) |  |  | 2.97–162.10 (n = 15) |  | |  |  |  |  | |
|  | Pigmented | NA | NA | NA | 123.9 ± 102.6 | NA | NA | 54.59 ± 54.75 | NA | | NA | NA | NA | NA | |
|  |  |  |  |  |  |  |  |  |  | |  |  |  |  | |
| Cyanidin 3–O–glucoside | Brown |  |  |  | 7.36 (n = 1) |  |  |  |  | |  |  |  |  | |
|  | Brown | NA | NA | NA | 7.36 ± 0.00 | NA | NA | NA | NA | | NA | NA | NA | NA | |
|  | Black |  |  |  | 150.0–2557  (n = 6) |  |  | 55.95–784.3 (n = 15) |  | |  |  |  |  | |
|  | Black | NA | NA | NA | 1071 ± 1029 | NA | NA | 274.4 ± 212.2 | NA | | NA | NA | NA | NA | |
|  | Red |  |  |  | 9.10–51.00 (n = 6) |  |  | 0.36–1.40 (n = 2) |  | |  |  |  |  | |
|  | Red | NA | NA | NA | 22.47 ± 15.79 | NA | NA | 0.88 ± 0.74 | NA | | NA | NA | NA | NA | |
|  | Purple |  |  |  | 130.0–2640 (n = 7) |  |  | 4.26–5.72 (n = 2) |  | |  |  |  |  | |
|  | Purple | NA | NA | NA | 1007 ± 853.4 | NA | NA | 4.99 ± 1.03 | NA | | NA | NA | NA | NA | |
|  | Pigmented |  |  |  | 9.10–2640  (n = 13) |  |  | 0.88–784.3 (n = 15) |  | |  |  |  |  | |
|  | Pigmented | NA | NA | NA | 700.2 ± 632.7 | NA | NA | 93.42 ± 71.32 | NA | | NA | NA | NA | NA | |
|  |  |  |  |  |  |  |  |  |  | |  |  |  |  | |
| Cyanidin 3–O–galactoside | Brown |  |  |  |  |  |  |  |  | |  |  |  |  | |
|  | Brown | NA | NA | NA | NA | NA | NA | NA | NA | | NA | NA | NA | NA | |
|  | Black |  |  |  |  |  |  |  |  | |  |  |  |  | |
|  | Black | NA | NA | NA | NA | NA | NA | NA | NA | | NA | NA | NA | NA | |
|  | Red |  |  |  |  |  |  |  |  | |  |  |  |  | |
|  | Red | NA | NA | NA | NA | NA | NA | NA | NA | | NA | NA | NA | NA | |
|  | Purple |  |  |  | 2.93–50.00 (n = 4) |  |  |  |  | |  |  |  |  | |
|  | Purple | NA | NA | NA | 28.29 ± 19.78 | NA | NA | NA | NA | | NA | NA | NA | NA | |
|  | Pigmented | NA (n = 0) | NA (n = 0) |  | 2.93–50.00 (n = 4) |  |  |  |  | |  |  |  |  | |
|  | Pigmented | NA | NA | NA | 28.29 ± 19.78 | NA | NA | NA | NA | | NA | NA | NA | NA | |
|  |  |  |  |  |  |  |  |  |  | |  |  |  |  | |
| Cyanidin 3–O–rutinoside | Brown |  |  |  | 6.19 (n = 1) |  |  |  |  | |  |  |  |  | |
|  | Brown | NA | NA | NA | 6.19 ± 0.00 | NA | NA | NA | NA | | NA | NA | NA | NA | |
|  | Black |  |  |  | 73.93–96.62 (n = 3) |  |  | 19.90 (n = 1) |  | |  |  |  |  | |
|  | Black | NA | NA | NA | 84.85 ± 11.37 | NA | NA | 19.90 ± 0.00 | NA | | NA | NA | NA | NA | |
|  | Red |  |  |  |  |  |  |  |  | |  |  |  |  | |
|  | Red | NA | NA | NA | NA | NA | NA | NA | NA | | NA | NA | NA | NA | |
|  | Purple |  |  |  | 3.17–47.32 (n = 3) |  |  | 13.78 (n = 1) |  | |  |  |  |  | |
|  | Purple | NA | NA | NA | 26.16 ± 22.13 | NA | NA | 13.78 ± 0.00 | NA | | NA | NA | NA | NA | |
|  | Pigmented |  |  |  | 3.17–96.62 (n = 6) |  |  | 13.78–19.90 (n = 2) |  | |  |  |  |  | |
|  | Pigmented | NA | NA | NA | 55.51 ± 16.75 | NA | NA | 16.84 ± 0.00 | NA | | NA | NA | NA | NA | |
|  |  |  |  |  |  |  |  |  |  | |  |  |  |  | |
| Catechin | Brown | ND (n = 2) |  |  | 0.55–2.35 (n = 4) |  |  | 0.26–0.44 (n = 4) |  | |  | 1.23–1.90 (n = 3) |  |  | |
|  | Brown | ND | NA | NA | 1.38 ± 0.74 | NA | NA | 0.34 ± 0.07 | NA | | NA | 1.54 ± 0.34 | NA | NA | |
|  | Black |  |  |  | 5.83–9.44 (n = 3) |  |  | 0.49–1.70 (n = 3) |  | |  | 1.79 (n = 1) |  |  | |
|  | Black | ND | NA | NA | 7.09 ± 2.04 | NA | NA | 1.19 ± 0.63 | NA | | NA | 1.79 ± 0.00 | NA | NA | |
|  | Red |  |  |  | 21.90–48.72 (n = 4) |  |  | 0.40–3.98 (n = 5) |  | |  | 1.57–3.16 (n = 3) |  |  | |
|  | Red | NA | NA | NA | 34.72 ± 11.67 | NA | NA | 1.44 ± 1.44 | NA | | NA | 2.24 ± 0.82 | NA | NA | |
|  | Purple |  |  |  |  |  |  |  |  | |  |  |  |  | |
|  | Purple | NA | NA | NA | NA | NA | NA | NA | NA | | NA | NA | NA | NA | |
|  | Pigmented |  |  |  | 5.83–48.72 (n = 7) |  |  | 0.40–3.98 (n = 8) |  | |  | 1.57–3.16 (n = 4) |  |  | |
|  | Pigmented | NA | NA | NA | 20.90 ± 6.85 | NA | NA | 1.32 ± 1.03 | NA | | NA | 2.02 ± 0.41 | NA | NA | |
|  |  |  |  |  |  |  |  |  |  | |  |  |  |  | |
| Epicatechin | Brown | ND (n = 1) |  |  |  |  |  | 0.34 (n = 1) |  | |  | 3.12–7.49 (n = 3) |  |  | |
|  | Brown | ND | NA | NA | NA | NA | NA | 0.34 ± 0.00 | NA | | NA | 4.98 ± 2.26 | NA | NA | |
|  | Black |  |  |  |  |  |  | 1.41 (n = 1) |  | |  | 3.80–6.14 (n = 3) |  |  | |
|  | Black | ND | NA | NA | NA | NA | NA | 1.41 ± 0.00 | NA | | NA | 4.98 ± 1.17 | NA | NA | |
|  | Red |  |  |  | 46.53 (n = 1) |  |  | 0.42 (n = 1) |  | |  | 0.44–0.57 (n = 3) |  |  | |
|  | Red | ND | NA | NA | 46.53 ± 0.00 | NA | NA | 0.42 ± 0.00 | NA | | NA | 0.50 ± 0.07 | NA | NA | |
|  | Purple |  |  |  |  |  |  |  |  | |  |  |  |  | |
|  | Purple | NA | NA | NA | NA | NA | NA | NA | NA | | NA | NA | NA | NA | |
|  | Pigmented |  |  |  | 46.53 (n = 1) |  |  | 0.42–1.41 (n = 2) |  | |  | 0.57–6.14 (n = 6) |  |  | |
|  | Pigmented | NA | NA | NA | 46.53 ± 0.00 | NA | NA | 0.92 ± 0.000 | NA | | NA | 2.74 ± 0.62 | NA | NA | |
|  |  |  |  |  |  |  |  |  |  | |  |  |  |  | |
| Total anthocyanins | Brown |  |  |  | 3.09 (n = 1) | 0.41 (n = 1) |  | 2.00–3.26 (n = 3) |  | |  |  |  |  | |
|  | Brown | NA | NA | NA | 3.09 ± 0.00 | 0.41 ± 0.00 | 3.50 ± 0.00 | 2.75 ± 0.67 | NA | | NA | NA | NA | NA | |
|  | Black | 17.51 (n = 1) |  |  | 113.5–5096 (n = 9) | 4.86–8.20 (n = 3) |  | 19.79–256.5 (n = 16) |  | |  |  |  |  | |
|  | Black | 17.51 ± 0.00 | NA | NA | 1884 ± 1794 | 6.66 ± 1.69 | 1891.40 | 138.6 ± 18.92 | NA | | NA | NA | NA | NA | |
|  | Red | 1.5 (N = 1) |  |  | 2.78–26.45 (n = 8) | 5.57 (n = 1) |  | 0.26–11.13 (n = 10) |  | |  |  |  |  | |
|  | Red | 1.50 ± 0.00 | NA | NA | 8.78 ± 8.84 | 5.57 ± 0.00 | 14.35 ± 8.84 | 4.07 ± 4.46 | NA | | NA | NA | NA | NA | |
|  | Purple |  |  |  | 155.0–6353 (n = 5) |  |  | 35.64 (n = 1) |  | |  |  |  |  | |
|  | Purple | NA | NA | NA | 2874 ± 2511 | NA | NA | 35.64 ± 0.00 | NA | | NA | NA | NA | NA | |
|  | Pigmented | 17.51 (n = 1) |  |  | 2.78–6353 (n = 22) | 4.86–8.20 (n = 4) |  | 4.07–256.5 (n = 16) |  | |  |  |  |  | |
|  | Pigmented | 17.51 ± 0.00 | NA | NA | 1589 ± 1438 | 6.12 ± 1.69 | 1595 ± 1440 | 59.43 ± 7.79 | NA | | NA | NA | NA | NA | |
|  |  |  |  |  |  |  |  |  |  | |  |  |  |  | |
| Total protoanthocyanidins | Brown |  |  |  | 2.24–6.44 (n = 2) |  |  | 5.02 (n = 1) |  | |  |  |  |  | |
|  | Brown | NA | NA | NA | 4.34 ± 2.97 | NA | NA | 5.02 ± 0.00 | NA | | NA | NA | NA | NA | |
|  | Black |  |  |  | 8.82–218.97 (n = 7) |  |  |  |  | |  |  |  |  | |
|  | Black | NA | NA | NA | 78.05 ± 79.39 | NA |  | NA | NA | | NA | NA | NA | NA | |
|  | Red |  |  |  | 80.00–2261 (n = 9) | ND (n = 1) |  | 5.04–202.0 (n = 7) |  | |  |  |  |  | |
|  | Red | NA | NA | NA | 716.6 ± 802.9 | ND | NA | 87.31 ± 77.44 | NA | | NA | NA | NA | NA | |
|  | Purple |  |  |  | 36.00–1260 (n = 5) |  |  | 22.40 (n = 1) |  | |  |  |  |  | |
|  | Purple | NA | NA | NA | 525.4 ± 506.4 | NA | NA | 22.40 ± 0.00 | NA | | NA | NA | NA | NA | |
|  | Pigmented |  |  |  | 8.82–2261 (n = 9) | ND (n = 1) |  | 5.04–202.1 (n = 7) |  | |  |  |  |  | |
|  | Pigmented | NA | NA | NA | 440.0 ± 462.9 | ND | NA | 54.85 ± 38.70 | NA | | NA | NA | NA | NA | |
|  |  |  |  |  |  |  |  |  |  | |  |  |  |  | |
| **Antioxidant Capacity** | **Color** | **Rice endosperm** |  |  | **Rice bran** |  |  | **Rice whole grain** |  | |  | **Rice husk** |  |  | |
|  |  | **Soluble** | **Insoluble** | **Total** | **Soluble** | **Insoluble** | **Total** | **Soluble** | **Insoluble** | | **Total** | **Soluble** | **Insoluble** | **Total** | |
| DPPH (mM trolox Eq/100 g) | Brown |  |  |  | 0.48–1.31  (n = 7) |  |  | 0.04–0.97  (n = 5) | 0.35–2.42  (n = 1) | |  |  |  |  | |
|  | Brown | NA | NA | NA | 0.87 ± 0.33 | NA | NA | 0.28 ± 0.39 | 2.42 ± 0.00 | | 2.70 ± 0.39 | NA | NA | NA | |
|  | Black |  |  |  |  |  |  | 0.38–0.79 (n = 10) | 0.25–0.44  (n = 3) | |  | 0.40  (n = 1) |  |  | |
|  | Black | NA | NA | NA | NA | NA | NA | 0.63 ± 0.15 | 0.42 ± 0.03 | | 1.04 ± 0.18 | 0.40 ± 0.00 | NA | NA | |
|  | Red |  |  |  | 5.14–51.98 (n = 10) |  |  | 0.41–0.91  (n = 9) | 0.11–0.69  (n = 3) | |  |  |  |  | |
|  | Red | NA | NA | NA | 17.90 ± 13.95 | NA | NA | 0.68 ± 0.17 | 0.55 ± 0.14 | | 1.23 ± 0.31 | NA | NA | NA | |
|  | Purple |  |  |  | 5.63–94.04  (n = 7) |  |  |  |  | |  |  |  |  | |
|  | Purple | NA | NA | NA | 38.74 ± 29.67 | NA | NA | NA | NA | | NA | NA | NA | NA | |
|  | Pigmented |  |  |  | 5.14–94.04 (n = 17) |  |  | 0.38–0.91 (n = 19) | 0.39–0.69  (n = 6) | |  | 0.40  (n = 1) |  |  | |
|  | Pigmented | NA | NA | NA | 28.32 ± 21.81 | NA | NA | 0.65 ± 0.16 | 0.49 ± 0.12 | | 1.14 ± 0.24 | 0.40 ± 0.00 | NA | NA | |
|  |  |  |  |  |  |  |  |  |  | |  |  |  |  | |
| DPPH (mg trolox Eq/100 g**)** | Brown | 29.11–378.1 (n = 3) | 17.26.–108.4 (n = 3) |  | 133.9–510.1 (n = 7) | 122.4–484.3 (n = 2) |  | 66.17–750.0 (n = 4) | 23.44–400.0 (n = 3) | |  | 107.8–178.5  (n = 3) | 91.32–147.6  (n = 3) |  | |
|  | Brown | 245.0 ± 188.1 | 26.45 ± 0.00 | 271.4 ± 188.1 | 378.8 ± 130.7 | 303.3 ± 255.9 | 682.2 ± 386.6 | 394.6 ± 273.9 | 199.4 ± 176.56 | | 594.0 ± 450.46 | 160.5 ± 0.00 | 117.1 ± 0.00 | 277.6 ± 0.00 | |
|  | Black | 641.0  (n = 1) |  |  |  |  |  | 6257  (n = 1) |  | |  |  |  |  | |
|  | Black | 641.0 ± 0.00 | NA | NA | NA | NA | NA | 6257 ± 0.00 | NA | | NA | NA | NA | NA | |
|  | Red | 695.0  (n = 1) |  |  | 10753  (n = 1) | 697.0  (n = 1) |  | 4195–6004 (n = 2) | 335.0  (n = 1) | |  | 120.0  (n = 1) |  |  | |
|  | Red | 695.0 ± 0.00 | NA | NA | 10753 ± 0.00 | 697.0 ± 0.00 | 11450 ± 0.00 | 5099 ± 1279 | 335.0 ± 0.00 | | 5434 ± 1279 | 120.0 ± 0.00 | NA | NA | |
|  | Purple |  |  |  | 7643.0  (n = 1) | 632.0  (n = 1) |  | 3157  (n = 1) | 437.0  (n = 1) | |  |  |  |  | |
|  | Purple | NA | NA | NA | 7643 ± 0.00 | 632 ± 0.00 | 8275 ± 0.00 | 3157 ± 0.00 | 437.0 ± 0.00 | | 3594 ± 0.00 | NA | NA | NA | |
|  | Pigmented | 641.0–695.0 (n = 2) |  |  | 7643.0–10753 (n = 2) | 632.0–697.0 (n = 2) |  | 3157–6257 (n = 4) | 335.0–437.0 (n = 2) | |  | 120.0  (n = 1) |  |  | |
|  | Pigmented | 668.0 ± 0.00 | NA | NA | 9198 ± 0.00 | 664.5 ± 0.00 | 9862 ± 0.00 | 4837 ± 426.3 | 386 ± 0.00 | | 5223 ± 426.3 | 120.0 ± 0.00 | NA | NA | |
|  |  |  |  |  |  |  |  |  |  | |  |  |  |  | |
| DPPH (%) | Brown | 5.43–61.40 (n = 11) | 6.92–29.54 (n = 2) |  | 18.30–93.91 (n = 15) | 29.08–98.68 (n = 2) |  | 15.86–85.50 (n = 18) | 16.58–54.03 (n = 2) | |  | 37.82–89.55 (n = 4) | 27.85–90.61 (n = 2) |  | |
|  | Brown | 35.13 ± 20.89 | 18.23 ± 15.99 |  | 55.67 ± 29.72 | 63.88 ± 49.21 |  | 50.86 ± 21.54 | 35.31 ± 26.48 | |  | 55.33 ± 23.53 | 59.23 ± 44.38 |  | |
|  | Black | 3.10  (n = 1) |  |  | 43.20–92.50 (n = 2) |  |  | 55.02–96.10 (n = 12) |  | |  |  |  |  | |
|  | Black | 3.10 ± 0.00 | NA |  | 67.85 ± 34.86 | NA |  | 75.58 ± 13.16 | NA | |  | NA | NA |  | |
|  | Red | 6.00  (n = 1) |  |  | 72.90–94.10 (n = 2) |  |  | 37.24–96.70 (n = 14) |  | |  |  |  |  | |
|  | Red | 6.00 ± 0.00 | NA |  | 83.97 ± 10.63 | NA |  | 66.99 ± 20.04 | NA | |  | NA | NA |  | |
|  | Purple |  |  |  |  |  |  |  |  | |  |  |  |  | |
|  | Purple | NA | NA |  | NA | NA |  | NA | NA | |  | NA | NA |  | |
|  | Pigmented | 3.10–6.00  (n = 2) |  |  | 43.20–94.10 (n = 4) |  |  | 37.24–96.70 (n = 26) |  | |  |  |  |  | |
|  | Pigmented | 4.55 ± 0.00 | NA |  | 75.91 ± 22.75 | NA |  | 71.28 ± 16.60 | NA | |  | NA | NA |  | |
|  |  |  |  |  |  |  |  |  |  | |  |  |  |  | |
| DPPH (EC50, mg/mL) | Brown | 19.00–26.41 (n = 5) |  |  | 0.15–35.43 (n = 24) |  |  | 2.75–72.18 (n = 5) |  | |  |  |  |  | |
|  | Brown | 15.06 ± 7.87 | NA | NA | 19.49 ± 15.33 | NA | NA | 18.45 ± 30.08 | NA | | NA | NA | NA | NA | |
|  | Black |  |  |  | 0.02–4.57  (n = 9) |  |  | 0.05  (n = 1) |  | |  |  |  |  | |
|  | Black | NA | NA | NA | 0.72 ± 1.49 | NA | NA | 0.05 ± 0.00 | NA | | NA | NA | NA | NA | |
|  | Red |  |  |  | 0.11–5.12  (n = 5) |  |  | 0.42  (n = 1) |  | |  |  |  |  | |
|  | Red | NA | NA | NA | 1.31 ± 2.15 | NA | NA | 0.42 ± 0.00 | NA | | NA | NA | NA | NA | |
|  | Purple |  |  |  | 1.50  (n = 1) |  |  | 0.01–0.05  (n = 4) |  | |  |  |  |  | |
|  | Purple | NA | NA | NA | 1.50 ± 0.00 | NA | NA | 0.03 ± 0.02 | NA | | NA | NA | NA | NA | |
|  | Pigmented |  |  |  | 0.02–5.12 (n = 15) |  |  | 0.01–59.30 (n = 6) |  | |  |  |  |  | |
|  | Pigmented | NA | NA | NA | 1.17 ± 1.21 | NA | NA | 19.92 ± 0.01 | NA | | NA | NA | NA | NA | |
|  |  |  |  |  |  |  |  |  |  | |  |  |  |  | |
| Inhibition of lipid peroxidation (linoleic acid, %) | Brown | 11.32–79.82 (n = 8) |  |  | 35.62–91.58 (n = 8) |  |  | 8.96–68.01 (n = 11) |  | |  | 17.26  (n = 1) |  |  | |
|  | Brown | 32.74 ± 20.77 | NA | NA | 56.43 ± 22.37 | NA | NA | 37.77 ± 23.96 | NA | | NA | 17.26 ± 0.00 | NA | NA | |
|  | Black |  |  |  | 32.56–80.86 (n = 4) |  |  | 60.00  (n = 1) |  | |  |  |  |  | |
|  | Black | NA | NA | NA | 53.47 ± 21.67 | NA | NA | 60.00 ± 0.00 | NA | | NA | NA | NA | NA | |
|  | Red |  |  |  | 40.00–64.32 (n = 3) |  |  |  |  | |  |  |  |  | |
|  | Red | NA | NA | NA | 53.17 ± 12.29 | NA | NA | NA | NA | | NA | NA | NA | NA | |
|  | Purple |  |  |  |  |  |  |  |  | |  |  |  |  | |
|  | Purple | NA | NA | NA | NA | NA | NA | NA | NA | | NA | NA | NA | NA | |
|  | Pigmented |  |  |  | 32.56–80.86 (n = 7) |  |  | 60.00  (n = 1) |  | |  |  |  |  | |
|  | Pigmented | NA | NA | NA | 53.32 ± 16.98 | NA | NA | 60.00 ± 0.00 | NA | | NA | NA | NA | NA | |
|  |  |  |  |  |  |  |  |  |  | |  |  |  |  | |
| ORAC (mM trolox Eq/100 g) | Brown | 0.85  (n = 1) |  |  | 7.02–101.70 (n = 4) | 44.97  (n = 1) |  | 0.02–4.33  (n = 8) | 0.01–0.04  (n = 3) | |  |  |  |  | |
|  | Brown | 0.85 ± 0.00 | NA | NA | 47.02 ± 47.70 | 44.97 ± 0.00 | 91.99 ± 47.70 | 1.79 ± 0.70 | 0.12 ± 0.16 | | 1.91 ± 0.86 | NA | NA | NA | |
|  | Black | 0.65–2.04  (n = 3) |  |  | 68.07–140.1 (n = 3) | 4.79–7.94  (n = 3) |  | 0.03–13.68 (n = 15) |  | |  |  |  |  | |
|  | Black | 1.30 ± 0.70 | NA | NA | 106.0 ± 36.17 | 6.24 ± 1.59 | 112.2 ± 37.76 | 6.57 ± 3.39 | NA | | NA | NA | NA | NA | |
|  | Red | 1.48–3.13  (n = 4) |  |  | 17.35–192.9 (n = 2) |  |  | 0.06–72.40 (n = 13) |  | |  |  |  |  | |
|  | Red | 2.47 ± 0.78 | NA | NA | 105.1 ± 124.1 | NA | NA | 18.97 ± 21.83 | NA | | NA | NA | NA | NA | |
|  | Purple | 0.80  (n = 1) |  |  |  |  |  | 3.53–7.10  (n = 2) |  | |  |  |  |  | |
|  | Purple | 0.80 ± 0.00 | NA | NA | NA | NA | NA | 5.32 ± 2.52 | NA | | NA | NA | NA | NA | |
|  | Pigmented | 0.65–3.13  (n = 8) |  |  | 17.35–192.9 (n = 5) | 4.79–7.94  (n = 3) |  | 1.80–72.40 (n = 30) |  | |  |  |  |  | |
|  | Pigmented | 1.52 ± 0.49 | NA | NA | 105.5 ± 80.15 | 6.24 ± 1.59 | 111.8 ± 81.74 | 10.28 ± 9.25 | NA | | NA | NA | NA | NA | |
|  |  |  |  |  |  |  |  |  |  | |  |  |  |  | |
| Inhibition of lipid peroxidation (conjugated dienes, EC50, mg/mL) | Brown | 0.26  (n = 1) |  |  | 0.14–0.62  (n = 4) |  |  | 0.32–0.71  (n = 2) |  | |  |  |  |  | |
|  | Brown | 0.26 ± 0.00 | NA | NA | 0.41 ± 0.22 | NA | NA | 0.52 ± 0.28 | NA | | NA | NA | NA | NA | |
|  | Red |  |  |  |  |  |  |  |  | |  |  |  |  | |
|  | Red | NA | NA | NA | NA | NA | NA | NA | NA | | NA | NA | NA | NA | |
|  | Purple |  |  |  |  |  |  |  |  | |  |  |  |  | |
|  | Purple | NA | NA | NA | NA | NA | NA | NA | NA | | NA | NA | NA | NA | |
|  | Pigmented |  |  |  |  |  |  |  |  | |  |  |  |  | |
|  | Pigmented | NA | NA | NA | NA | NA | NA | NA | NA | | NA | NA | NA | NA | |
|  |  |  |  |  |  |  |  |  |  | |  |  |  |  | |
|  |  |  |  |  |  |  |  |  |  | |  |  |  |  | |
| ABTS (Mm trolox Eq/100 g) | Brown | 0.02–4.93  (n = 4) | 0.19–3.47 (n = 2) |  | 1.18–14.23  (n = 6) | 6.98  (n = 1) |  | 0.02–2.02 (n = 22) | 0.02–1.12  (n = 3) | |  | 0.20  (n = 1) | 2.08  (n = 1) |  | |
|  | Brown | 2.44 ± 1.27 | 2.32 ± 1.83 | 4.76 ± 3.10 | 5.24 ± 4.20 | 6.98 ± 0.00 | 12.22 ± 5.24 | 0.65 ± 0.69 | 0.57 ± 0.47 | | 1.22 ± 1.16 | 0.20 ± 0.00 | 2.08 ± 0.00 | 2.28 ± 0.00 | |
|  | Black |  |  |  |  |  |  | 1.45–12.03 (n = 11) | 0.21  (n = 1) | |  |  |  |  | |
|  | Black | NA | NA | NA | NA | NA | NA | 4.68 ± 2.93 | 0.21 ± 0.00 | | 4.89 ± 2.93 | NA | NA | NA | |
|  | Red | 0.05–0.09  (n = 4) |  |  | 8.67–14.23  (n = 4) |  |  | 0.29–12.29 (n = 14) | 0.15  (n = 1) | |  |  |  |  | |
|  | Red | 0.07 ± 0.02 | NA | NA | 11.31 ± 2.47 | NA | NA | 2.82 ± 3.28 | 0.15 ± 0.00 | | 2.97 ± 3.28 | NA | NA | NA | |
|  | Purple |  |  |  | 4.37–20.97  (n = 3) |  |  |  |  | |  |  |  |  | |
|  | Purple | NA | NA | NA | 13.45 ± 8.41 | NA | NA | NA | NA | | NA | NA | NA | NA | |
|  | Pigmented | 0.05–0.09  (n = 4) |  |  | 4.37–20.97  (n = 7) |  |  | 0.29–12.29 (n = 25) | 0.15–0.21  (n = 2) | |  |  |  |  | |
|  | Pigmented | 0.07 ± 0.02 | NA | NA | 12.38 ± 5.44 | NA | NA | 3.75 ± 3.10 | 0.18 ± 0.00 | | 3.93 ± 3.10 | NA | NA | NA | |
|  |  |  |  |  |  |  |  |  |  | |  |  |  |  | |
|  |  |  |  |  |  |  |  |  |  | |  |  |  |  | |
| ABTS (%) | Brown |  |  |  | 42.30–49.50 (n = 3) |  |  | 30.00–56.07 (n = 5) | 96.88  (n = 1) | |  |  |  |  | |
|  | Brown | NA | NA | NA | 45.27 ± 3.76 | NA | NA | 35.14 ± 16.28 | 96.88 ± 0.00 | |  | NA | NA | NA | |
|  | Black |  |  |  |  |  |  | 58.98–80.00 (n = 6) |  | |  |  |  |  | |
|  | Black | NA | NA | NA | NA | NA | NA | 69.83 ± 9.18 | NA | | NA | NA | NA | NA | |
|  | Red |  |  |  |  |  |  | 25.00–63.20 (n = 9) |  | |  |  |  |  | |
|  | Red | NA | NA | NA | NA | NA | NA | 47.94 ± 11.03 | NA | | NA | NA | NA | NA | |
|  | Purple |  |  |  |  |  |  |  |  | |  |  |  |  | |
|  | Purple | NA | NA | NA | NA | NA | NA | NA | NA | | NA | NA | NA | NA | |
|  | Pigmented |  |  |  |  |  |  | 25.00–80.00 (n = 15) |  | |  |  |  |  | |
|  | Pigmented | NA | NA | NA | NA | NA | NA | 58.89 ± 10.11 | NA | | NA | NA | NA | NA | |
|  |  |  |  |  |  |  |  |  |  | |  |  |  |  | |
| ABTS (EC50, mg/mL) | Brown |  |  |  | 0.04–3.21  (n = 3) |  |  |  |  | |  |  |  |  | |
|  | Brown | NA | NA | NA | 1.70 ± 1.42 | NA | NA | NA | NA | | NA | NA | NA | NA | |
|  | Black |  |  |  | 0.44  (n = 1) |  |  |  |  | |  |  |  |  | |
|  | Black | NA | NA | NA | 0.44 ± 0.00 | NA | NA | NA | NA | | NA | NA | NA | NA | |
|  | Red |  |  |  | 0.33  (n = 1) |  |  |  |  | |  |  |  |  | |
|  | Red | NA | NA | NA | 0.33 ± 0.00 | NA | NA | NA | NA | | NA | NA | NA | NA | |
|  | Purple |  |  |  |  |  |  |  |  | |  |  |  |  | |
|  | Purple | NA | NA | NA | NA | NA | NA | NA | NA | | NA | NA | NA | NA | |
|  | Pigmented |  |  |  | 0.77  (n = 2) |  |  |  |  | |  |  |  |  | |
|  | Pigmented | NA | NA | NA | 0.77 ± 0.00 | NA | NA | NA | NA | | NA | NA | NA | NA | |
|  |  |  |  |  |  |  |  |  |  | |  |  |  |  | |
| Reducing power (absorbance) | Brown | 0.05–0.29  (n = 4) |  |  | 0.10–1.85  (n = 10) |  |  | 0.13–0.35 (n = 10) |  | |  | 0.35  (n = 1) |  |  | |
|  | Brown | 0.15 ± 0.10 | NA | NA | 0.93 ± 0.59 | NA | NA | 0.24 ± 0.08 | NA | | NA | 0.35 ± 0.00 | NA | NA | |
|  | Black |  |  |  | 0.25–2.06  (n = 5) |  |  | 0.25  (n = 1) |  | |  |  |  |  | |
|  | Black | NA | NA | NA | 1.04 ± 0.76 | NA | NA | 0.25 ± 0.00 | NA | | NA | NA | NA | NA | |
|  | Red |  |  |  | 0.17–2.92  (n = 5) |  |  | 0.47  (n = 1) |  | |  |  |  |  | |
|  | Red | NA | NA | NA | 1.23 ± 1.15 | NA | NA | 0.47 ± 0.00 | NA | | NA | NA | NA | NA | |
|  | Purple |  |  |  | 1.50  (n = 1) |  |  | 0.72  (n = 1) |  | |  |  |  |  | |
|  | Purple | NA | NA | NA | 1.50 ± 0.00 | NA | NA | 0.72 ± 0.00 | NA | | NA | NA | NA | NA | |
|  | Pigmented |  |  |  | 0.17–2.92  (n = 10) |  |  | 0.25–0.72  (n = 3) |  | |  |  |  |  | |
|  | Pigmented | NA | NA | NA | 1.26 ± 0.64 | NA | NA | 0.48 ± 0.00 | NA | | NA | NA | NA | NA | |
|  |  |  |  |  |  |  |  |  |  | |  |  |  |  | |
| FRAP (mM Fe+/trolox Eq/100 g) | Brown | 0.05–0.65  (n = 9) | 0.73  (n = 1) |  | 1.46–5.72  (n = 11) |  |  | 0.35–1.83 (n = 17) | 0.02–1.15  (n = 4) | |  | 1.24–2.82 (n = 3) | 5.06  (n = 1) |  | |
|  | Brown | 0.30 ± 0.25 | 0.73 ± 0.00 | 1.03 ± 0.25 | 3.05 ± 1.17 | NA | NA | 0.87 ± 0.54 | 0.39 ± 0.51 | | 1.26 ± 1.06 | 2.02 ± 0.79 | 5.06 ± 0.00 | 7.08 ± 0.79 | |
|  | Black |  |  |  |  |  |  | 0.62–12.61 (n = 12) | 0.10–0.22  (n = 3) | |  |  |  |  | |
|  | Black | NA | NA | NA | NA | NA | NA | 4.71 ± 4.56 | 0.16 ± 0.06 | | 4.87 ± 4.62 | NA | NA | NA | |
|  | Red |  |  |  | 4.07–17.22  (n = 7) |  |  | 0.15–8.08 (n = 11) | 1.98–3.42  (n = 3) | |  |  |  |  | |
|  | Red | NA | NA | NA | 11.01 ± 4.31 | NA | NA | 2.43 ± 2.60 | 2.59 ± 0.75 | | 5.02 ± 3.35 | NA | NA | NA | |
|  | Purple |  |  |  | 3.22–7.19  (n = 3) |  |  | 2.59–3.29  (n = 2) | 0.07  (n = 1) | |  |  |  |  | |
|  | Purple | NA | NA | NA | 5.14 ± 1.99 | NA | NA | 2.90 ± 0.56 | 0.07 ± 0.00 | | 2.97 ± 0.56 | NA | NA | NA | |
|  | Pigmented |  |  |  | 3.22–17.22 (n = 10) |  |  | 0.15–12.61 (n = 25) | 0.07–3.42  (n = 7) | |  |  |  |  | |
|  | Pigmented | NA | NA | NA | 8.07 ± 3.15 | NA | NA | 3.35 ± 2.57 | 0.94 ± 0.27 | | 4.29 ± 2.84 | NA | NA | NA | |
|  |  |  |  |  |  |  |  |  |  | |  |  |  |  | |
| Ferrous ion–chelating activity (mg EDTA Eq/100 g) | Brown | 10.00–150.40 (n = 4) |  |  | 610.1–1250  (n = 5) |  |  | 159.8–420.0 (n = 4) | 141.0  (n = 1) | |  |  |  |  | |
|  | Brown | 67.53 ± 59.29 | NA | NA | 893.0 ± 306.1 | NA | NA | 250.5 ± 115.9 | 141.0 ± 0.00 | | 391.5 ± 115.9 | NA | NA | NA | |
|  | Black |  |  |  |  |  |  |  |  | |  |  |  |  | |
|  | Black | NA | NA | NA | NA | NA | NA | NA | NA | | NA | NA | NA | NA | |
|  | Red |  |  |  | 2474.0  (n = 1) | 162.0  (n = 1) |  | 1721  (n = 1) | 451.0  (n = 1) | |  |  |  |  | |
|  | Red | NA | NA | NA | 2474 ± 0.00 | 162.0 ± 0.00 | 2636 ± 0.00 | 1721 ± 0.00 | 451.0 ± 0.00 | | 2172 ± 0.00 | NA | NA | NA | |
|  | Purple |  |  |  | 1534  (n = 1) | 204.0  (n = 1) |  | 1279  (n = 1) | 443.0  (n = 1) | |  |  |  |  | |
|  | Purple | NA | NA | NA | 1534 ± 0.00 | 204.000 ± 0.00 | 1738 ± 0.00 | 1279 ± 0.00 | 443.0 ± 0.00 | | 1722 ± 0.00 | NA | NA | NA | |
|  | Pigmented |  |  |  | 1534–2474 (n = 2) | 162.0–204.0 (n = 2) |  | 1279–1721 (n = 2) | 443.0–452.0 (n = 2) | |  |  |  |  | |
|  | Pigmented | NA | NA | NA | 2004 ± 102.0 | 183.0 ± 0.00 | 2187 ± 102.0 | 1083 ± 36.64 | 447.0 ± 0.00 | | 1530 ± 38.64 | NA | NA | NA | |
|  |  |  |  |  |  |  |  |  |  | |  |  |  |  | |
| Ferrous ion–chelating activity (%) | Brown |  |  |  |  |  |  | 14.33–110.45 (n = 4) |  | |  |  |  |  | |
|  | Brown | NA | NA | NA | NA | NA | NA | 48.66 ± 42.51 | NA | | NA | NA | NA | NA | |
|  | Black |  |  |  | 8.11  (n = 1) |  |  |  |  | |  |  |  |  | |
|  | Black | NA | NA | NA | 8.11 ± 0.00 | NA | NA | NA | NA | | NA | NA | NA | NA | |
|  | Red |  |  |  | 4.25  (n = 1) |  |  |  |  | |  |  |  |  | |
|  | Red | NA | NA | NA | 4.25 ± 0.00 | NA | NA | NA | NA | | NA | NA | NA | NA | |
|  | Purple |  |  |  | 5.42  (n = 1) |  |  |  |  | |  |  |  |  | |
|  | Purple | NA | NA | NA | 5.42 ± 0.00 | NA | NA | NA | NA | | NA | NA | NA | NA | |
|  | Pigmented |  |  |  | 4.25–8.11  (n = 3) |  |  |  |  | |  |  |  |  | |
|  | Pigmented | NA | NA | NA | 5.93 ± 0.00 | NA | NA | NA | NA | | NA | NA | NA | NA | |
|  |  |  |  |  |  |  |  |  |  | |  |  |  |  | |
| Ferrous ion–chelating activity (EC50, mg/mL) | Brown | 5.26  (n = 1) |  |  | 0.11–1.50  (n = 5) |  |  | 1.41–7.09  (n = 2) |  | |  | 0.35  (n = 1) |  |  | |
|  | Brown | 5.26 ± 0.00 | NA | NA | 0.68 ± 0.55 | NA | NA | 4.25 ± 4.02 | NA | | NA | 0.35 ± 0.00 | NA | NA | |
|  | Black |  |  |  |  |  |  |  |  | |  |  |  |  | |
|  | Black | NA | NA | NA | NA | NA | NA | NA | NA | | NA | NA | NA | NA | |
|  | Red |  |  |  |  |  |  |  |  | |  |  |  |  | |
|  | Red | NA | NA | NA | NA | NA | NA | NA | NA | | NA | NA | NA | NA | |
|  | Purple |  |  |  |  |  |  |  |  | |  |  |  |  | |
|  | Purple | NA | NA | NA | NA | NA | NA | NA | NA | | NA | NA | NA | NA | |
|  | Pigmented |  |  |  |  |  |  |  |  | |  |  |  |  | |
|  | Pigmented | NA | NA | NA | NA | NA | NA | NA | NA | | NA | NA | NA | NA | |
|  |  |  |  |  |  |  |  |  |  | |  |  |  |  | |
|  |  |  |  |  |  |  |  |  |  | |  |  |  |  | |
| TBARS (mg TBARS Eq/100g) | Brown |  |  |  | 0.04–2.80  (n = 5) |  |  |  |  | |  | 3.84 (n = 1) |  |  | |
|  | Brown | NA | NA | NA | 1.08 ± 1.39 | NA | NA | NA | NA | | NA | 3.84 ± 0.00 | NA | NA | |
|  | Black |  |  |  |  |  |  |  |  | |  |  |  |  | |
|  | Black | NA | NA | NA | NA | NA | NA | NA | NA | | NA | NA | NA | NA | |
|  | Red |  |  |  |  |  |  |  |  | |  |  |  |  | |
|  | Red | NA | NA | NA | NA | NA | NA | NA | NA | | NA | NA | NA | NA | |
|  | Purple |  |  |  |  |  |  |  |  | |  |  |  |  | |
|  | Purple | NA | NA | NA | NA | NA | NA | NA | NA | | NA | NA | NA | NA | |
|  | Pigmented |  |  |  |  |  |  |  |  | |  |  |  |  | |
|  | Pigmented | NA | NA | NA | NA | NA | NA | NA | NA | | NA | NA | NA | NA | |
|  |  |  |  |  |  |  |  |  |  | |  |  |  |  | |
| Hydroxyl radical scavenging activity (%) | Brown | 20.11–42.35 (n = 6) |  |  | 79.36  (n = 1) |  |  | 21.80–73.97 (n = 7) |  | |  |  |  |  | |
|  | Brown | 28.71 ± 9.66 | NA | NA | 79.36 ± 0.00 | NA | NA | 54.05 ± 16.02 | NA | | NA | NA | NA | NA | |
|  | Black |  |  |  | 71.17  (n = 1) |  |  | 52.57  (n = 1) |  | |  |  |  |  | |
|  | Black | NA | NA | NA | 71.17 ± 0.00 | NA | NA | 52.77 ± 0.00 | NA | | NA | NA | NA | NA | |
|  | Red |  |  |  | 81.66  (n = 1) |  |  |  |  | |  |  |  |  | |
|  | Red | NA | NA | NA | 81.66 ± 0.00 | NA | NA | NA | NA | | NA | NA | NA | NA | |
|  | Purple |  |  |  |  |  |  | 29.82–40.80 (n = 2) |  | |  |  |  |  | |
|  | Purple | NA | NA | NA | NA | NA | NA | 35.31 ± 7.76 | NA | | NA | NA | NA | NA | |
|  | Pigmented |  |  |  | 71.17–81.66 (n = 2) |  |  | 29.82–52.57 (n = 3) |  | |  |  |  |  | |
|  | Pigmented | NA | NA | NA | 76.42 ± 0.00 | NA | NA | 44.04 ± 3.88 | NA | | NA | NA | NA | NA | |
|  |  |  |  |  |  |  |  |  |  | |  |  |  |  | |
|  |  |  |  |  |  |  |  |  |  | |  |  |  |  | |
| Hydroxyl radical scavenging activity (EC50, mg/mL) | Brown | 2.10–6.44  (n = 3) |  |  | 11.46–5.43  (n = 6) |  |  | 4.90–7.34  (n = 2) |  | |  | ND  (n = 1) |  |  | |
|  | Brown | 4.18 ± 2.18 | NA | NA | 3.94 ± 1.67 | NA | NA | 6.12 ± 1.73 | NA | | NA | ND | ND | ND | |
|  | Black |  |  |  | 0.09–1.45  (n = 8) |  |  |  |  | |  |  |  |  | |
|  | Black | NA | NA | NA | 1.18 ± 0.54 | NA | NA | NA | NA | | NA | NA | NA | NA | |
|  | Red |  |  |  | 0.57–3.57  (n = 7) |  |  |  |  | |  |  |  |  | |
|  | Red | NA | NA | NA | 1.57 ± 1.04 | NA | NA | NA | NA | | NA | NA | NA | NA | |
|  | Purple |  |  |  |  |  |  |  |  | |  |  |  |  | |
|  | Purple | NA | NA | NA | NA | NA | NA | NA | NA | | NA | NA | NA | NA | |
|  | Pigmented |  |  |  | 0.09–3.57  (n = 15) |  |  |  |  | |  |  |  |  | |
|  | Pigmented | NA | NA | NA | 1.37 ± 0.79 | NA | NA | NA | NA | | NA | NA | NA | NA | |
|  |  |  |  |  |  |  |  |  |  | |  |  |  |  | |
| Hydrogen peroxide scavenging assay (EC50, mg/mL) | Brown |  |  |  |  |  |  |  |  | |  | 5.82  (n = 1) |  |  | |
|  | Brown | NA | NA | NA | NA | NA | NA | NA | NA | | NA | 5.82 ± 0.00 | NA | NA | |
|  | Black |  |  |  |  |  |  |  |  | |  |  |  |  | |
|  | Black | NA | NA | NA | NA | NA | NA | NA | NA | | NA | NA | NA | NA | |
|  | Red |  |  |  |  |  |  |  |  | |  |  |  |  | |
|  | Red | NA | NA | NA | NA | NA | NA | NA | NA | | NA | NA | NA | NA | |
|  | Purple |  |  |  |  |  |  |  |  | |  |  |  |  | |
|  | Purple | NA | NA | NA | NA | NA | NA | NA | NA | | NA | NA | NA | NA | |
|  | Pigmented |  |  |  |  |  |  |  |  | |  |  |  |  | |
|  | Pigmented | NA | NA | NA | NA | NA | NA | NA | NA | | NA | NA | NA | NA | |
|  |  |  |  |  |  |  |  |  |  | |  |  |  |  | |
| Superoxide radical scavenging activity (%) | Brown | 31.99–42.61 (n = 3) |  |  | 59.21  (n = 1) |  |  | 16.70–76.88 (n = 4) |  | |  |  |  |  | |
|  | Brown | 37.53 ± 5.33 | NA | NA | 59.21 ± 0.00 | NA | NA | 49.48 ± 25.58 | NA | | NA | NA | NA | NA | |
|  | Black |  |  |  | 85.75  (n = 1) |  |  | 35.00–78.12 (n = 4) |  | |  |  |  |  | |
|  | Black | NA | NA | NA | 85.75 ± 0.00 | NA | NA | 47.78 ± 20.33 | NA | | NA | NA | NA | NA | |
|  | Red |  |  |  | 41.19–89.75 (n = 5) |  |  | 15.44–61.40 (n = 4) |  | |  |  |  |  | |
|  | Red | NA | NA | NA | 73.53 ± 20.95 | NA | NA | 32.46 ± 20.19 | NA | | NA | NA | NA | NA | |
|  | Purple |  |  |  | 16.02–26.80 (n = 3) |  |  | 27.88–90.30 (n = 2) |  | |  |  |  |  | |
|  | Purple | NA | NA | NA | 21.61 ± 5.40 | NA | NA | 59.09 ± 44.14 | NA | | NA | NA | NA | NA | |
|  | Pigmented |  |  |  | 16.02–89.75 (n = 9) |  |  | 15.44–90.30 (n = 10) |  | |  |  |  |  | |
|  | Pigmented | NA | NA | NA | 60.29 ± 8.78 | NA | NA | 46.44 | NA | | NA | NA | NA | NA | |
|  |  |  |  |  |  |  |  |  |  | |  |  |  |  | |
| Superoxide radical scavenging activity (EC50, mg/mL) | Brown |  |  |  | 0.02–2.09  (n = 7) |  |  | 1.22  (n = 1) |  | |  | 1.56  (n = 1) |  |  | |
|  | Brown | NA | NA | NA | 0.96 ± 0.76 | NA | NA | 1.22 ± 0.00 | NA | | NA | 1.56 ± 0.00 | NA | NA | |
|  | Black |  |  |  | 0.02–0.60  (n = 8) |  |  |  |  | |  |  |  |  | |
|  | Black | NA | NA | NA | 0.21 ± 0.25 | NA | NA | NA | NA | | NA | NA | NA | NA | |
|  | Red |  |  |  | 0.02–0.52  (n = 7) |  |  |  |  | |  |  |  |  | |
|  | Red | NA | NA | NA | 0.19 ± 0.18 | NA | NA | NA | NA | | NA | NA | NA | NA | |
|  | Purple |  |  |  |  |  |  |  |  | |  |  |  |  | |
|  | Purple | NA | NA | NA | NA | NA | NA | NA | NA | | NA | NA | NA | NA | |
|  | Pigmented |  |  |  | 0.02–0.60  (n = 15) |  |  |  |  | |  |  |  |  | |
|  | Pigmented | NA | NA | NA | 0.20 ± 0.22 | NA | NA | NA | NA | | NA | NA | NA | NA | |
|  |  |  |  |  |  |  |  |  |  | |  |  |  |  | |
| Oxygen radical scavenging activity (EC50, mg/mL) | Brown |  |  |  | 1.38–6.12  (n = 4) |  |  |  |  | |  |  |  |  | |
|  | Brown | NA | NA | NA | 3.14 ± 2.10 | NA | NA | NA | NA | | NA | NA | NA | NA | |
|  | Black |  |  |  | 0.09–2.36  (n = 7) |  |  |  |  | |  |  |  |  | |
|  | Black | NA | NA | NA | 1.04 ± 0.92 | NA | NA | NA | NA | | NA | NA | NA | NA | |
|  | Red |  |  |  | 0.15–1.57  (n = 6) |  |  |  |  | |  |  |  |  | |
|  | Red | NA | NA | NA | 0.66 ± 0.56 | NA | NA | NA | NA | | NA | NA | NA | NA | |
|  | Purple |  |  |  |  |  |  |  |  | |  |  |  |  | |
|  | Purple | NA | NA | NA | NA | NA | NA | NA | NA | | NA | NA | NA | NA | |
|  | Pigmented |  |  |  | 0.09–2.36  (n = 13) |  |  |  |  | |  |  |  |  | |
|  | Pigmented | NA | NA | NA | 0.85 ± 0.74 | NA | NA | NA | NA | | NA | NA | NA | NA | |
|  |  |  |  |  |  |  |  |  |  | |  |  |  |  | |
| Reduction of molybdate (mg BHT Eq/100 g) | Brown |  |  |  | 217.0–1504 (n = 8) |  |  |  |  | |  |  |  |  | |
|  | Brown | NA | NA | NA | 947.3 ± 499.8 | NA | NA | NA | NA | | NA | NA | NA | NA | |
|  | Black |  |  |  | 2900–4500 (n = 4) |  |  |  |  | |  |  |  |  | |
|  | Black | NA | NA | NA | 3475 ± 732.0 | NA | NA | NA | NA | | NA | NA | NA | NA | |
|  | Red |  |  |  | 2592  (n = 1) |  |  |  |  | |  |  |  |  | |
|  | Red | NA | NA | NA | 2592 ± 0.00 | NA | NA | NA | NA | | NA | NA | NA | NA | |
|  | Purple |  |  |  |  |  |  |  |  | |  |  |  |  | |
|  | Purple | NA | NA | NA | NA | NA | NA | NA | NA | | NA | NA | NA | NA | |
|  | Pigmented |  |  |  | 2592–4500 (n = 4) |  |  |  |  | |  |  |  |  | |
|  | Pigmented | NA | NA | NA | 3033 ± 366.0 | NA | NA | NA | NA | | NA | NA | NA | NA | |
|  |  |  |  |  |  |  |  |  |  | |  |  |  |  | |
| Tert–butylperoxyl radical scavenging activity (EC50, mg/mL) | Brown |  |  |  | 0.32–0.46  (n = 4) |  |  | 1.01  (n = 1) |  | |  |  |  |  | |
|  | Brown | NA | NA | NA | 0.40 ± 0.06 | NA | NA | 1.01 ± 0.00 | NA | | NA | NA | NA | NA | |
|  | Black |  |  |  | 0.09–0.71  (n = 7) |  |  | 1.30  (n = 1) |  | |  |  |  |  | |
|  | Black | NA | NA | NA | 0.32 ± 0.22 | NA | NA | 1.30 ± 0.00 | NA | | NA | NA | NA | NA | |
|  | Red |  |  |  | 0.09–0.79  (n = 6) |  |  | 1.80  (n = 1) |  | |  |  |  |  | |
|  | Red | NA | NA | NA | 0.42 ± 0.26 | NA | NA | 1.80 ± 0.00 | NA | | NA | NA | NA | NA | |
|  | Purple |  |  |  |  |  |  |  |  | |  |  |  |  | |
|  | Purple | NA | NA | NA | NA | NA | NA | NA | NA | | NA | NA | NA | NA | |
|  | Pigmented |  |  |  | 0.09–0.79 (n = 13) |  |  | 1.30–1.80  (n = 2) |  | |  |  |  |  | |
|  | Pigmented | NA | NA | NA | 0.37 ± 0.24 | NA | NA | | 1.55 ± 0.00 | NA | | NA | NA | NA | NA |
|  |  |  |  |  |  |  |  | |  |  | |  |  |  |  |

^a^ For each parameter, the first raw values describe the minimum and maximum values (A–B) and the total number of studies from which data were extracted (n), whereas the second raw values show the mean and SD.

^b^ NA = not available.

^c^ ND = not detected.

^d^ Pigmented rice refer to rice with black, purple, and red bran, whereas non-pigmented rice refer to rice with brown bran.
